# Asymmetric α-amination of 3-substituted oxindoles using chiral bifunctional phosphine catalysts

**DOI:** 10.3762/bjoc.12.72

**Published:** 2016-04-15

**Authors:** Qiao-Wen Jin, Zhuo Chai, You-Ming Huang, Gang Zou, Gang Zhao

**Affiliations:** 1 Laboratory of Advanced Materials and Institute of Fine Chemicals, East China University of Science and Technology, 130 Meilong Road, Shanghai 200237, People’s Republic of China; 2 Key Laboratory of Synthetic Chemistry of Natural Substances, Shanghai Institute of Organic Chemistry, Chinese Academy of Sciences, 345 Lingling Road, Shanghai 200032, People’s Republic of China

**Keywords:** 3-aminooxindoles, asymmetric catalysis, phosphine catalyst, tetrasubstituted stereogenic carbon centers

## Abstract

A highly enantioselective α-amination of 3-substituted oxindoles with azodicarboxylates catalyzed by amino acids-derived chiral phosphine catalysts is reported. The corresponding products containing a tetrasubstituted carbon center attached to a nitrogen atom at the C-3 position of the oxindole were obtained in high yields and with up to 98% ee.

## Introduction

Recently, chiral 3-substituted oxindoles have been attractive targets in asymmetric synthesis due to their abundance in the structures of numerous natural products and pharmaceutically active compounds [1]. In particular, the chiral 3-aminooxindoles containing a tetrasubstituted carbon center have been recognized as core building blocks for the preparation of many biologically active and therapeutic compounds [2–7]. As a type of commercially available electrophilic amination reagents, azodicarboxylates have been extensively used in both asymmetric organocatalysis and metal catalysis for the construction of this type of structures. For example, Chen et al. reported the first organocatalytic enantioselective amination reaction of 2-oxindoles catalyzed by biscinchona alkaloid catalysts [8]. Zhou [9–10] and Barbas [11–12], have independently reported similar organocatalytic processes. In the field of metal catalysis, Shibasaki et al. reported the reaction between C3-substituted oxindole and azodicarboxylates, using homodinuclear or monometallic Ni-Schiff base complexes as catalysts [13]; Feng et al. also developed a similar procedure with chiral *N*,*N’*-dioxide-Sc(III) complexes as catalysts [14]. Despite these impressive advances, current catalytic systems still more or less suffer from limitations such as long reaction times, relatively large catalyst loading in most organocatalytic systems and in some cases unsatisfactory yields and/or enantioselectivities. Therefore, the development of more efficient catalytic systems for the asymmetric α-amination of 3-substituted oxindoles with azodicarboxylates is still desirable.

**Scheme 1:**
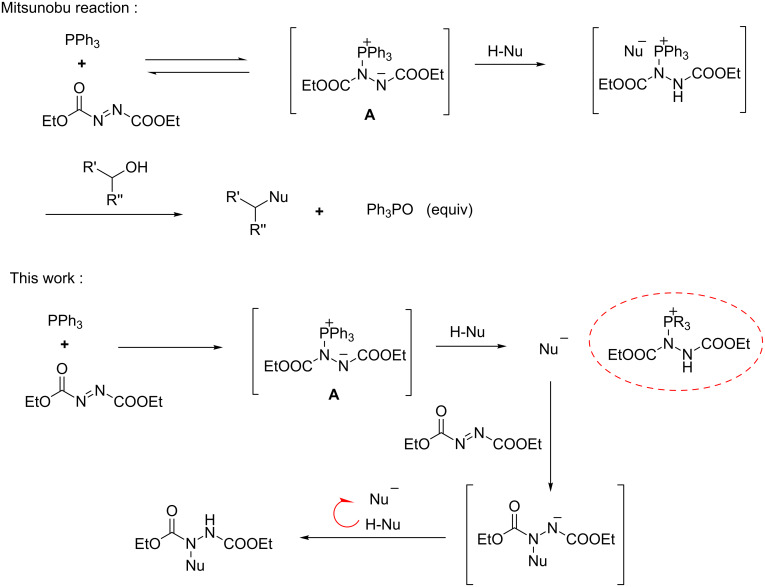
The mimetic activation mode of Mitsunobu reaction.

Chiral organophosphine catalysis [15–18] has captured considerable attention over the past decades owing to its high catalytic efficiency in a variety of reactions such as aza-Morita–Baylis–Hillman reactions [19–21], Rauhut–Currier reactions [22–27], Michael addition reactions [28–35], and various cycloadditions [36–39]. In recent years, our group has focused on the development of novel amino acid-derived chiral bifunctional organophosphine catalysts, which have successfully applied to catalyze various asymmetric reactions [40–41]. As a general concept, a tertiary phosphine adds to an electrophilic reactant to form a zwitterion which serves as either a nucleophile or a Bronsted base to participate in the catalytic cycle. In 2015, we reported a novel asymmetric dual-reagent catalysis strategy based on these chiral phosphine catalysts [42], in which the zwitterion in situ generated from the chiral phosphine and methyl acrylate acted as an efficient catalyst for the asymmetric Mannich-type reaction. As an extension of this work, we then wondered if other electrophilic partners instead of methyl acrylate could be used to generate similar catalytically active species in situ. Also inspired by the Mitsunobu reaction [43], we reported herein the reaction of azodicarboxylates with 3-substituted oxindole catalyzed by chiral amino acid-derived organophosphine catalysts, in which the zwitterions in situ generated from the phosphine and azodicarboxylates serve as highly efficient catalysts [44] (Scheme 1).

## Results and Discussion

Initially, the reaction between 3-phenyloxindole **1a** and DEAD (diethyl azodicarboxylate, **2a**) was selected as the model reaction for the evaluation of chiral phosphine catalysts (Table 1). Using bifunctional thiourea catalysts **4a** and **4b**, the reaction proceeded smoothly at room temperature to afford the product **3a** in good yields, albeit with low enantiomeric excesses (ee) (Table 1, entries 1 and 2). When the thiourea moiety in the catalysts were replaced by amides, the enantioselectivities were greatly improved (Table 1, entries 3–6). The examination of catalysts **4c–f** with further fine-tuning on the acyl group revealed **4d** as the optimal catalyst for this transformation (Table 1, entry 4). Different from *N*-Boc-oxindole, using *N*-unprotected oxindole and *N*-benzyl-substituted oxindole as the substrates, accomplished the reaction with every low enantioselectivity (Table 1, entries 7 and 8), incidated the *N*-Boc protecting group is crucial for this system.

**Table 1:** Catalyst screening.

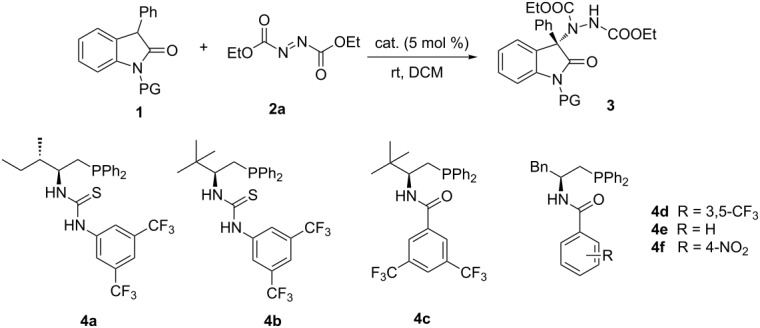					

Entry^a^	PG	Catalyst	*t* (min)	Yield (%)^b^	ee (%)^c^

1	Boc (**1a**)	**4a**	5	72	17
2	Boc (**1a**)	**4b**	5	79	39
3	Boc (**1a**)	**4c**	5	89	61
4	Boc (**1a**)	**4d**	5	85	83
5	Boc (**1a**)	**4e**	5	88	60
6	Boc (**1a**)	**4f**	5	70	64
7	H (**1b**)	**4d**	40	66	17
8	Bn (**1c**)	**4d**	60	50	0

^a^0.1 mmol scale in 1.0 mL of DCM. ^b^Isolated yield. ^c^Determined by chiral HPLC analysis.

Next, the influence of solvents and reaction temperature on the reaction were investigated with the best catalyst (Table 2). The use of both polar solvents (ethyl ether, tetrahydrofuran, acetone, acetonitrile or ethyl alcohol) including other chlorinated solvents such as chloroform, 1,2-dichloroethane and 1,1,2-trichloroethane or the less polar solvent toluene gave no improvement in enantioselectivity in comparison to the originally used DCM (Table 2, entries 1–9). To our delight, lowering the reaction temperature increased the reaction yield significantly (Table 2, entries 10–16), while the highest ee value (90%) with DEAD was obtained at −30 °C (Table 2, entry 13). Interestingly, the use of other azodicarboxylates with larger R group as amination reagent revealed different optimum reaction temperatures for the best enantioselectivity, and −78 °C was identified as optimal for di-*tert*-butyl azodicarboxylate (**2d**, DBAD, 93% ee, Table 2, entry 20). It’s worth mentioning that the reaction could still proceed to completion within 5 minutes under such low reaction temperatures.

**Table 2:** Optimization of conditions.

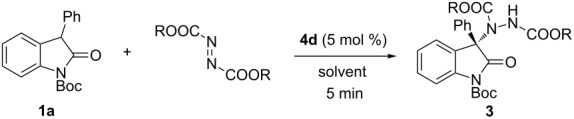					

Entry^a^	R	Solvent	*T* (°C)	Yield (%)^b^	ee (%)^c^

1	Et	Et_2_O	rt	64 (**3a**)	65
2	Et	THF	rt	62 (**3a**)	39
3	Et	acetone	rt	62 (**3a**)	35
4	Et	acetonitrile	rt	41 (**3a**)	5
5	Et	EtOH	rt	78 (**3a**)	0
6	Et	toluene	rt	70 (**3a**)	79
7	Et	CHCl_3_	rt	66 (**3a**)	79
8	Et	1,2-dichloroethane	rt	66 (**3a**)	77
9	Et	1,1,2-trichloroethane	rt	74 (**3a**)	53
10	Et	DCM	0	81 (**3a**)	83
11	Et	DCM	−10	93 (**3a**)	84
12	Et	DCM	−20	86 (**3a**)	85
13	Et	DCM	−30	87 (**3a**)	90
14	Et	DCM	−40	87 (**3a**)	84
15	Et	DCM	−50	93 (**3a**)	81
16	Et	DCM	−78	85 (**3a**)	68
17	iPr	DCM	−30	95 (**3b**)	82
18	iPr	DCM	−78	93 (**3b**)	89
19	*t*-Bu	DCM	−30	87 (**3d**)	64
20	*t*-Bu	DCM	−78	80 (**3d**)	93

^a^0.1 mmol scale in 1.0 mL of solvent. ^b^Isolated yield. ^c^Determined by chiral HPLC analysis.

With the optimized reaction conditions in hand, a variety of oxindoles **1** and azodicarboxylates **2** were then examined to probe the scope of the reaction (Table 3). In general, the catalytic system showed excellent efficiency for all the substrates examined to provide good to excellent yields and enantioselectivities within a very short reaction time (5 min). The use of the sterically more hindered DBAD is much more favored than DEAD in terms of enantioselectivity. The substitution type including different electronic nature and/or positions of the substituents on the benzene ring of the oxoindole skeleton or 3-aryl group showed no pronounced influence on the reaction in terms of both yield and enantioselectivity. It is noteworthy that products **3i**, **3q**, **3r** and **3s**, which contain a fluorine atom, were obtained in good yield with good to execellent ee (Table 3, entries 6 and 14–16). Enantiomerically enriched fluorine-containing 2-oxoindoles are of great significance in drug discovery and development [45]. Unfortunately, there was no ee observed when the 3-substituent was changed to an alkyl group (Table 3, entry 18).

**Table 3:** Substrate scope.

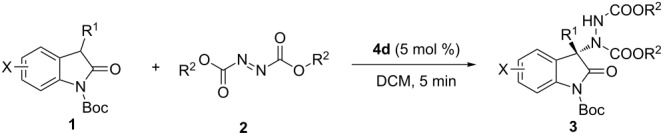					

Entry^a^	X	R^1^	R^2^	Yield(%)^b^	ee(%)^c^

1	H	Ph	*t*-Bu	87 (**3d**)	93
2	5-Me	Ph	*t*-Bu	85 (**3e**)	96
3	5-OMe	Ph	*t*-Bu	88 (**3f**)	96
4	5-Me	Ph	Et	88 (**3g**)	86
5	5-OMe	Ph	Et	84 (**3h**)	88
6	5-F	Ph	Et	84 (**3i**)	87
7	5-Cl	Ph	Et	85 (**3j**)	90
8	6-Cl	Ph	Et	87 (**3k**)	87
9	H	4-MeC_6_H_4_	*t*-Bu	89 (**3l**)	81
10	H	4-OMeC_6_H_4_	*t*-Bu	85 (**3m**)	95
11	H	4-MeC_6_H_4_	Et	90 (**3n**)	81
12	H	4-*t*-BuC_6_H_4_	Et	82 (**3o**)	87
13	H	3-OMeC_6_H_4_	Et	86 (**3p**)	87
14	H	4-FC_6_H_4_	*t*-Bu	87 (**3q**)	95
15	H	4-FC_6_H_4_	Et	89 (**3r**)	85
16	5-Me	4-FC_6_H_4_	*t*-Bu	85 (**3s**)	98
17	5-Me	4-MeC_6_H_4_	*t*-Bu	86 (**3t**)	96
18	H	Me	Et	72 **(3u)**	0

^a^0.1 mmol scale in 1.0 mL of DCM. At −78 °C when R = *t*-Bu, at −30 °C when R = Et. ^b^Isolated yield. ^c^Determined by chiral HPLC analysis.

**Scheme 2:**
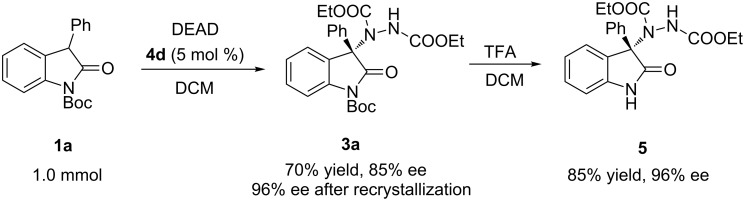
Scale-up of the reaction and deprotection of the product.

Subsequently, a scale-up experiment on 1.0 mmol scale of the reaction was examined, and the corresponding product could be obtained smoothly with a slightly reduced yield (70%) and ee (85%). The ee value of the product could be raised to 96% after a single recrystallization step (Scheme 2). The product could be deprotected to provide the known compound **5** with no deterioration in enantioselectivity. The absolute configuration of **3a** was deduced to be *S* by comparison the specific optical rotation data of **5** with literature data [10,12], and the absolute configurations of other adducts **3b**–**t** were assigned by analogy.

**Figure 1:**
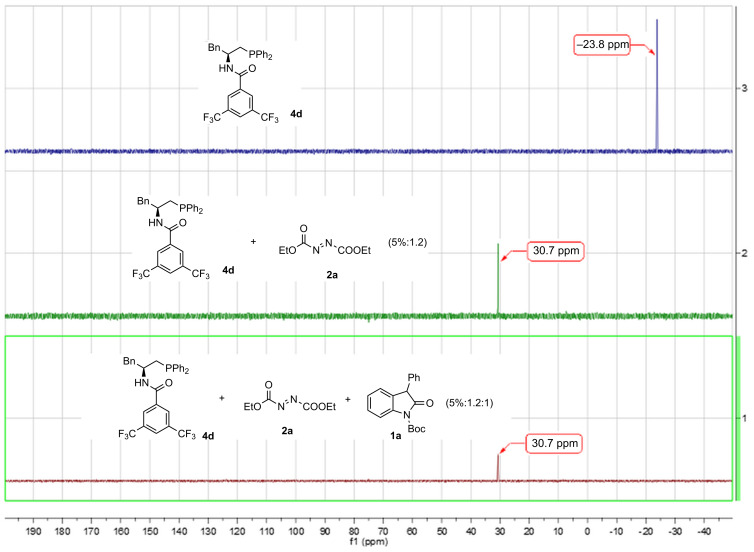
The ^31^P NMR spectra research in CD_2_Cl_2_.

**Scheme 3:**
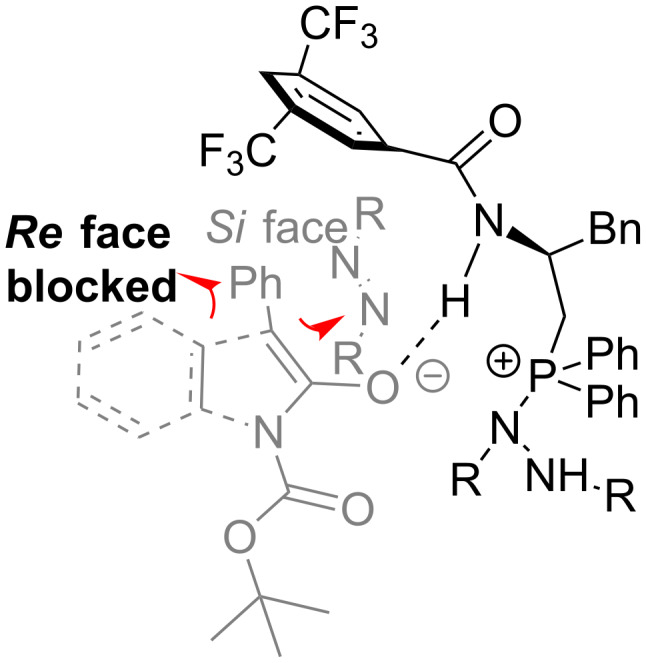
Proposed transition-state model.

To get some insight into this reaction, ^31^P NMR of the mixture of **4d** (0.5 mol %) and **2a** (0.12 mmol) in CD_2_Cl_2_ was monitored, followed by the addition of **1a** (0.1 mmol) in to the mixture (Figure 1). The formation of zwitterion intermediate **A** in Scheme 1, observed as a new ^31^P NMR chemical shift, was generated at δ = 30 ppm, and did not disappear until the reaction was finished. On the basis of the experimental results and previous related studies, a plausible transition state was proposed to explain the stereochemistry of the product (Scheme 3). We propose that after deprotonation by the basic in situ generated zwitterion, the resultant enolate form of 3-aryloxindoles might interact with the catalyst by both hydrogen bonding as well as static interaction. The presence of the 3,5-CF_3_-substituted benzene ring may block the *Re* face of the enolate, driving the electrophile to attack from the *Si* face.

## Conclusion

In summary, we have realized enantioselective α-aminations of 3-substitued oxindoles with azodicarboxylates by using amino acid-derived bifunctional phosphine catalysts. These reactions afford a variety of chiral 2-oxindoles with a tetrasubstituted carbon center attached to a nirogen atom at the C-3 position in high yields and excellent enantioselectivities. Further studies regarding the mechanism as well as the development of related reactions using this catalytic mode are currently under investigation.

## Supporting Information

File 1 Experimental part.

## References

[1] Dounay A B, Overman L E (2003). Chem Rev.

[2] Heimgartner H (1991). Angew Chem.

[3] Arend M (1999). Angew Chem.

[4] Bergmeier S C (2000). Tetrahedron.

[5] Ooi T, Takahashi M, Doda K, Maruoka K (2002). J Am Chem Soc.

[6] Suri J T, Steiner D D, Barbas C F (2005). Org Lett.

[7] Poulsen T B, Alemparte C, Jorgensen K A (2005). J Am Chem Soc.

[8] Cheng L, Liu L, Wang D, Chen Y-J (2009). Org Lett.

[9] Qian Z-Q, Zhou F, Du T-P, Wang B-L, Ding M, Zhao X-L, Zhou J (2009). Chem Commun.

[10] Zhou F, Ding M, Liu Y-L, Wang C-H, Ji C-B, Zhang Y-Y, Zhou J (2011). Adv Synth Catal.

[11] Bui T, Borregan M, Barbas C F (2009). J Org Chem.

[12] Bui T, Hernández-Torres G, Milite C, Barbas C F (2010). Org Lett.

[13] Mouri S, Chen Z, Mitsunuma H, Furutachi M, Matsunaga S, Shibasaki M (2010). J Am Chem Soc.

[14] Yang Z, Wang Z, Bai S, Shen K, Chen D, Liu X, Lin L, Feng X (2010). Chem – Eur J.

[15] Wang S-X, Han X, Zhong F, Wang Y, Lu Y (2011). Synlett.

[16] Zhao Q-Y, Lian Z, Wei Y, Shi M (2012). Chem Commun.

[17] Fan Y C, Kwon O (2013). Chem Commun.

[18] Wei Y, Shi M (2014). Chem – Asian J.

[19] Declerck V, Martinez J, Lamaty F (2009). Chem Rev.

[20] Basavaiah D, Reddy B S, Badsara S S (2010). Chem Rev.

[21] Wei Y, Shi M (2013). Chem Rev.

[22] Rauhut M, Currier H (1963). Preparation of Dialkyl 2-Methylene Glutarates. U.S. Patent.

[23] McClure J D (1970). J Org Chem.

[24] Zhao Q-Y, Pei C-K, Guan X-Y, Shi M (2011). Adv Synth Catal.

[25] Zhang X-N, Shi M (2012). Eur J Org Chem.

[26] Shi Z, Yu P, Loh T-P, Zhong G (2012). Angew Chem.

[27] Takizawa S, Nguyen T M-N, Grossmann A, Enders D, Sasai A (2012). Angew Chem.

[28] White D A, Baizer M M (1973). Tetrahedron Lett.

[29] Trost B M, Li C-J (1994). J Am Chem Soc.

[30] Trost B M, Dake G R (1997). J Org Chem.

[31] Chung Y K, Fu G C (2009). Angew Chem.

[32] Smith S W, Fu G C (2009). J Am Chem Soc.

[33] Sun J, Fu G C (2010). J Am Chem Soc.

[34] Lundgren R J, Wilsily A, Marion N, Ma C, Chung Y K, Fu G C (2013). Angew Chem.

[35] Wang T, Yao W, Zhong F, Pang G H, Lu Y (2014). Angew Chem.

[36] Mindal M, Ibrahim A A, Wheeler K A, Kerrigan N J (2010). Org Lett.

[37] Xiao H, Chai Z, Zheng C-W, Yang Y-Q, Liu W, Zhang J-K, Zhao G (2010). Angew Chem.

[38] Han X, Wang Y, Zhong F, Lu Y (2011). J Am Chem Soc.

[39] Xiao H, Chai Z, Cao D, Wang H, Chen J, Zhao G (2012). Org Biomol Chem.

[40] Chai Z, Zhao G (2012). Catal Sci Technol.

[41] Cao D, Chai Z, Zhang J, Ye Z, Xiao H, Wang H, Chen J, Wu X, Zhao G (2013). Chem Commun.

[42] Wang H-Y, Zhang K, Zheng C-W, Chai Z, Cao D-D, Zhang J-X, Zhao G (2015). Angew Chem.

[43] Mitsunobu O, Yamada Y (1967). Bull Chem Soc Jpn.

[44] Zhong F, Dou X, Han X, Yao W, Zhu Q, Meng Y, Lu Y (2013). Angew Chem.

[45] Banks R E, Smart B E, Tatlow J C (1994). Organofluorine Chemistry: Principles and Commercial Applications.

