# Supplementary material for: Asymmetric α-amination of 3-substituted oxindoles using chiral bifunctional phosphine catalysts
Source: Beilstein J Org Chem. 2016 Apr 15;12:725–31. doi: 10.3762/bjoc.12.72 (PMC4902028; doi:10.3762/bjoc.12.72)
Supplement: File 1 — Experimental part. [file Beilstein_J_Org_Chem-12-725-s001.pdf]

Supporting Information

for

Asymmetric α-amination of 3-substituted oxindoles using chiral bifunctional phosphine catalysts

Qiao-Wen Jin[1], Zhuo Chai[2], You-Ming Huang[2], Gang Zou[*,§,1], and Gang Zhao[*,¶,2]

Address: [1]Laboratory of Advanced Materials and Institute of Fine Chemicals, East China University of Science and Technology, 130 Meilong Road, Shanghai 200237, People's Republic of China and [2]Key Laboratory of Synthetic Chemistry of Natural Substances, Shanghai Institute of Organic Chemistry, Chinese Academy of Sciences, 345 Lingling Road, Shanghai 200032, People's Republic of China

Email: Gang Zou - zougang@ecust.edu.cn;

Gang Zhao - zhaog@mail.sioc.ac.cn

*Corresponding author

[§]Fax: (+86)-21-6425-3881.

[¶]Fax: (+86)-21-6416-6128

Experimental part

Contents

General information……………………………………………………….....s2
Synthesis of 3-substituted oxindoles……………………………………………s3
Asymmetric α-amination of 3-substituted oxindoles......................................s4
Transformation of product **5**……………………………………………………..s19
References………………………………………………………………………..s20
NMR and HPLC spectra of **1p**, **3a–u** and **5**……………………………........s21

General information

The ^{1}H NMR spectra were recorded on a Bruker (400 MHz) spectrometer. All chemical shifts (δ) were given in ppm. Data were reported as follows: chemical shift, intergration, multiplicity (s = single, d = doublet, t = triplet, q = quarter, br = broad, m= multiplet, cm = complex multiplet) and coupling constants (Hz). ^{13}C NMR spectra were recorded on a DPX-400 spectrometer (at 100 MHz). ^{19}F NMR were recorded on a Agilent 400 spectrometer (at 376 MHz). ^{31}P NMR were recorded on a Agilent 400 spectrometer (at 163 MHz). Flash column chromatography was performed using H silica gel. For thin-layer chromatography (TLC), silica gel plates (HSGF 254) were used and compounds were visualized by irradiation with UV light. Analytical high performance liquid chromatography (HPLC) was carried out on SHIMADZU equipment using chiral columns. Melting points were determined on a SGW X-4 melting point apparatus and were uncorrected. Optical rotations were measured on a JASCO P-1010 Polarimeter at λ = 589 nm. IR spectra were recorded on a Perkin-Elmer 983G instrument. Mass spectra analysis was performed on API 200 LC/MS system (Applied Biosystems Co. Ltd.).

Commercially available materials were purchased from Sigma-Aldrich®, Adamas-beta® or Energy Chemical® were used as received. The synthesis of 3-substituted oxindoles and the catalysts were performed according to reported methods [1-7].

Synthesis of 3-substituted oxindoles

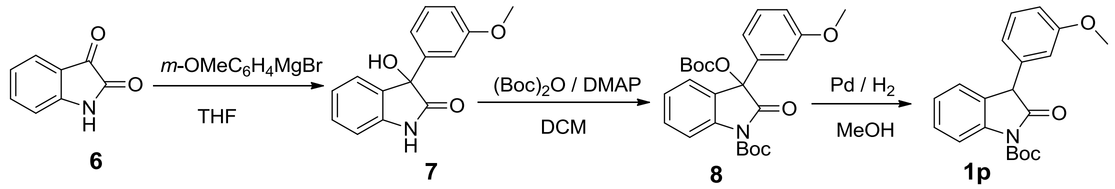

tert-Butyl 3-(3-methoxyphenyl)-2-oxoindoline-1-carboxylate (**1p**): A solution of *m*-OMeC_6H_4MgBr in THF (2.0 M, 6.8 mL) was added to a stirred cold (−40 °C) suspension of isatin **6** (1.0 g, 6.8 mmol) in THF (30 mL) under an atmosphere of N_2. The mixture was allowed to warm to room temperature and was stirred until isatin was consumed. The reaction mixture was diluted with ether, cooled in an ice-bath, and then quenched with 1.0 M HCl. The aqueous layer was extracted with ether, and the combined organic layers were washed with water and brine and then dried over Na_2SO_4. After the removal of solvent, purification by flash column chromatography (hexane/acetone = 4:1) was carried out to give **7** in 80% yield (1.24 g). **7** (67.3 mg, 0.264 mmol) was dissolved in CH_2Cl_2 (2.6 mL). To this solution were added DMAP (3.2 mg, 0.0264 mmol) and $(Boc)_2O$ (66 mg, 0.3 mmol) at room temperature, and then the mixture was stirred for 3 h. The reaction mixture was diluted with ethyl acetate, and then quenched with saturated aqueous NH_4Cl. The aqueous layer was extracted with ethyl acetate, and the combined organic layers were washed with water and brine, then dried over Na_2SO_4 and evaporated to give product **8**, which was dissolved in MeOH (5 mL). Pd/C (20 mg, 5% w/w) was added to this solution, and the resulting mixture was stirred under hydrogen atmosphere (balloon) for 3 h at room temperature. The reaction mixture was passed through celite to remove Pd/C, and the residue was washed with ether. After the removal of solvent, the crude product was purified by flash column chromatography (hexane/acetone = 12:1) to give **1p** in 80% yield (71.6 mg) for two steps as a white solid. mp 67-69 °C. ^{1}H NMR (400 MHz, $CDCl_3$): δ(ppm) = 7.92 (d, J = 8.0 Hz, 1H), 7.33-7.37 (m, 1H), 7.24 (t, J = 8.0 Hz, 1H), 7.15-7.16

(br, 2H), 6.82-6.85 (m, 1H), 6.78 (d, *J* = 7.6 Hz, 1H), 6.72-6.73 (m, 1H), 4.68 (s, 1H), 3.76 (s, 3H), 1.63 (s, 9H). ^{13}C NMR (100 MHz, $CDCl_3$) δ(ppm) = 173.7, 160.0, 149.4, 140.5, 137.7, 129.9, 128.7, 127.4, 125.1, 124.6, 121.0, 115.1, 114.6, 113.2, 84.4, 55.3, 52.5, 28.1. IR (KBr): 2979, 2922, 1794, 1768, 1729, 1600, 1584, 1490, 1480, 1369, 1345, 1288, 1251, 1147, 1088, 1048, 754. HRMS (ESI) calcd for $C_{20}H_{21}N_1O_4$ $(M+Na)^+$ 362.1368, found 362.1450.

Asymmetric α-amination of 3-substituted oxindoles

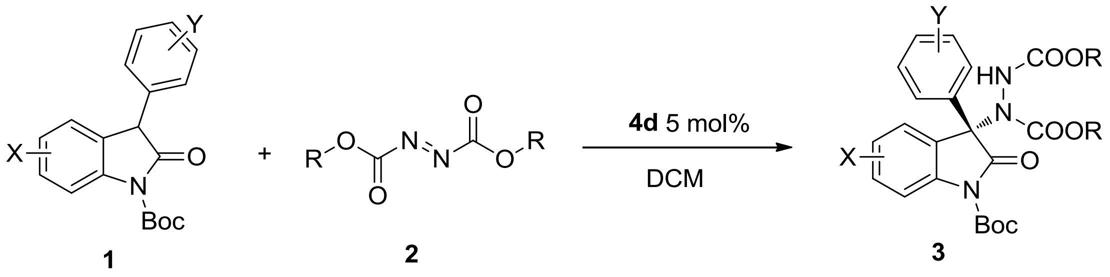

To a solution of the corresponding catalyst **4** (5 mol %) in solvent (1.0 mL) was added 3-substituted oxindole **1**, and cooled to −78 °C before the di-*tert*-butyl azodicarboxylate was introduced (to −30 °C when diethyl azodicarboxylate was used). The solvent was removed after the reaction was finished (determined by TLC analysis), the residue was purified directly by column chromatography on silica gel using hexane/ethyl acetate (4:1) as an eluent to afford **3**.

(*S*)-Diethyl 1-(1-(*tert*-butoxycarbonyl)-2-oxo-3-phenylindolin-3-yl)hydrazine-1,2-dicarboxylate (**3a**).

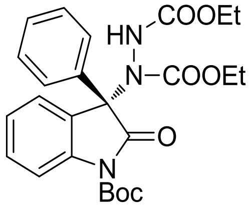

White solid, m.p. 140-143°C. 87% yield**.** ^{1}H NMR (400 MHz,

$CDCl_3$) δ(ppm) = 8.16 (d, *J* = 7.2 Hz, 1H), 7.83 (d, *J* = 8.0 Hz, 1H), 7.55 (br, 2H), 7.29-7.39 (m, 5H), 6.54 (s, 1H), 4.00-4.08 (m, 4H), 1.60 (s, 9H), 1.10 (t, *J* = 7.2 Hz, 3H), 1.06 (t, *J* = 7.2 Hz, 3H). ^{13}C NMR (100 MHz, $CDCl_3$) δ(ppm) = 173.7, 155.8, 154.4, 148.9, 139.0, 132.4, 129.7, 129.1, 128.4, 128.2, 128.0, 126.5, 124.4, 114.9, 84.4, 72.5, 63.1, 62.0, 28.1, 14.3, 13.9. IR (KBr): 3315, 2981, 2934, 1731, 1479, 1466, 1373, 1343, 1290, 1248, 1151, 1093, 1064, 758, 728, 404. HRMS (ESI) calcd for $C_{25}H_{29}N_3O_7$ $(M+Na)^+$ 506.1903, found 506.1895. $[\alpha]^{23.4}_D$ = +74.8 (*c* 1.7, $CHCl_3$) HPLC (Daicel Chiralpak AD-H, *i*-PrOH / Hexane=10 : 90, 220 nm, 1.0 mL/min): major 10.7 min, minor 20.2 min. Enantiomeric excess: 90%.

(*S*)-Diisopropyl1-(1-(*tert*-butoxycarbonyl)-2-oxo-3-phenylindolin-3-yl)-hydrazine-1,2-dicarboxylate (**3b**).

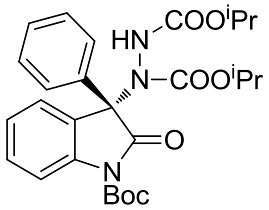

Colorless oil. 93% yield. ^{1}H NMR (400 MHz, $CDCl_3$) δ(ppm) = 8.20 (d, *J* = 7.2 Hz, 1H), 7.83 (d, *J* = 8.0 Hz, 1H), 7.55 (br, 2H), 7.36 (t, *J* = 7.6 Hz, 1H), 7.27-7.31 (m, 4H), 6.43 (s, 1H), 4.76-4.81 (m, 2H), 1.60 (s, 9H), 1.18 (d, *J* = 6.4 Hz, 3H), 1.08 (d, *J* = 6.4 Hz, 3H), 0.99 (d, *J* = 6.4 Hz, 3H), 0.88 (d, *J* = 6.0 Hz, 3H). ^{13}C NMR (100 MHz, $CDCl_3$) δ(ppm) = 173.8, 155.5, 153.9, 149.1, 139.1, 132.6, 129.7, 129.1, 129.0, 128.4, 126.6, 124.4, 114.8, 84.3, 72.5, 71.2, 69.8, 28.1, 21.8, 21.7, 21.5, 21.4. IR (KBr): 3322, 2981, 2934, 1778, 1731, 1479, 1466, 1373, 1251, 1151, 1108, 758. HRMS (ESI) calcd for

$C_{27}H_{33}N_3O_7$ (M+Na)$^+$ 534.2216, found 534.2205. $[\alpha]^{28.5}_D$ = +52.5 (*c* 1.5, $CHCl_3$) HPLC (Daicel Chiralpak AD-H, *i*-PrOH / Hexane=10 : 90, 220 nm, 1.0 mL/min): major 9.8 min, minor 27.2 min. Enantiomeric excess: 89%.

(*S*)-Di-*tert*-butyl1-(1-(*tert*-butoxycarbonyl)-2-oxo-3-phenylindolin-3-yl)hydrazine-1,2-dicarboxylate (**3d**).

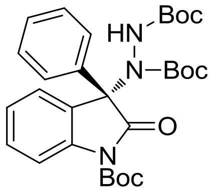

White solid, m.p. 82-85°C. 87% yield. ^1H NMR (400 MHz, $CDCl_3$) δ(ppm) = 8.25 (d, *J* = 7.2 Hz, 1H), 7.80 (d, *J* = 8.0 Hz, 1H), 7.54 (br, 2H), 7.32-7.36 (m, 1H), 7.26-7.31 (m, 4H), 6.30 (s, 1H), 1.60 (s, 9H), 1.30 (s, 9H), 1.20(s, 9H). ^{13}C NMR (100 MHz, $CDCl_3$) δ(ppm) = 174.1, 154.8, 153.1, 149.1, 138.7, 132.9, 129.7, 129.3, 128.8, 128.7, 128.2, 126.4, 124.4, 114.8, 84.2, 83.1, 80.9, 72.5, 28.1, 28.0, 27.7. IR (KBr): 3336, 2979, 2932, 1778, 1729, 1479, 1368, 1345, 1249, 1152, 758. HRMS (ESI) calcd for $C_{29}H_{37}N_3O_7$ (M+Na)$^+$ 562.2529, found 562.2511. $[\alpha]^{28.6}_D$ = +26.4 (*c* 1.7, $CHCl_3$) HPLC (Daicel Chiralpak AD-H / AD, *i*-PrOH / Hexane=5 : 95, 220 nm, 0.7 mL/min): major 66.9 min, minor 80.7 min. Enantiomeric excess: 93%.

(*S*)-Di-*t*ert-butyl 1-(1-(*t*ert-butoxycarbonyl)-5-methyl-2-oxo-3-phenylindolin-3-yl)hydrazine-1,2-dicarboxylate (**3e**)

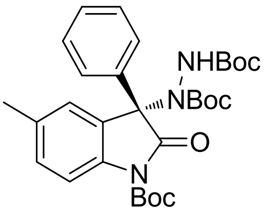
White solid, m.p. 71-73°C. 85% yield. ^{1}H NMR (400 MHz, $CDCl_3$) δ(ppm) = 7.98 (s, 1H), 7.66 (d, *J* = 8.4 Hz, 1H), 7.55 (br, 2H), 7.27-7.30 (m, 3H), 7.14 (d, *J* = 8.4 Hz, 1H), 6.30 (s, 1H), 2.44 (s, 3H), 1.60 (s, 9H), 1.30 (s, 9H), 1.20 (m, 9H). ^{13}C NMR (100 MHz, $CDCl_3$) δ(ppm) = 174.4, 154.7, 153.3, 149.1, 136.3, 133.3, 134.0, 133.2, 129.6, 129.3, 128.7, 128.2, 126.6, 115.0, 114.6, 84.1, 83.1, 80.8, 72.7, 28.1, 28.0, 27.7, 21.4. IR (KBr): 3322, 2979, 2931, 1778, 1728, 1490, 1393, 1368, 1337, 1304, 1278, 1247, 1154, 732. HRMS (ESI) calcd for $C_{30}H_{39}N_3O_8$ $(M+H)^+$ 554.2866, found 554.2860. $[\alpha]^{27.8}_{D}$ = +67.2 (*c* 2.0, $CHCl_3$) HPLC (Daicel Chiralpak OD-H, *i*-PrOH / Hexane=1 : 99, 220 nm, 1.0 mL/min): major 8.0 min, minor 11.2 min. Enantiomeric excess: 96%.

(*S*)-Di-*tert*-butyl 1-(1-(*tert*-butoxycarbonyl)-5-methoxy-2-oxo-3-phenylindolin-3-yl)hydrazine-1,2-dicarboxylate (**3f**).

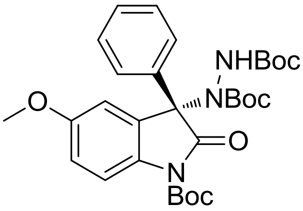
White solid, m.p. 81-83°C. 88% yield. ^{1}H NMR (400 MHz, $CDCl_3$) δ(ppm) = 7.93 (br, 1H), 7.72 (d, *J* = 8.8 Hz, 1H), 7.54 (br, 2H), 7.29-7.31 (m, 3H), 6.87-6.90 (dd, *J* = 2.8 Hz, 2.8Hz, 1H), 6.28 (s, 1H), 3.88 (s, 3H), 1.59 (s, 9H), 1.29 (s, 9H), 1.22 (s, 9H). ^{13}C NMR (100 MHz, $CDCl_3$) δ(ppm) = 174.0,

156.9, 154.7, 153.2, 149.2, 133.3, 132.3, 130.5, 129.6, 128.8, 128.3, 115.7, 114.1, 112.6, 84.0, 83.1, 80.9, 72.8, 55.8, 28.1, 28.0, 27.8. IR (KBr): 3335, 2979, 2932, 1776, 1727, 1488, 1368, 1301, 1277, 1246, 1154, 758. HRMS (ESI) calcd for $C_{30}H_{39}N_3O_8$ $(M+H)^+$ 570.2815, found 570.2812. $[\alpha]^{21.3}_D$ = +79.9 (*c* 1.0, $CHCl_3$) HPLC (Daicel Chiralpak IC / PC-II, *i*-PrOH / Hexane=10:90, 220 nm, 1.0 mL/min): major 12.9 min, minor 22.8 min. Enantiomeric excess: 96%.

(*S*)-Diethyl1-(1-(*tert*-butoxycarbonyl)-5-methyl-2-oxo-3-phenylindolin-3-yl)-hydrazine-1,2-dicarboxylate (**3g**).

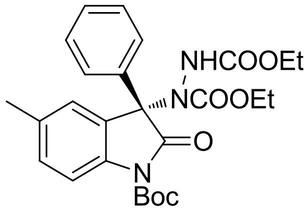

White solid, m.p. 180-182°C. 88% yield. ^{1}H NMR (400 MHz, $CDCl_3$) δ(ppm) = 7.93 (s, 1H), 7.69 (d, *J* = 8.4 Hz, 1H), 7.56-7.57 (br, 2H), 7.31-7.32 (m, 3H), 7.15 (d, *J* = 8.0 Hz, 1H), 6.51 (s, 1H), 4.02-4.07 (m, 4H), 2.44 (s, 3H), 1.60 (s, 9H), 1.05-1.12 (m, 6H). ^{13}C NMR (100 MHz, $CDCl_3$) δ(ppm) = 173.9, 155.7, 154.5, 149.0, 136.6, 134.0, 132.5, 129.7, 129.0, 128.4, 128.2, 128.1, 126.8, 114.7, 84.2, 72.7, 63.1, 61.9, 29.7, 21.4, 14.3, 13.9. IR (KBr): 3314, 2981, 2932, 1730, 1491, 1373, 1336, 1301, 1245, 1154, 1064, 729, 406. HRMS (ESI) calcd for $C_{26}H_{31}N_3O_7$ $(M+Na)^+$ 520.2060, found 520.2052. $[\alpha]^{28.9}_D$ = +110.1 (*c* 1.0, $CHCl_3$) HPLC (Daicel Chiralpak AD-H, *i*-PrOH / Hexane=10 : 90, 220 nm, 1.0 mL/min): major 11.0 min, minor 25.4 min. Enantiomeric excess: 86%.

(*S*)-Diethyl1-(1-(*tert*-butoxycarbonyl)-5-methoxy-2-oxo-3-phenylindolin-3-yl)-hydrazine-1,2-dicarboxylate (**3h**).

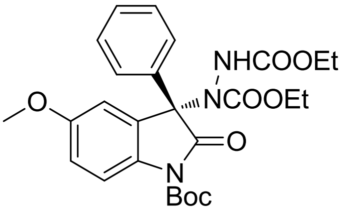

White solid, m.p. 89-91°C. 84% yield.^{1}H NMR (400 MHz, $CDCl_3$) δ(ppm) = 7.81 (br, 1H), 7.74 (d, *J* = 8.8 Hz, 1H), 7.55-7.56 (br, 2H), 7.32 (t, *J*=3.2 Hz, 3H), 6.89 (dd, *J* = 2.8 Hz, 2.4 Hz, 1H), 6.52 (s, 1H), 4.02-4.09 (m, 4H), 3.87 (s, 3H), 1.59 (s, 9H), 1.06-1.11 (m, 6H). ^{13}C NMR (100 MHz, $CDCl_3$) δ(ppm) = 173.7, 156.7, 155.7, 154.4, 149.0, 132.5, 129.6, 129.1, 129.0, 128.4, 128.2, 115.8, 114.3, 112.5, 84.1, 72.8, 63.1, 61.9, 55.7, 28.1, 14.3, 13.9. IR (KBr): 3314, 2981, 2934, 1728, 1488, 1373, 1338, 1244, 1153, 1064, 730. HRMS (ESI) calcd for $C_{26}H_{31}N_3O_8$ $(M+Na)^+$ 536.2009, found 536.1997. $[\alpha]_D$ +110.1 (*c* 1.0, $CHCl_3$) HPLC (Daicel Chiralpak AD-H, *i*-PrOH / Hexane=10:90, 220 nm, 1.0 mL/min): major 16.6 min, minor 32.9 min. Enantiomeric excess: 88%.

(*S*)-Diethyl1-(1-(*tert*-butoxycarbonyl)-5-fluoro-2-oxo-3-phenylindolin-3-yl)-hydrazine-1,2-dicarboxylate (**3i**).

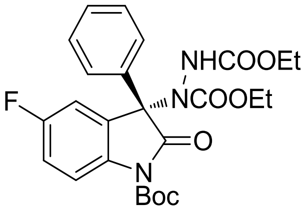

White solid, m.p. 138-140°C. 84% yield.^{1}H NMR (400

MHz, $CDCl_3$) δ(ppm) = 8.00 (dd, *J* = 2.8 Hz, 8Hz, 1H), 7.82 (q, *J* = 4.4 Hz, 1H), 7.52-7.53 (br, 2H), 7.33-7.35 (m, 3H), 7.04-7.09 (dt, *J* = 2.8 Hz, 8.8Hz, 1H), 6.52 (s, 1H), 4.01-4.10 (m, 4H), 1.60 (s, 9H), 1.10 (t, *J* = 7.2 Hz, 6H). ^{13}C NMR (100 MHz, $CDCl_3$) δ(ppm) = 173.3, 159.8 (d, *J* = 242.6 Hz), 155.8, 154.3, 148.9, 135.0 (d, *J* = 2.4 Hz), 131.8, 129.6, 129.3, 128.5, 116.2 (d, *J* = 7.8 Hz), 115.7 (d, *J* = 22.9 Hz), 114.3, 114.1, 84.5, 72.5, 63.3, 62.1, 28.0, 14.3, 14.0. ^{19}F NMR (376 MHz, $CDCl_3$) δ(ppm) = -117.1 (m, 1F). IR (KBr): 3319, 2981, 2934, 1778, 1731, 1482, 1373, 1342, 1299, 1244, 1150, 1062, 817, 728, 415. HRMS (ESI) calcd for $C_{25}H_{28}FN_3O_7$ $(M+Na)^+$ 524.1809, found 524.1797. $[\alpha]^{29.7}_D$ = +55.7 (*c* 1.6, $CHCl_3$) HPLC (Daicel Chiralpak AD-H, *i*-PrOH / Hexane=10 : 90, 220 nm, 1.0 mL/min): major 8.8 min, minor 14.3 min. Enantiomeric excess: 87%.

(*S*)-Diethyl1-(1-(*tert*-butoxycarbonyl)-5-chloro-2-oxo-3-phenylindolin-3-yl)-hydrazine-1,2-dicarboxylate (**3j**).

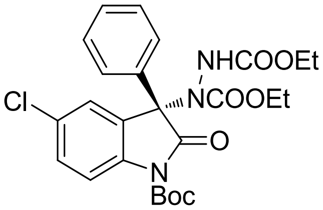

White solid, m.p. 156-158°C. 85% yield**.** ^1H NMR (400 MHz, $CDCl_3$) δ(ppm) = 8.21 (d, *J* = 2.0 Hz, 1H), 7.80 (d, *J* = 8.4 Hz, 1H), 7.52-7.53 (br, 2H), 7.33-7.35 (m, 4H), 6.49 (s, 1H), 4.01-4.10 (m, 4H), 1.59 (s, 9H), 1.10-1.12 (m, 6H). ^{13}C NMR (100 MHz, $CDCl_3$) δ(ppm) = 173.1, 155.7, 154.4, 148.8, 137.5, 131.7, 130.0, 129.9, 129.6, 129.3, 129.2, 128.5, 126.6,

116.2, 84.7, 72.3, 63.3, 62.1, 28.0, 14.3, 14.0. IR (KBr): 3323, 2982, 2932, 1781, 1732, 1472, 1373, 1334, 1296, 1248, 1152, 1104, 1063, 760. HRMS (ESI) calcd for $C_{25}H_{28}ClN_3O_7$ $(M+Na)^+$ 540.1513, found 540.1489. $[\alpha]^{23.5}_D$ = +100.5 (*c* 1.7, $CHCl_3$) HPLC (Daicel Chiralpak AD-H, *i*-PrOH / Hexane=10 : 90, 220 nm, 1.0 mL/min): major 9.4 min, minor 17.1 min. Enantiomeric excess: 90%.

(*S*)-Diethyl1-(1-(*tert*-butoxycarbonyl)-6-chloro-2-oxo-3-phenylindolin-3-yl)-hydrazine-1,2-dicarboxylate (**3k**).

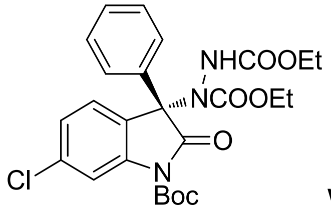

White solid, m.p. 75-77°C. 87% yield. ^{1}H NMR (400 MHz, $CDCl_3$) δ(ppm) = 8.11 (d, *J* = 8.4 Hz, 1H), 7.92 (d, *J* = 1.6 Hz, 1H), 7.50 (t, *J* = 4.0 Hz, 2H), 7.28-7.34 (m, 4H), 6.51 (s, 1H), 4.02-4.09 (m, 4H), 1.60 (s, 9H), 1.10 (t, *J* = 7.2 Hz, 6H). ^{13}C NMR (100 MHz, $CDCl_3$) δ(ppm) = 173.1, 155.8, 154.4, 148.8, 140.0, 135.0, 132.0, 129.6, 129.3, 128.4, 127.5, 126.4, 124.5, 115.7, 84.9, 72.2, 63.3, 62.1, 28.0, 14.3, 14.0. IR (KBr): 3304, 2963, 2928, 1781, 1731, 1476, 1373, 1338, 1260, 800, 406. HRMS (ESI) calcd for $C_{25}H_{28}ClN_3O_7$ $(M+Na)^+$ 540.1513, found 540.1500. $[\alpha]^{27.8}_D$ = +211.1 (*c* 0.1, $CHCl_3$) HPLC (Daicel Chiralpak AD-H, *i*-PrOH / Hexane=10 : 90, 220 nm, 1.0 mL/min): major 7.5 min, minor 10.7 min. Enantiomeric excess: 87%.

(*S*)-Di-*tert*-butyl 1-(1-(*tert*-butoxycarbonyl)-2-oxo-3-(*p*-tolyl)indolin-3-yl)-hydrazine-1,2-dicarboxylate (**3l**).

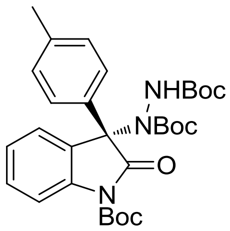

White solid, m.p. 79-81°C. 89% yield**.** ^{1}H NMR (400 MHz, $CDCl_3$) δ(ppm) = 8.23 (d, *J* = 6.8 Hz, 1H), 7.78 (d, *J* = 8.0 Hz, 1H), 7.41 (d, *J* = 8.0 Hz, 2H), 7.28-7.35 (m, 2H), 7.10 (d, *J* = 8.4 Hz, 2H), 6.29 (s, 1H), 2.30 (s, 3H), 1.60 (s, 9H), 1.30 (s, 9H) 1.19 (s, 9H). ^{13}C NMR (100 MHz, $CDCl_3$) δ(ppm) = 174.2, 154.8, 153.1, 149.1, 138.7, 135.06, 129.8, 129.6, 129.5, 128.9, 128.7, 126.3, 124.4, 114.7, 84.2, 83.0, 80.9, 72.3, 28.1, 28.0, 27.7, 21.0. IR (KBr): 3338, 2979, 2932, 1778, 1730, 1479, 1466, 1368, 1346, 1290, 1248, 1153, 758. HRMS (ESI) calcd for $C_{30}H_{39}N_3O_7$ $(M+H)^+$ 554.2866, found 554.2858. $[\alpha]^{27.9}_{D}$ = +32.5 (*c* 1.8, $CHCl_3$) HPLC (Daicel Chiralpak AD-H / AD, *i*-PrOH / Hexane=10 : 90, 220 nm, 1.0 mL/min): major 23.8 min, minor 39.2 min. Enantiomeric excess: 81%.

(*S*)-Di-*tert*-butyl 1-(1-(*tert*-butoxycarbonyl)-3-(4-methoxyphenyl)-2-oxoindolin-3-yl)hydrazine-1,2-dicarboxylate (**3m**).

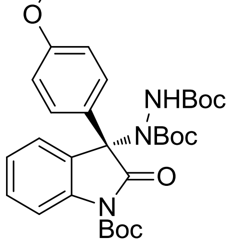

White solid, m.p. 82-85°C. 85% yield**.** ^{1}H NMR (400 MHz, $CDCl_3$) δ(ppm) = 8.24 (d, *J* = 6.8 Hz, 1H), 7.79 (d, *J* = 8.0 Hz, 1H), 7.46 (d, *J* =

8.4 Hz, 2H), 7.27-7.36 (m, 2H), 6.81 (d, *J* = 8.8 Hz, 2H), 6.31 (s, 1H), 3.77 (s, 3H), 1.60 (s, 9H), 1.31 (s, 9H), 1.19 (s, 9H). ^{13}C NMR (100 MHz, $CDCl_3$) δ(ppm) = 174.3, 159.8, 154.8, 153.0, 149.1, 138.6, 131.1, 129.6, 128.7, 126.3, 124.6, 124.4, 114.7, 113.5, 84.2, 83.0, 80.9, 72.0, 55.3, 28.1, 28.0, 27.7. IR (KBr): 3339, 2979, 2933, 1777, 1731, 1609, 1511, 1479, 1393, 1368, 1298, 1251, 1153, 1058, 833, 757. HRMS (ESI) calcd for $C_{26}H_{31}N_3O_7$ $(M+H)^+$ 570.2815, found 570.2810. $[\alpha]^{26.4}_D$ = +38.7 (*c* 2.1, $CHCl_3$) HPLC (Daicel Chiralpak AD-H / AD, *i*-PrOH / Hexane=10 : 90, 220 nm, 1.0 mL/min): major 33.3 min, minor 49.0 min. Enantiomeric excess: 95%.

(*S*)-Diethyl 1-(1-(*tert*-butoxycarbonyl)-2-oxo-3-(*p*-tolyl)indolin-3-yl)hydrazine-1,2-dicarboxylate (**3n**).

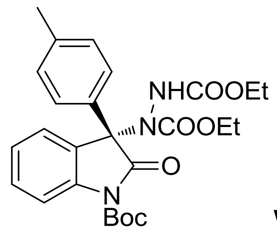

White solid, m.p. 88-90°C. 90% yield**.** ^{1}H NMR (400 MHz, $CDCl_3$) δ(ppm) = 8.14 (d, *J* = 7.2 Hz, 1H), 7.82 (d, *J* = 8.0 Hz, 1H), 7.42 (d, *J* = 8.0 Hz, 2H), 7.35 (t, *J* = 7.2 Hz, 1H), 7.27-7.32 (m, 1H), 7.12 (d, *J* = 8.0 Hz, 2H), 6.54 (s, 1H), 4.00-4.09 (m, 4H), 2.31 (s, 3H), 1.60 (s, 9H), 1.11 (t, *J* = 7.2 Hz, 3H), 1.05 (t, *J* = 7.2 Hz, 3H). ^{13}C NMR (100 MHz, $CDCl_3$) δ(ppm) = 173.8, 155.8, 154.4, 149.0, 139.1, 138.9, 129.6, 129.4, 129.1, 129.0, 128.2, 126.4, 124.4, 114.8, 84.3, 72.4, 63.1, 61.9, 28.1, 21.0, 14.3, 13.9. IR (KBr): 3316, 2981, 2934, 1731, 1606, 1511, 1479, 1466, 1372, 1344, 1289, 1253, 1151,

1064, 834, 758, 405. HRMS (ESI) calcd for $C_{26}H_{31}N_3O_7$ $(M+Na)^+$ 520.2060, found 520.0441. $[\alpha]^{28.7}_D$ = +46.4 (*c* 0.8, $CHCl_3$) HPLC (Daicel Chiralpak AD-H, *i*-PrOH / Hexane=10 : 90, 220 nm, 1.0 mL/min): major 14.9 min, minor 55.3 min. Enantiomeric excess: 81%.

(*S*)-Diethyl 1-(1-(*tert*-butoxycarbonyl)-3-(4-(*tert*-butyl)phenyl)-2-oxoindolin-3-yl)hydrazine-1,2-dicarboxylate (**3o**).

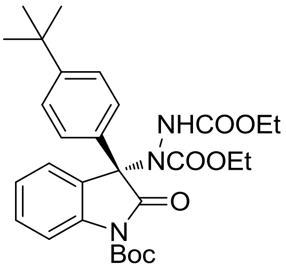

White solid, m.p. 87-88°C. 82% yield. ^{1}H NMR (400 MHz, $CDCl_3$) δ(ppm) = 8.16 (d, *J* = 7.2 Hz, 1H), 7.81 (d, *J* = 8.0 Hz, 1H), 7.47 (d, *J* = 8.4 Hz, 2H), 7.32-7.35 (m, 3H), 7.28-7.29 (br, 1H), 6.48 (s, 1H), 4.00-4.04 (m, 4H), 1.60 (s, 9H), 1.27 (s, 9H), 1.04-1.08 (m, 6H). ^{13}C NMR (100 MHz, $CDCl_3$) δ(ppm) = 173.8, 155.7, 154.5, 152.2, 149.1, 139.0, 129.5, 129.2, 129.1, 128.4, 126.4, 125.2, 124.4, 114.9, 84.3, 72.4, 63.0, 61.8, 34.6, 31.2, 28.1, 14.4, 13.9. IR (KBr): 3316, 2963, 2929, 1731, 1479, 1466, 1371, 1344, 1290, 1249, 1152, 1091, 1018, 758. HRMS (ESI) calcd for $C_{29}H_{37}N_3O_7$ $(M+Na)^+$ 562.2529, found 562.2506. $[\alpha]^{29.6}_D$ = +4.6 (*c* 0.3, $CHCl_3$) HPLC (Daicel Chiralpak AD-H, *i*-PrOH / Hexane=10 : 90, 220 nm, 1.0 mL/min): major 6.1 min, minor 8.8 min. Enantiomeric excess: 87%.

(*S*)-Diethyl 1-(1-(*tert*-butoxycarbonyl)-3-(3-methoxyphenyl)-2-oxoindolin-3-yl)-hydrazine-1,2-dicarboxylate (**3p**).

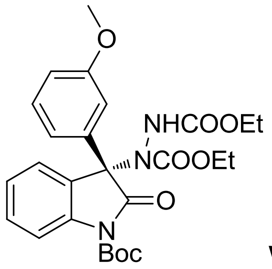

White solid, m.p. 82-84°C. 86% yield. ^{1}H NMR (400 MHz, $CDCl_3$) δ(ppm) = 8.13 (d, *J* = 7.2 Hz, 1H), 7.82 (d, *J* = 8.0 Hz, 1H), 7.36 (t, *J* = 7.6 Hz, 1H), 7.21-7.39 (m, 2H), 7.12-7.14 (br, 2H), 6.85-6.87 (br, 1H), 6.61 (s, 1H), 4.02-4.10 (m, 4H), 3.75 (s, 3H), 1.60 (s, 9H), 1.12 (t, *J* = 7.2 Hz, 3H), 1.06 (t, *J* = 7.2 Hz, 3H). ^{13}C NMR (100 MHz, $CDCl_3$) δ(ppm) = 173.5, 159.5, 154.4, 149.0, 139.1, 134.1, 129.2, 126.4, 122.0, 115.5, 114.9, 114.7, 84.4, 77.3, 76.8, 72.5, 63.1, 62.0, 55.3, 28.1, 14.3, 13.9. IR (KBr): 3311, 2981, 2940, 1731, 1601, 1466, 1343, 1251, 1151, 757. HRMS (ESI) calcd for $C_{26}H_{31}N_3O_8$ $(M+Na)^+$ 536.2009, found 536.2004. $[\alpha]^{23.4}_{D}$ +64.5 (*c* 2.1, $CHCl_3$) HPLC (Daicel Chiralpak AD-H, *i*-PrOH / Hexane=10 : 90, 220 nm, 1.0 mL/min): major 13.6 min, minor 21.2 min. Enantiomeric excess: 87%.

(*S*)-Di-*tert*-butyl 1-(1-(*tert*-butoxycarbonyl)-3-(4-fluorophenyl)-2-oxoindolin-3-yl)hydrazine-1,2-dicarboxylate (**3q**).

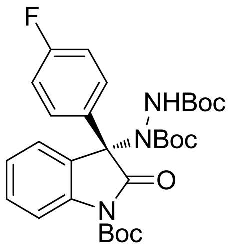

White solid, m.p. 83-85°C. 87% yield. ^{1}H NMR (400 MHz,

$CDCl_3$) δ(ppm) = 8.24 (d, *J* = 7.2 Hz, 1H), 7.80 (d, *J* = 8.0 Hz, 1H), 7.52-7.56 (m, 2H), 7.37 (t, *J* = 7.2 Hz, 1H), 7.30 (t, *J* = 7.2 Hz, 1H), 6.98 (t, *J* = 8.8 Hz, 2H), 6.33 (s, 1H), 1.61(s, 9H), 1.32 (s, 9H), 1.19 (s, 9H). ^{13}C NMR (100 MHz, $CDCl_3$) δ(ppm) = 174.1, 162.8 (d, *J* = 248.2 Hz), 154.8, 153.0, 149.0, 138.7, 131.8 (d, *J* = 8.3 Hz), 129.1, 129.0, 128.7 (d, *J* = 2.8 Hz), 126.3, 124.6, 115.1 (d, *J* = 24.5 Hz), 114.8, 84.4, 83.2, 81.1, 71.8, 28.1, 28.0, 27.7. ^{19}F NMR (376 MHz, $CDCl_3$) δ(ppm) = -112.7 (m, 1F). IR (KBr): 3337, 2980, 2933, 1777, 1731, 1605, 1508, 1479, 1369, 1345, 1289, 1153, 1091, 870, 758. HRMS (ESI) calcd for $C_{29}H_{36}FN_3O_7$ $(M+H)^+$ 558.2616, found 558.2611. $[\alpha]^{27.6}_D$ = +64.7 (*c* 1.5, $CHCl_3$) HPLC (Daicel Chiralpak AD-H / AD, *i*-PrOH / Hexane=10 : 90, 220 nm, 1.0 mL/min): major 23.1 min, minor 40.4 min. Enantiomeric excess: 95%.

(*S*)-Diethyl 1-(1-(*tert*-butoxycarbonyl)-3-(4-fluorophenyl)-2-oxoindolin-3-yl)-hydrazine-1,2-dicarboxylate (**3r**).

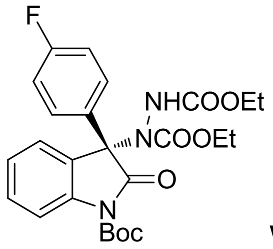

White solid, m.p. 134-136°C. 89% yield. ^{1}H NMR (400 MHz, $CDCl_3$) δ(ppm) = 8.16 (d, *J* = 7.2 Hz, 1H), 7.84 (d, *J* = 8.0 Hz, 1H), 7.53-7.57 (br, 2H), 7.38 (t, *J* = 7.6 Hz, 1H), 7.29-7.31 (br, 1H), 7.00 (t, *J* = 8.4 Hz, 2H), 6.61 (s, 1H), 4.01-4.08 (m, 4H), 1.60 (s, 9H), 1.13 (t, *J* = 7.2 Hz, 3H), 1.05 (t, *J*=7.2 Hz, 3H). ^{13}C NMR (100 MHz, $CDCl_3$) δ(ppm) = 173.7, 163.0 (d, *J* = 248.6), 154.3, 148.9, 139.0, 131.8 (d, *J* = 8.4 Hz), 129.3, 128.2 (d, *J* = 3.3 Hz),

127.9, 126.4, 124.6, 115.3 (d, J = 21.5 Hz), 115.0, 84.5, 71.9, 63.2, 62.1, 28.0, 14.4, 13.9. ^{19}F NMR (376 MHz, $CDCl_3$) δ(ppm) = -112.2 (m, 1F). IR (KBr): 3314, 2982, 2933, 1731, 1605, 1509, 1479, 1467, 1372, 1343, 1248, 1151, 1064, 837, 759, 520. HRMS (ESI) calcd for $C_{25}H_{38}FN_3O_7$ $(M+Na)^+$ 524.1809, found 524.1795. $[\alpha]^{28.4}_D$ = +57.0 (c 1.4, $CHCl_3$) HPLC (Daicel Chiralpak AD-H, *i*-PrOH / Hexane=10 : 90, 220 nm, 1.0 mL/min): major 10.1 min, minor 49.7 min. Enantiomeric excess: 85%.

(*S*)-Di-*tert*-butyl1-(1-(*tert*-butoxycarbonyl)-3-(4-fluorophenyl)-5-methyl-2-oxo-indolin-3-yl)hydrazine-1,2-dicarboxylate (**3s**).

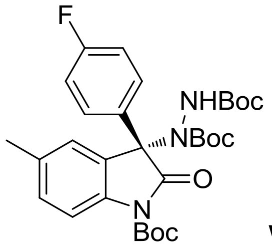

White solid, m.p. 87-89°C. 85% yield**.** ^{1}H NMR (400 MHz, $CDCl_3$) δ(ppm) = 7.98 (s, 1H), 7.67 (d, J = 8.0 Hz, 1H), 7.54 (br, 2H), 7.15 (d, J = 8.0 Hz, 1H), 6.98 (t, J = 8.0 Hz, 2H), 6.32 (s, 1H), 2.45 (s, 3H), 1.60 (s, 9H), 1.32 (s, 9H), 1.20 (s, 9H). ^{13}C NMR (100 MHz, $CDCl_3$) δ(ppm) = 174.3, 162.8 (d, J = 248.2 Hz), 154.7, 153.1, 149.0, 136.3, 134.2, 131.7 (d, J = 8.4Hz), 129.5, 129.2, 129.0 (d, J = 3.4 Hz), 126.5, 115.0 (d, J = 21.3 Hz), 114.7, 84.2, 83.2, 81.0, 72.0, 28.1, 28.0, 27.7, 21.3. ^{19}F NMR (376 MHz, $CDCl_3$) δ(ppm) = -113.0 (m, 1F). IR (KBr): 3335, 2981, 2934, 1731, 1508, 1491, 1369, 1336, 1246, 1154, 1101, 839, 758, 412. HRMS (ESI) calcd for $C_{30}H_{38}FN_3O_7$ $(M+H)^+$ 572.2772, found 572.2767. $[\alpha]^{21.3}_D$ = +76.3 (c 2.2, $CHCl_3$) HPLC (Daicel

Chiralpak AD-H, *i*-PrOH/Hexane=10 : 90, 220 nm, 1.0 mL/min): major 16.5 min, minor 26.8 min. Enantiomeric excess: 98%.

(*S*)-Di-*tert*-butyl 1-(1-(*tert*-butoxycarbonyl)-5-methyl-2-oxo-3-(*p*-tolyl)indolin-3-yl)hydrazine-1,2-dicarboxylate (**3t**)

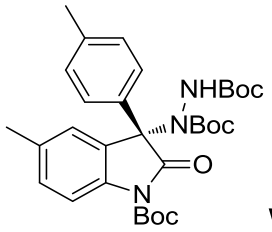

White solid, m.p. 66-69°C. 86% yield. ^{1}H NMR (400 MHz, $CDCl_3$) δ(ppm) = 7.96 (s, 1H), 7.64 (d, *J* = 8.0 Hz, 1H), 7.42 (d, *J* = 8.0 Hz, 2H), 7.09-7.14 (m, 3H), 6.29 (s, 1H), 2.44 (s, 3H), 2.30 (s, 3H), 1.59 (s, 9H), 1.30 (s, 9H), 1.19 (s, 9H). ^{13}C NMR (100 MHz, $CDCl_3$) δ(ppm) = 174.5, 154.7, 153.3, 149.1, 138.6, 136.3, 134.0, 130.2, 129.5, 129.2, 128.9, 126.5, 114.9, 114.6, 84.0, 83.0, 80.7, 72.5, 28.1, 28.0, 27.7, 21.4, 21.0. IR (KBr): 3322, 2979, 2930, 1778, 1729, 1511, 1491, 1368, 1337, 1246, 1154, 1058, 755. HRMS (ESI) calcd for $C_{31}H_{41}N_3O_7$ $(M+H)^+$ 568.3023, found 568.3017. $[\alpha]^{22.6}_{D}$ = +65.3 (*c* 2.5, $CHCl_3$) HPLC (Daicel Chiralpak AD-H, *i*-PrOH/Hexane=10 : 90, 220 nm, 1.0 mL/min): major 14.5 min, minor 24.7 min. Enantiomeric excess: 96%.

(S)-Diethyl 1-(1-(*tert*-butoxycarbonyl)-3-methyl-2-oxoindolin-3-yl)hydrazine-1,2-dicarboxylate (**3u**)

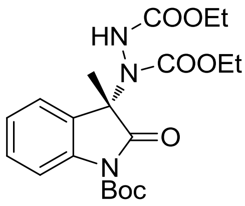

White solid, 72% yield. ^{1}H NMR (400 MHz, CDCl3): δ(ppm) = 7.82 (d, *J* = 8.4 Hz, 2H), 7.30 (t, *J* = 7.6 Hz, 1H), 7.18 (t, *J* = 7.6 Hz, 1H), 6.94 (s, 1H), 4.27-4.31 (m, 2H), 3.95-3.99 (m, 2H), 1.65 (s, 9H), 1.55 (s, 3H), 1.34 (t, *J* = 7.2 Hz, 3H), 1.00 (br, 3H). HRMS (ESI) calcd for $C_{20}H_{27}N_3O_7$ $(M+H)^+$ 422.1922, found 422.1925. $[\alpha]^{22.6}_D$ = 0 (*c* 2.5, $CHCl_3$) HPLC (Daicel Chiralpak AD-H, *i*-PrOH/Hexane=10 : 90, 220 nm, 1.0 mL/min): major 6.4 min, minor 10.0 min. Enantiomeric excess: 0%.

Transformation of product 5

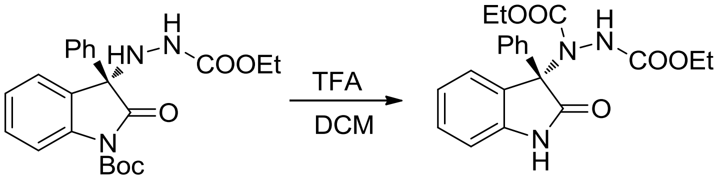

3a **5**

(S)-Diethyl 1-(2-oxo-3-phenylindolin-3-yl)hydrazine-1,2-dicarboxylate (**5**). To a solution of **3a** (48.3 mg, 0.1 mmol) in CH_2Cl_2 (5 mL) was added CF_3CO_2H (0.7 mL, 10 mmol) at 0 °C. Reaction mixture was allowed to warm up to temperature and stirred for 2 h. Saturated Na_2CO_3 aqueous solution (10 mL) was added to quench the reaction, and the resulting mixture was extracted with CH_2Cl_2 (10 mL × 3) and the combined organic layer was washed by brine (20 mL). The combined organic extracts were dried over Na_2SO_4. After the removal of solvent, the crude product was purified by flash column

chromatography (hexane/acetone = 1:1) to give **5** in 85% yield.^{1}H NMR (400 MHz, $CDCl_3$) δ(ppm) = 8.14 (s, 1H), 8.06 (d, *J* = 7.6 Hz, 1H), 7.62(br, 2H), 7.30 (t, *J* = 3.2 Hz, 3H), 7.23 (d, *J* = 7.6 Hz, 1H), 7.14 (t, *J* = 7.2 Hz, 3H), 6.81 (d, *J* = 7.6 Hz, 1H), 6.74 (s, 1H), 3.97-4.08 (m, 4H), 1.10 (t, *J* = 7.2 Hz, 3H), 1.03 (t, *J* = 7.2 Hz, 3H). ^{13}C NMR (100 MHz, $CDCl_3$) δ(ppm) = 177.5, 155.9, 154.7, 140.4, 133.1, 129.5, 129.0, 128.9, 128.3, 128.2, 127.1, 122.6, 110.0, 72.7, 63.0, 61.9, 14.3, 14.0. IR (KBr): 3288, 2982, 2931, 1727, 1620, 1472, 1406, 1377, 1341, 1243, 1097, 1061, 1021, 758, 699. HRMS (ESI) calcd for $C_{20}H_{21}N_3O_5$ $(M+Na)^+$ 406.1379, found 506.1895. $[\alpha]^{29.6}_{D}$ = +102.3 (*c* 0.5, $CHCl_3$) HPLC (Daicel Chiralpak IA, *i*-PrOH/Hexane=30:70, 214 nm, 0.7 mL/min): major 14.2 min, minor 10.9 min. Enantiomeric excess: 96%.

References

1. Xiao, H.; Chai, Z.; Zheng, C-W., Yang, Y.-Q., Zhang, J.-K. & Zhao, G. Asymmetric [3+2] cycloadditions of allenoates and dual activated olefins catalyzed by simple bifunctional *N*-acyl aminophosphines. *Angew. Chem. Int. Ed.* **2010**, *49*, 4467-4470.
2. Cao D. D., Chai Z., Zhang J. X., Ye Z. Q., Xiao H., Wang H. Y., Chen J. H., Wu X. Y., Zhao G. Thiourea-phosphonium salts from amino acids: coopereative phase-transfer catalysts in the enantioselective aza-Henry reaction. *Chem. Commun*. **2013**, *49*, 5972-5974.
3. Wang, H.-Y, Zhang, K., Zheng, C.-W., Chai, Z., Cao, D.-D., Zhang, J.-X., &

Zhao, G. Asymmetric Dual-Reagent Catalysis: Mannich-type Reactions Catalyzed by Ion Pair *Angew. Chem. Int. Ed*. **2015**, *54*, 1775-1779.

4. Ishimaru, T.; Shibata, N.; Norikawa, T.; Yasuda, N.; Nakamura, S.; Tour, T.; Shiro, M. *Angew. Chem. Int. Ed*. **2008**, *47*, 4157-4161.
5. Hamashia, Y.; Suzuki, T.; Takano, H.; Shimura, Y.; Sodeoka, M. *J. Am. Chem. Soc*. **2005**, *127*, 10164-10165.
6. Bui, T.; Gloria H. T.; Milite C.; Barbas, C. F. III. *Org. Lett*. **2010**, *12*, 5696-5699.
7. Zhong, F.; Dou, X.; Han, X.; Yao, W.; Zhu, Q.; Meng, Y.; Lu, Y. *Angew. Chem. Int. Ed.* **2013**, *52*, 943-947

The NMR and HPLC spectra of 1p, 3a–u and 5.

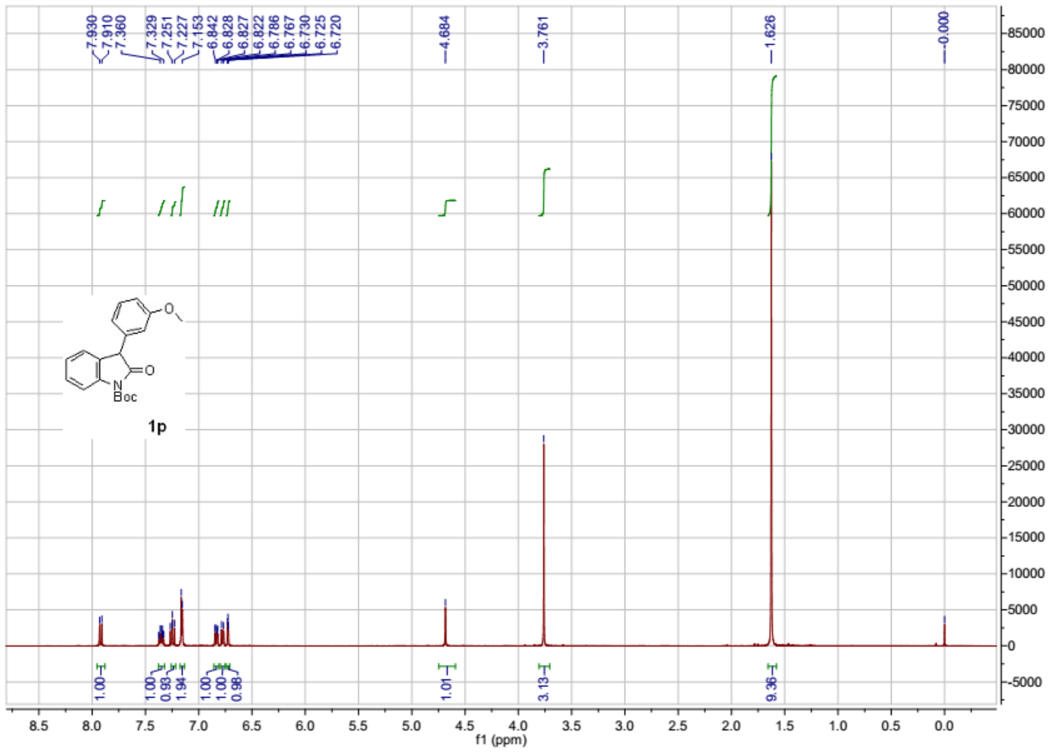

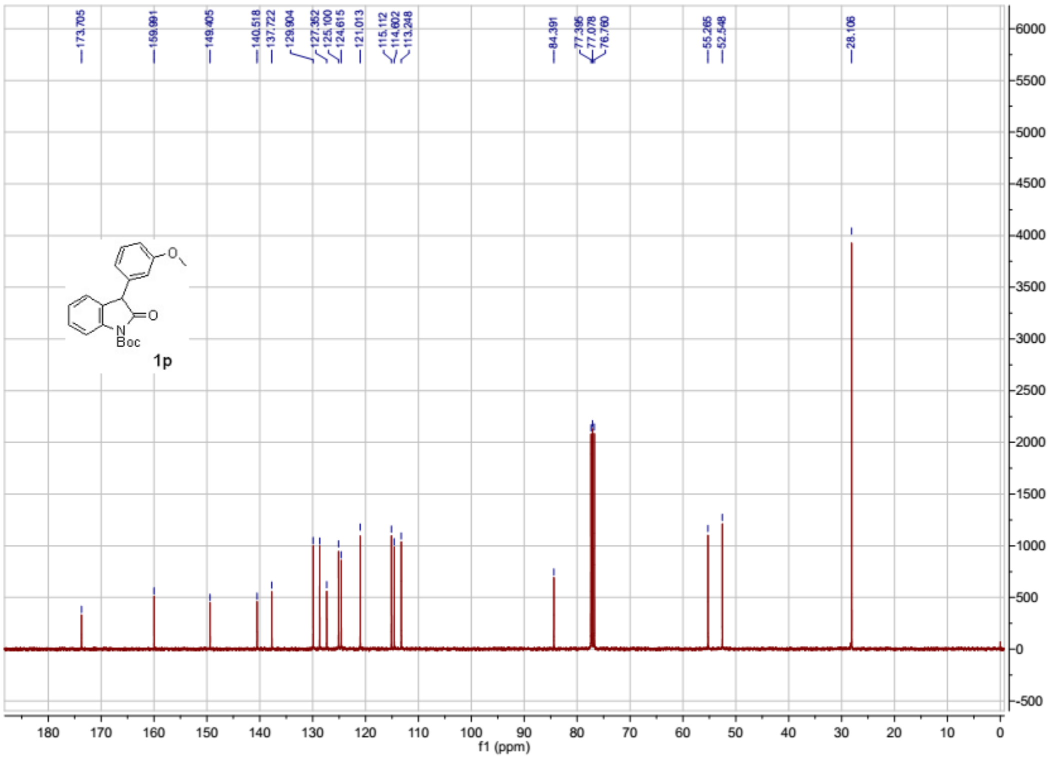
Boc
1p
f1 (ppm)

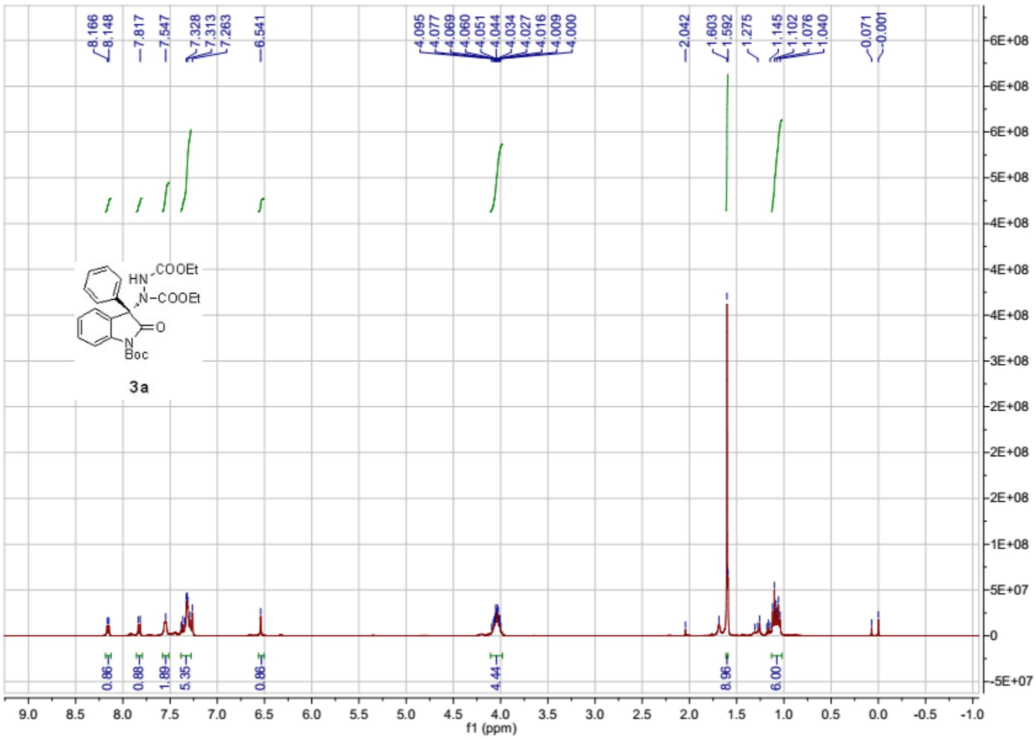
HN
COOEt
N−COOEt
Boc
3a
f1 (ppm)

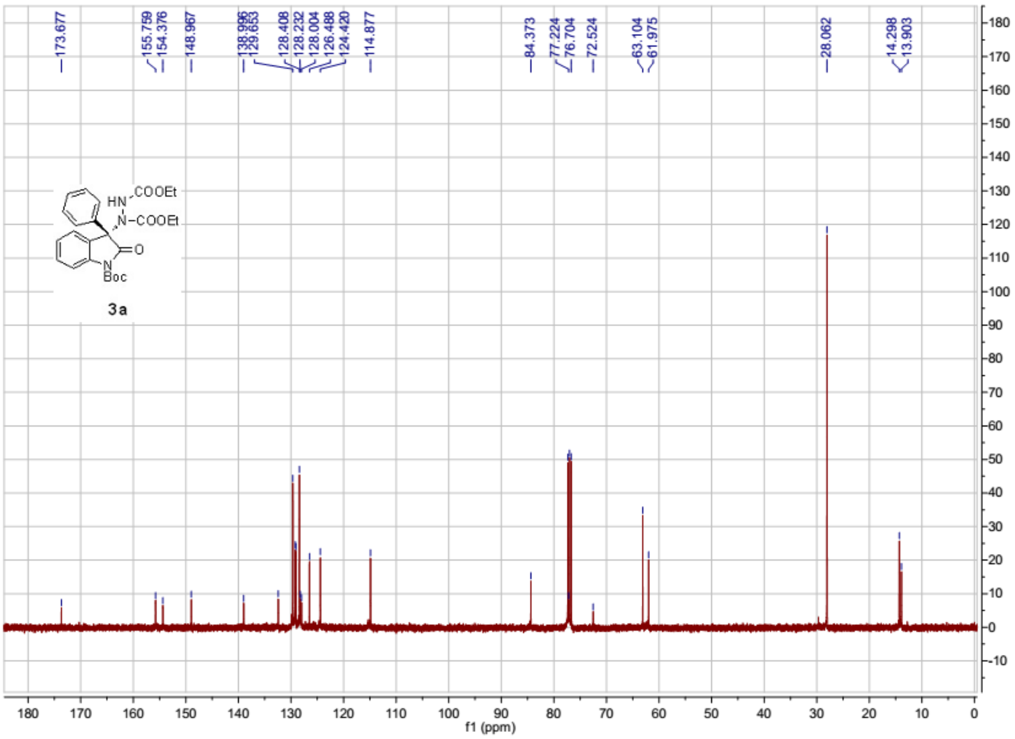

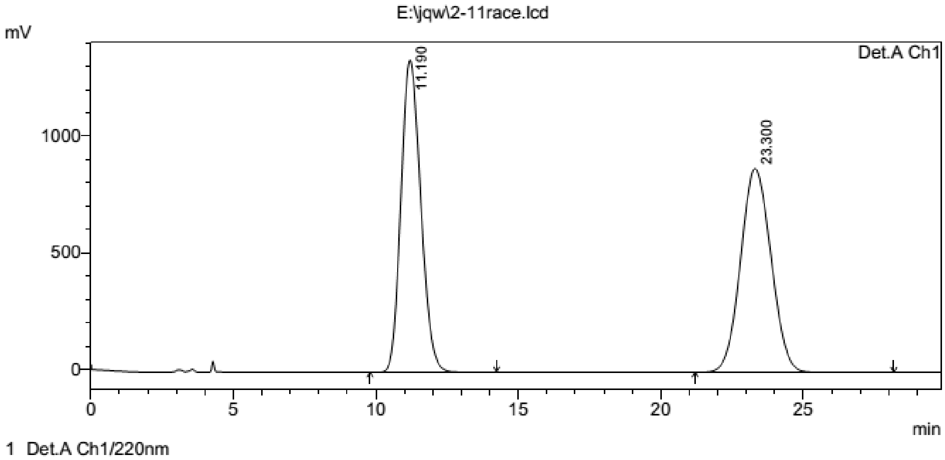

PeakTable

Detector A Ch1 220nm

Peak#	Ret. Time	Area	Height	Area %	Height %
1	11.190	64709636	1337691	49.925	60.502
2	23.300	64903555	873297	50.075	39.498
Total		129613192	2210989	100.000	100.000

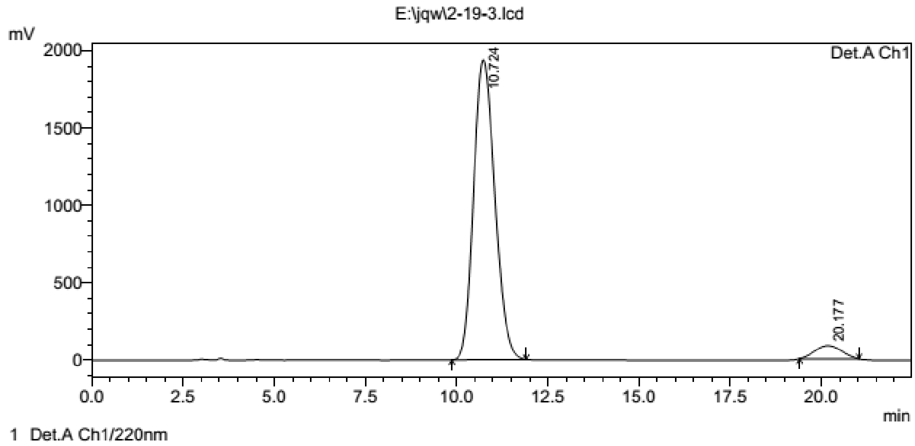

PeakTable

Detector A Ch1 220nm

Peak#	Ret. Time	Area	Height	Area %	Height %
1	10.724	78526764	1937772	94.800	95.834
2	20.177	4307236	84246	5.200	4.166
Total		82834000	2022018	100.000	100.000

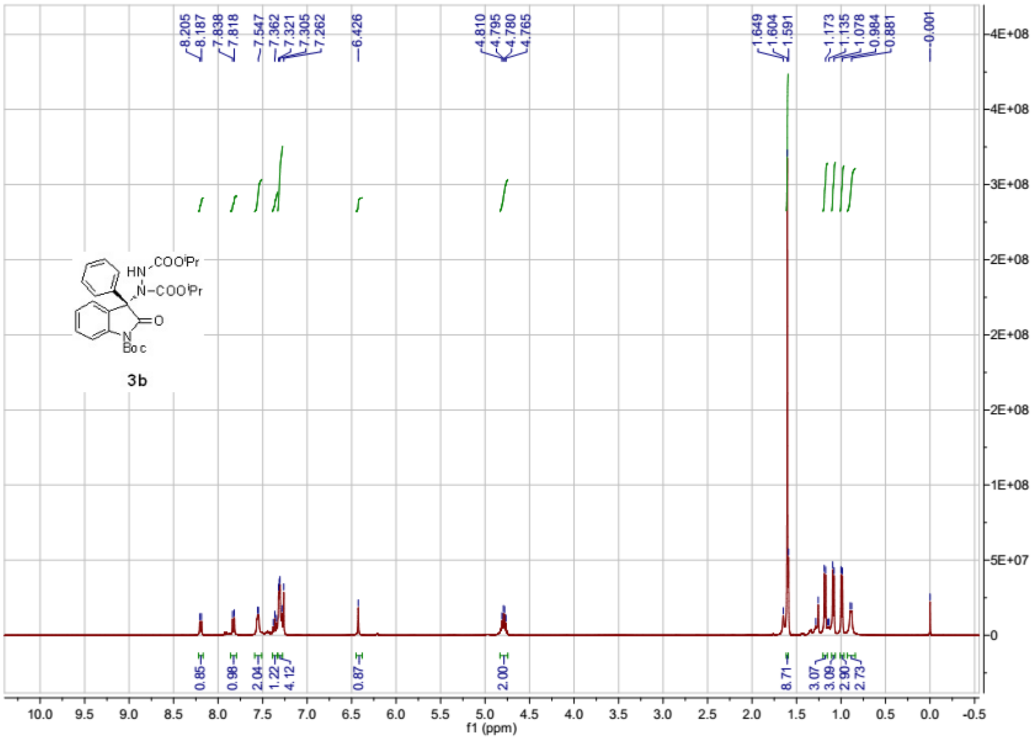

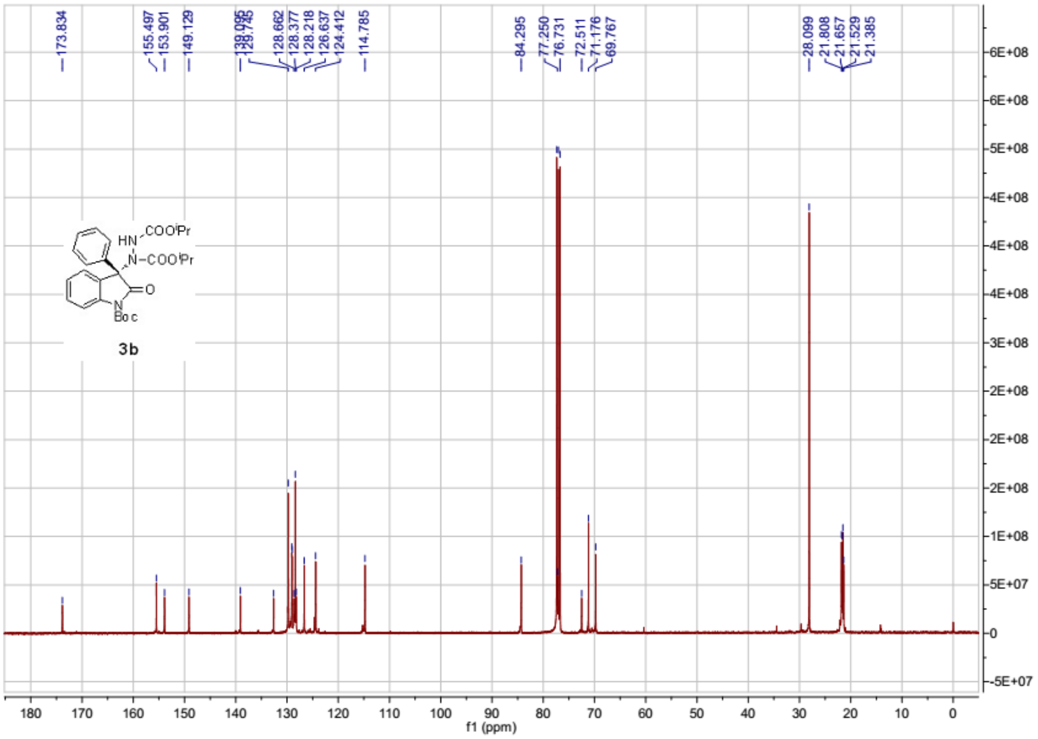

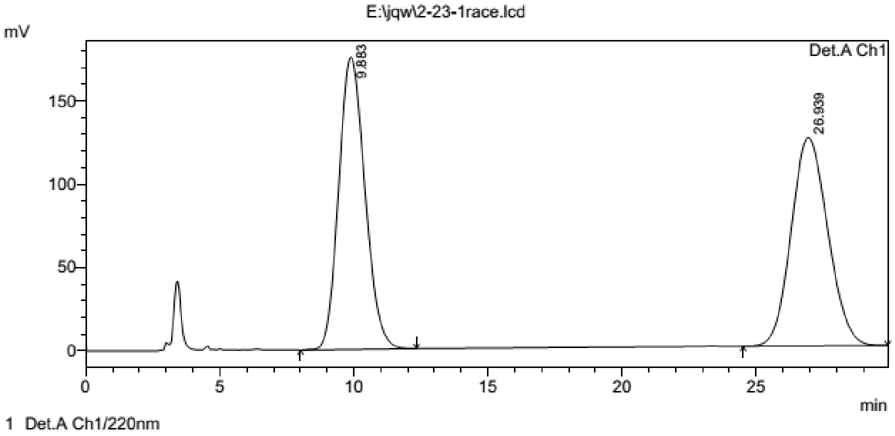

PeakTable

Detector A Ch1 220nm

Peak#	Ret. Time	Area	Height	Area %	Height %
1	9.883	11913078	175496	50.085	58.398
2	26.939	11872752	125024	49.915	41.602
Total		23785830	300520	100.000	100.000

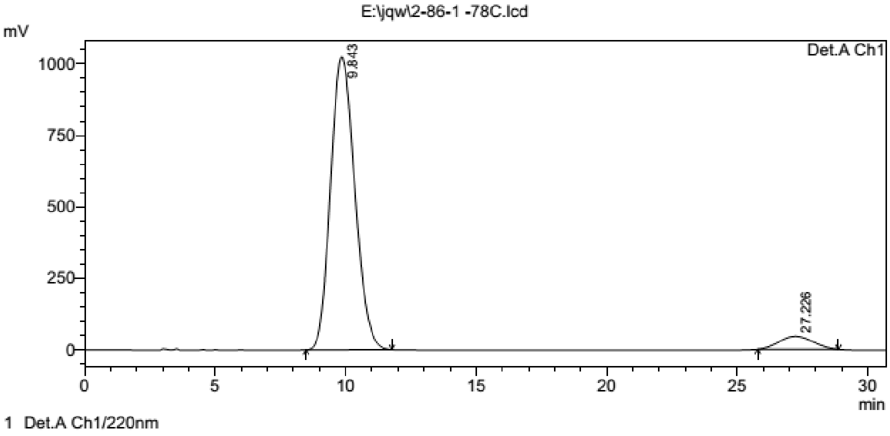

PeakTable

Detector A Ch1 220nm

Peak#	Ret. Time	Area	Height	Area %	Height %
1	9.843	66346369	1026071	94.211	95.849
2	27.226	4076782	44437	5.789	4.151
Total		70423151	1070508	100.000	100.000

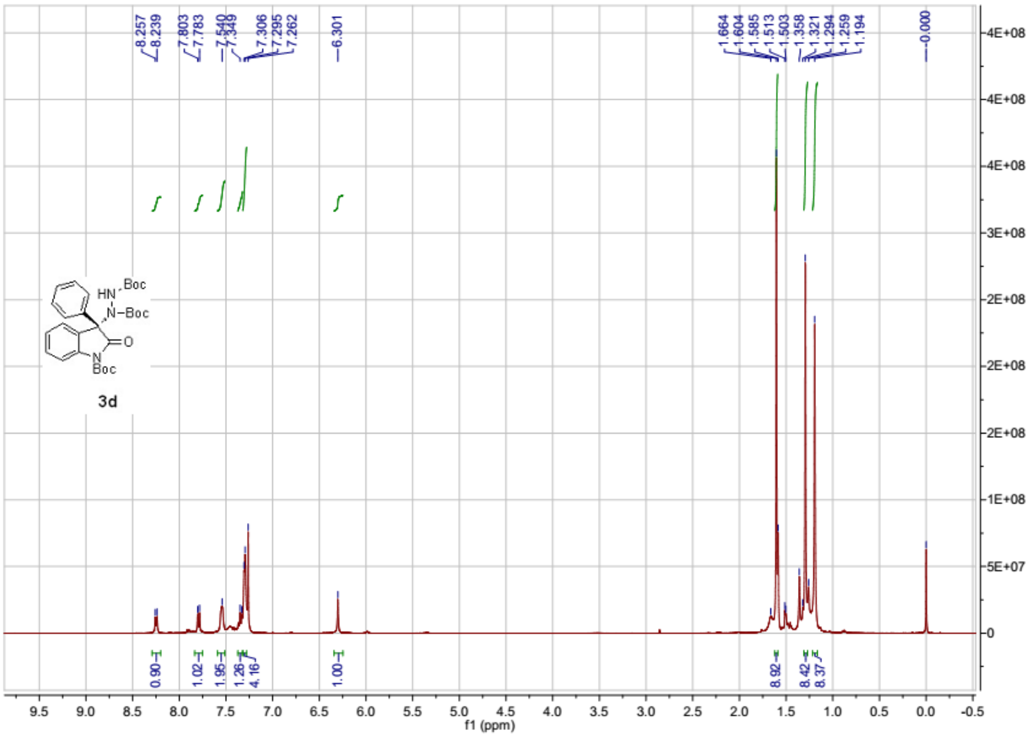

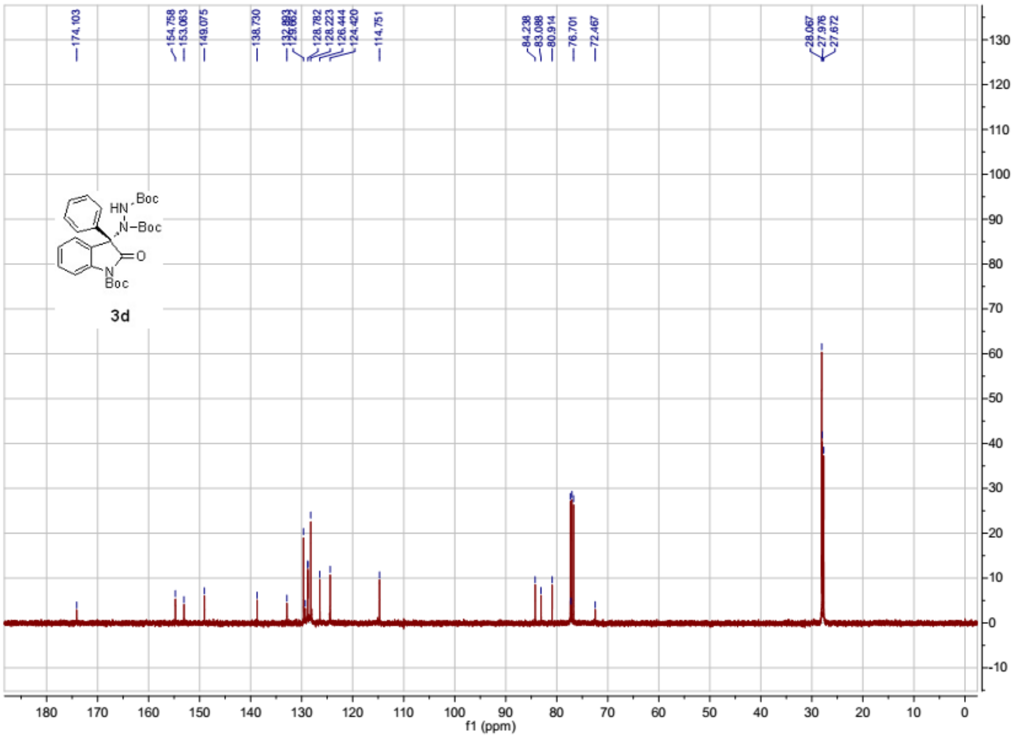

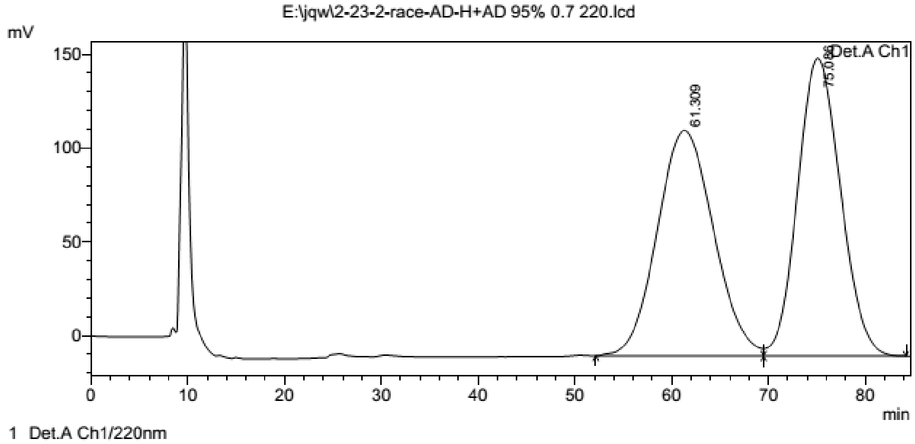

PeakTable

Detector A Ch1 220nm

Peak#	Ret. Time	Area	Height	Area %	Height %
1	61.309	48656036	120237	49.871	43.096
2	75.086	48907743	158764	50.129	56.904
Total		97563779	279001	100.000	100.000

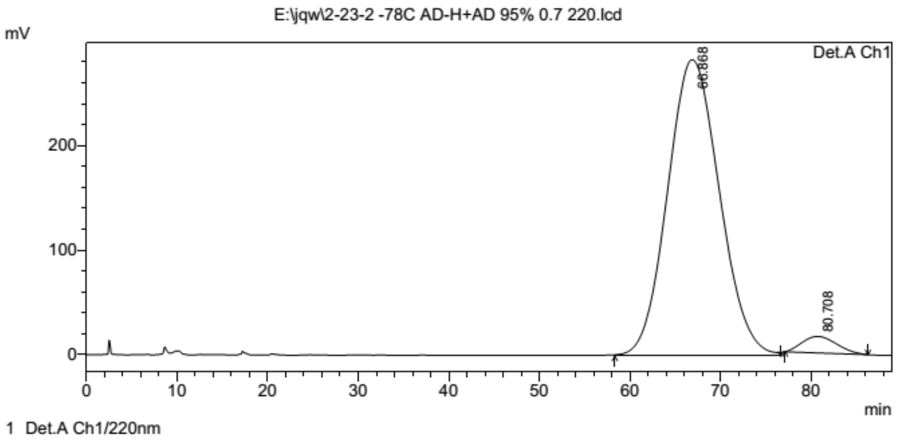

PeakTable

Detector A Ch1 220nm

Peak#	Ret. Time	Area	Height	Area %	Height %
1	66.868	113359519	283562	96.441	94.748
2	80.708	4183885	15719	3.559	5.252
Total		117543404	299282	100.000	100.000

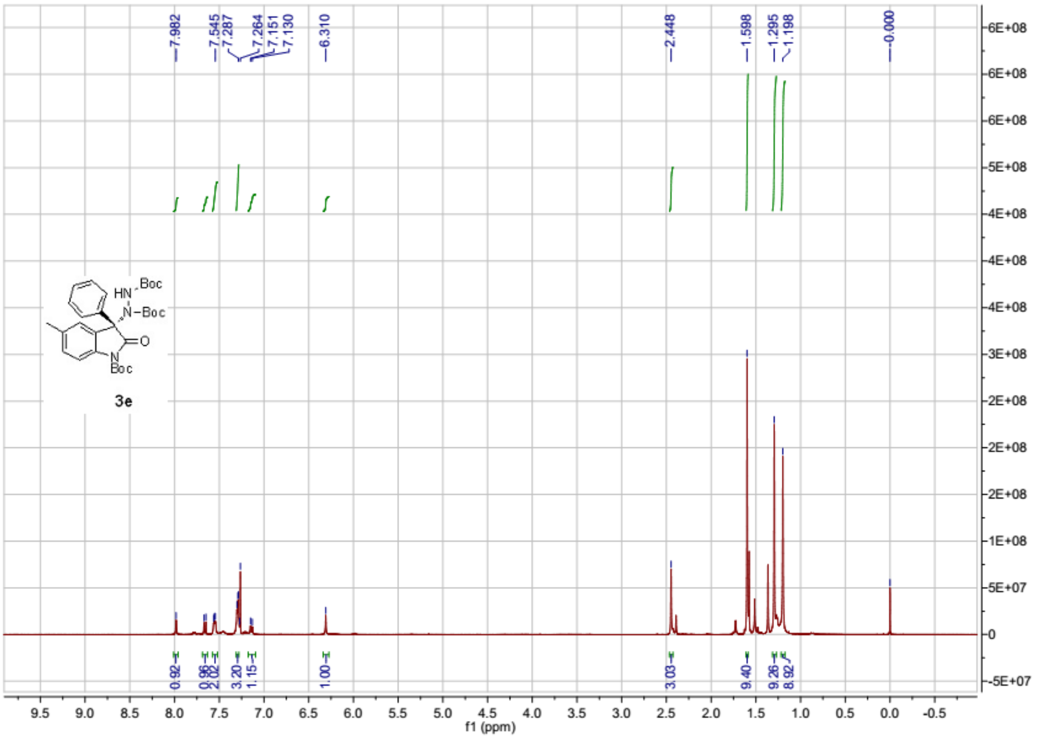

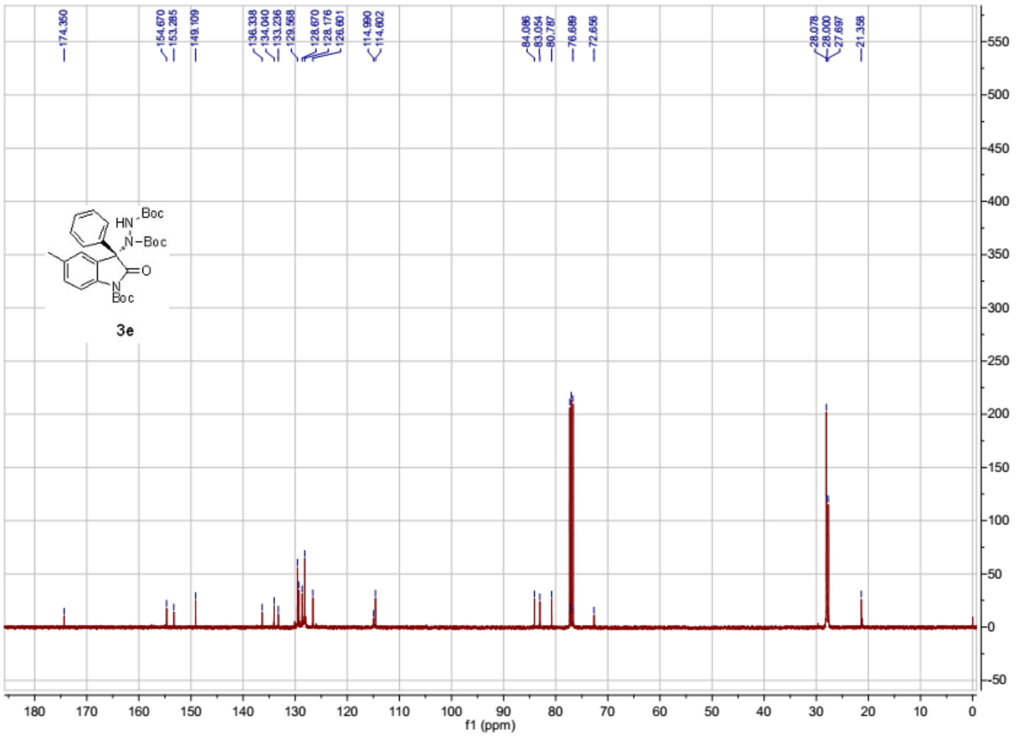

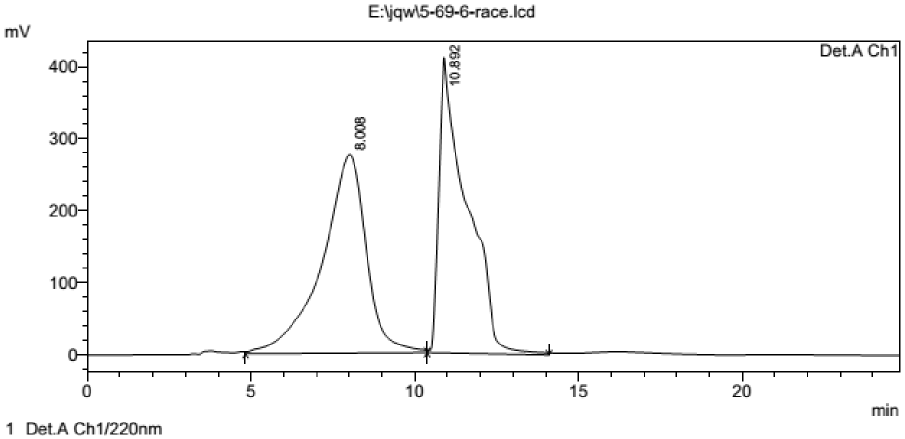

PeakTable

Detector A Ch1 220nm

Peak#	Ret. Time	Area	Height	Area %	Height %
1	8.008	25792986	275193	51.650	40.182
2	10.892	24144824	409669	48.350	59.818
Total		49937810	684862	100.000	100.000

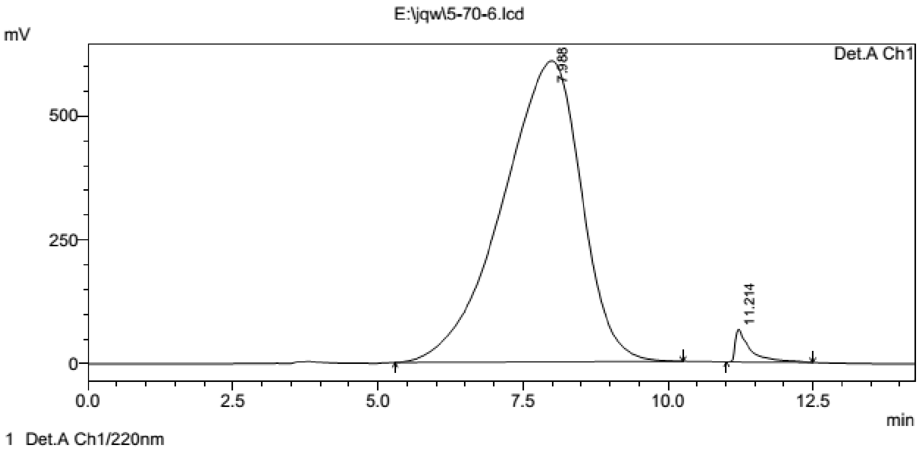

PeakTable

Detector A Ch1 220nm

Peak#	Ret. Time	Area	Height	Area %	Height %
1	7.988	57405358	607437	97.924	90.194
2	11.214	1217179	66044	2.076	9.806
Total		58622537	673480	100.000	100.000

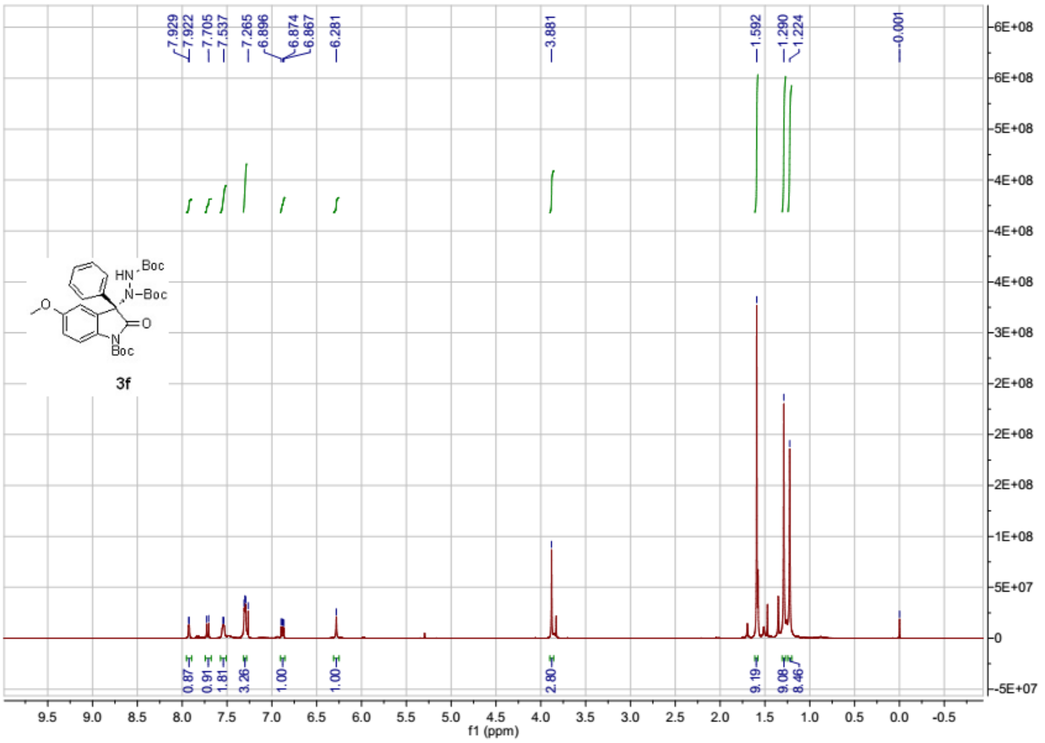

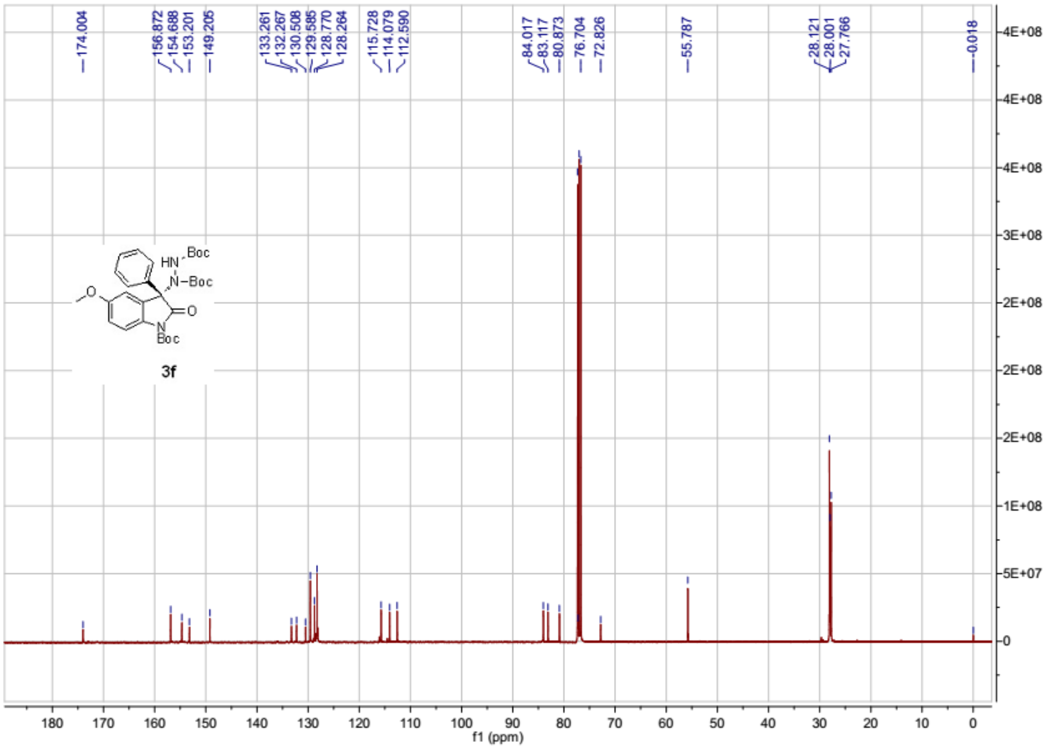

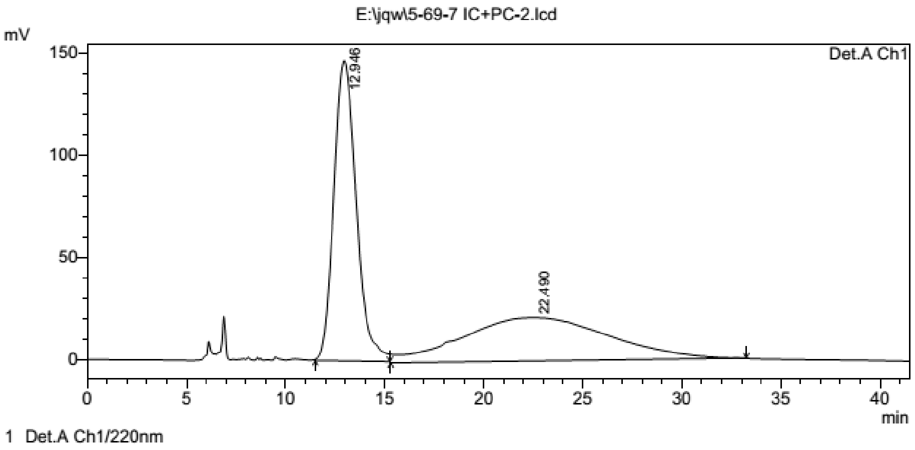

PeakTable

Detector A Ch1 220nm

Peak#	Ret. Time	Area	Height	Area %	Height %
1	12.946	11524836	146555	51.529	87.406
2	22.490	10840966	21117	48.471	12.594
Total		22365802	167672	100.000	100.000

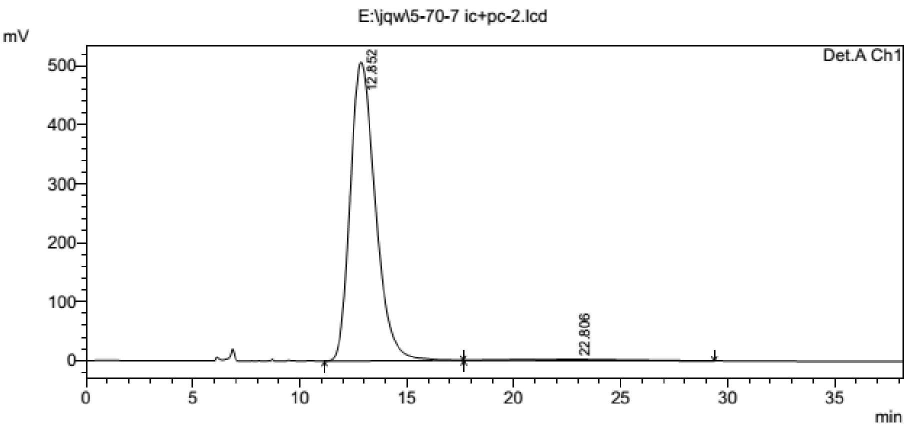

1 Det.A Ch1/220nm

PeakTable

Detector A Ch1 220nm

Peak#	Ret. Time	Area	Height	Area %	Height %
1	12.852	41610887	507128	98.140	99.582
2	22.806	788736	2127	1.860	0.418
Total		42399623	509255	100.000	100.000

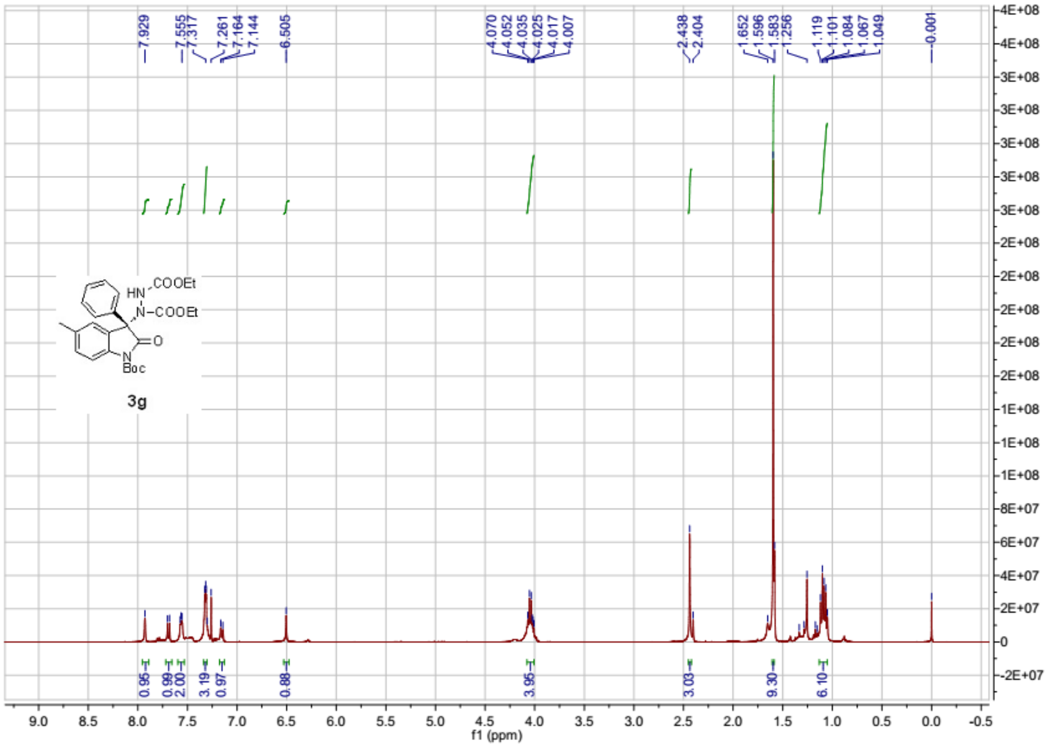

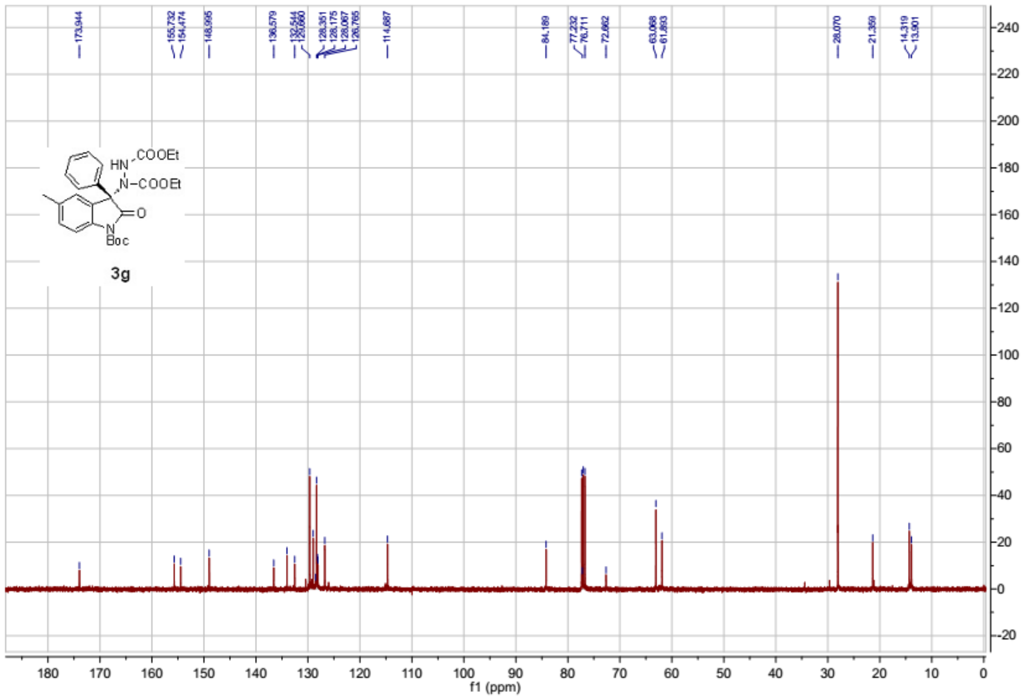

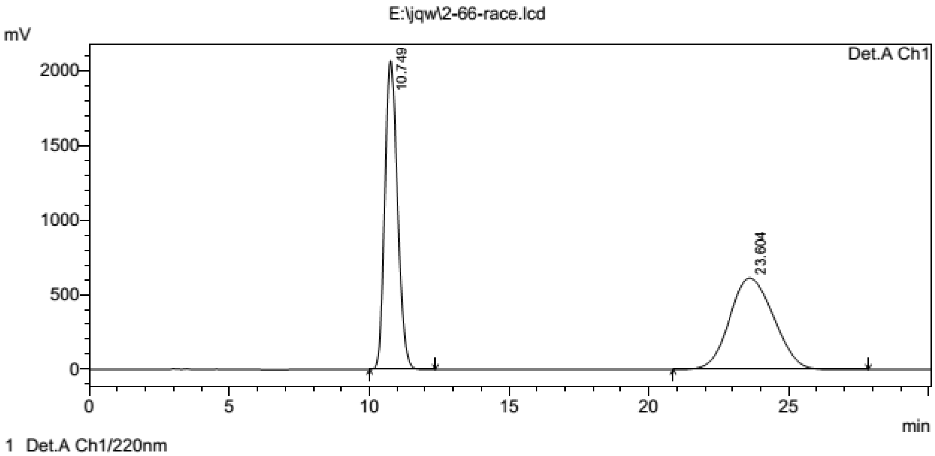

PeakTable

Detector A Ch1 220nm

Peak#	Ret. Time	Area	Height	Area %	Height %
1	10.749	66252054	2067814	49.719	77.230
2	23.604	67001408	609667	50.281	22.770
Total		133253461	2677481	100.000	100.000

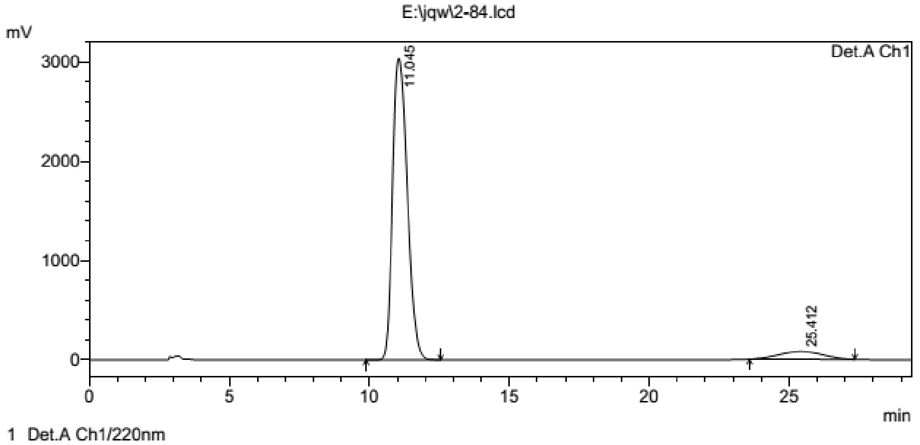

1 Det.A Ch1/220nm

PeakTable

Detector A Ch1 220nm

Peak#	Ret. Time	Area	Height	Area %	Height %
1	11.045	110470886	3034828	92.843	97.465
2	25.412	8515726	78943	7.157	2.535
Total		118986612	3113770	100.000	100.000

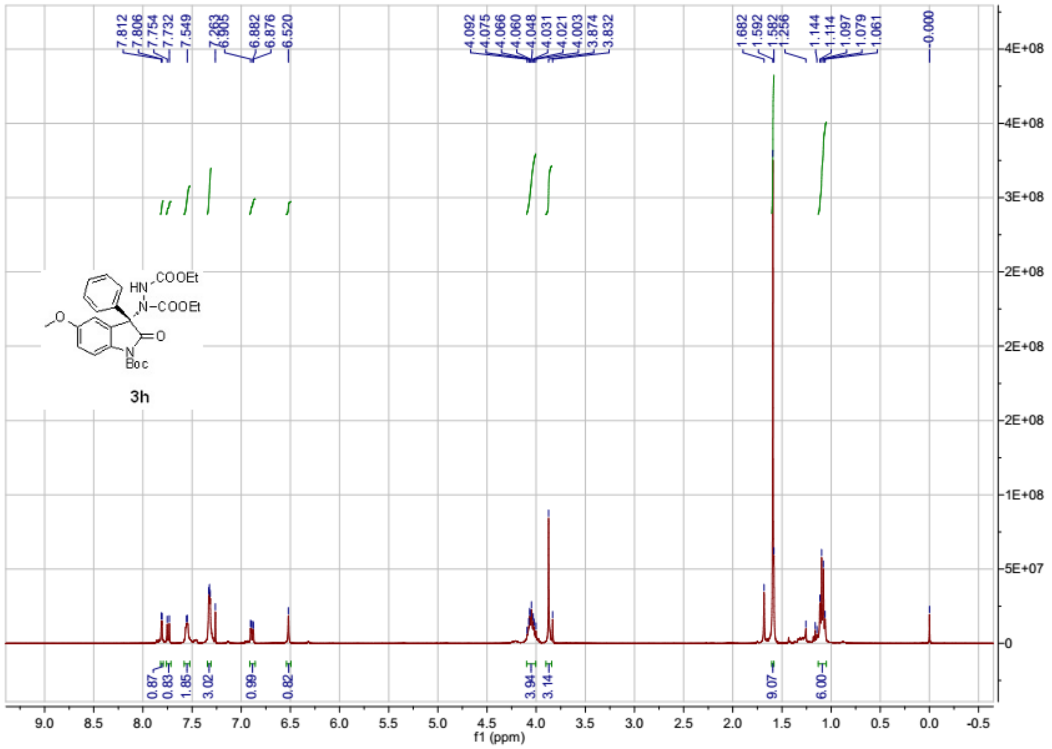

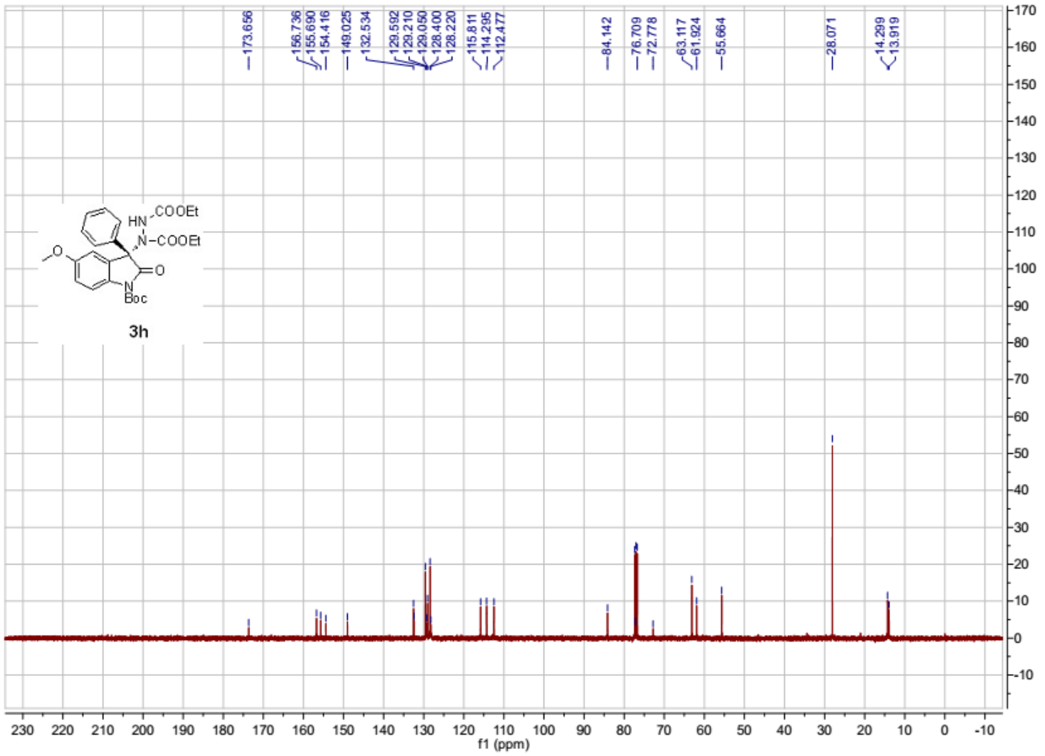

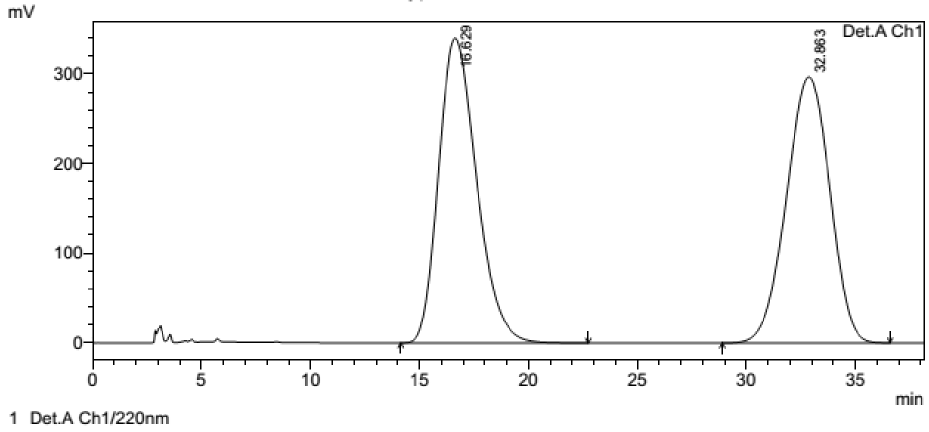

PeakTable

Detector A Ch1 220nm

Peak#	Ret. Time	Area	Height	Area %	Height %
1	16.629	41258138	340133	50.010	53.382
2	32.863	41242413	297030	49.990	46.618
Total		82500552	637163	100.000	100.000

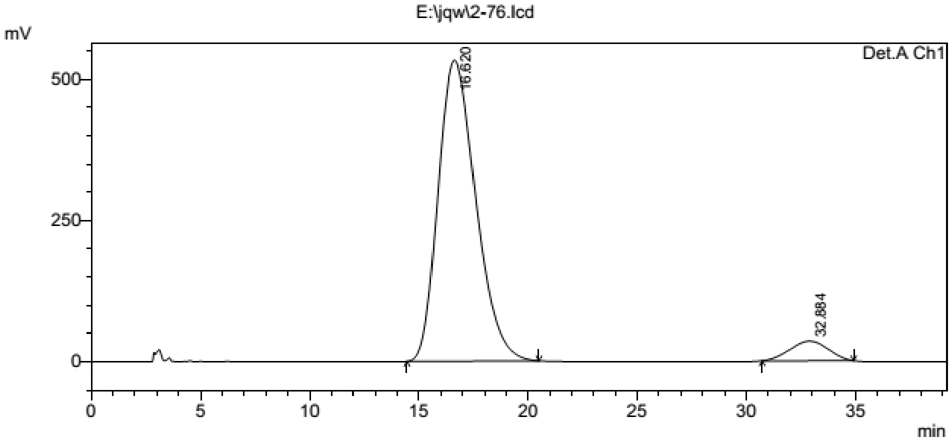

1 Det.A Ch1/220nm

PeakTable

Detector A Ch1 220nm

Peak#	Ret. Time	Area	Height	Area %	Height %
1	16.620	63903980	532515	93.728	93.834
2	32.884	4276482	34993	6.272	6.166
Total		68180462	567508	100.000	100.000

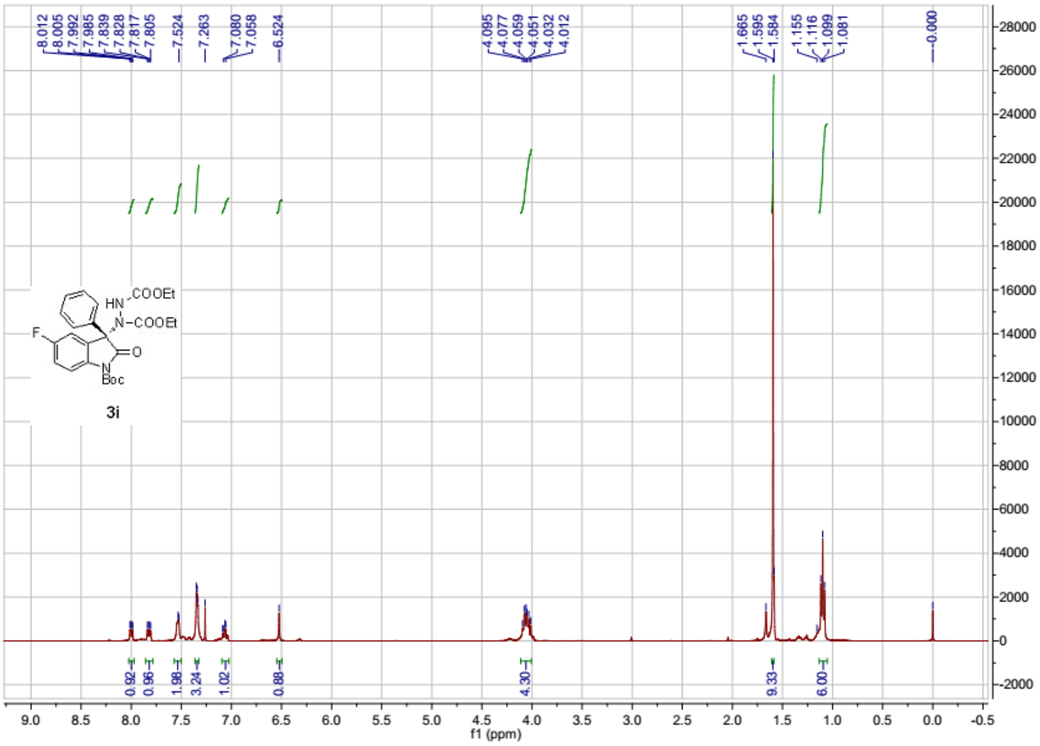

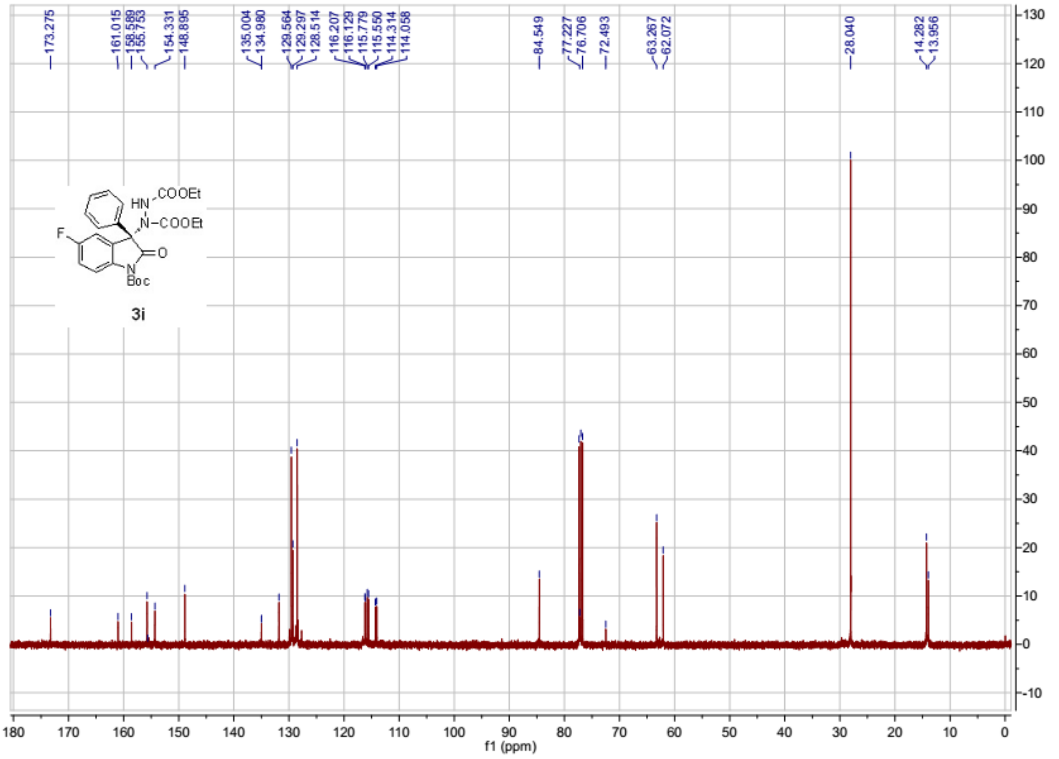

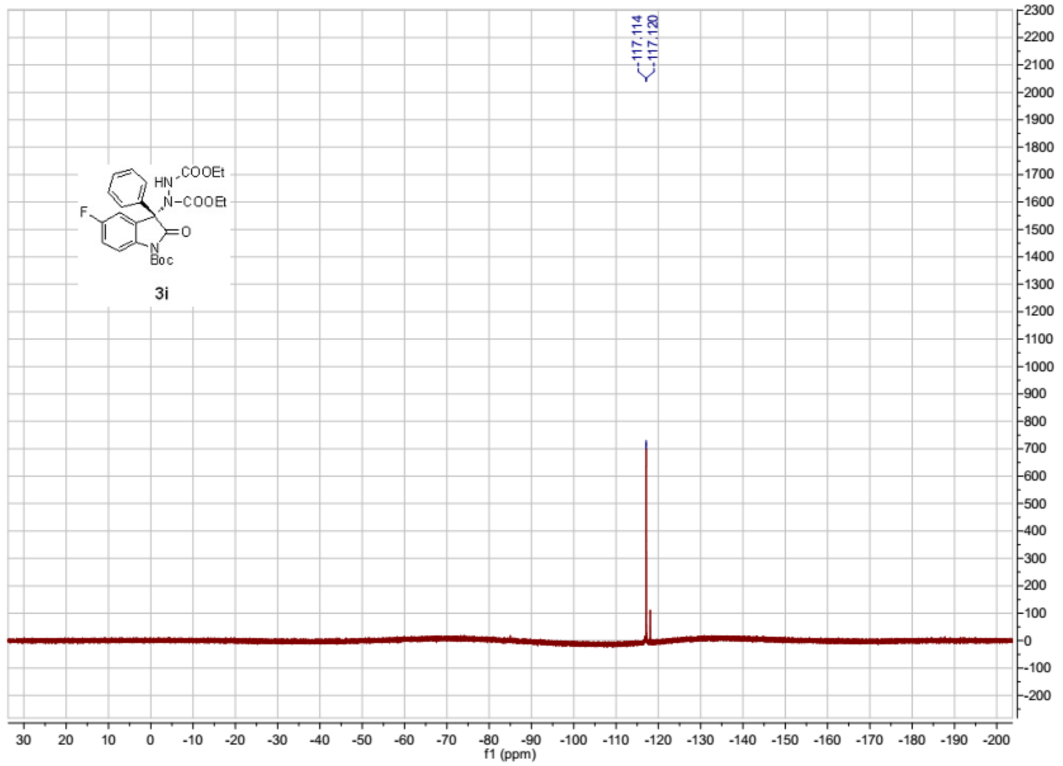

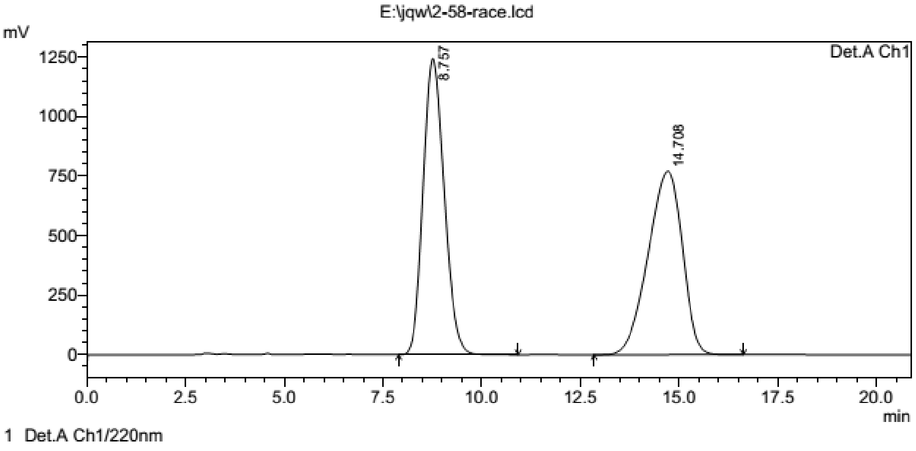

PeakTable

Detector A Ch1 220nm

Peak#	Ret. Time	Area	Height	Area %	Height %
1	8.757	46393494	1244194	49.942	61.743
2	14.708	46501695	770911	50.058	38.257
Total		92895189	2015105	100.000	100.000

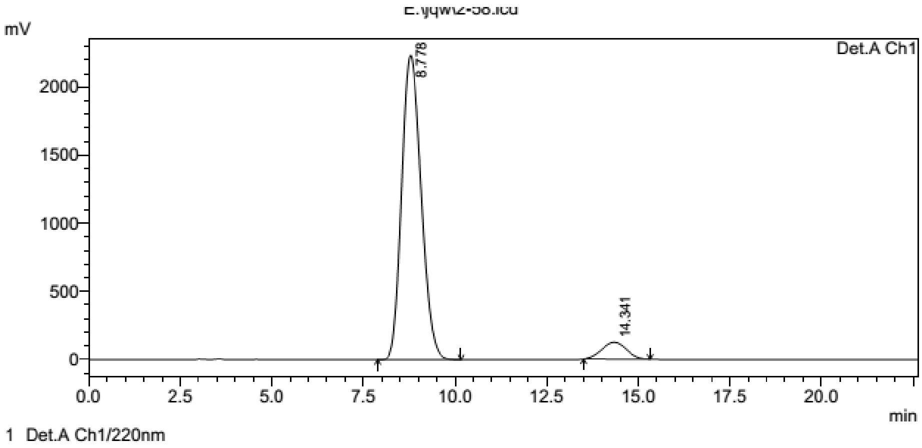

PeakTable

Detector A Ch1 220nm

Peak#	Ret. Time	Area	Height	Area %	Height %
1	8.778	82857819	2232793	93.450	94.751
2	14.341	5807272	123701	6.550	5.249
Total		88665091	2356494	100.000	100.000

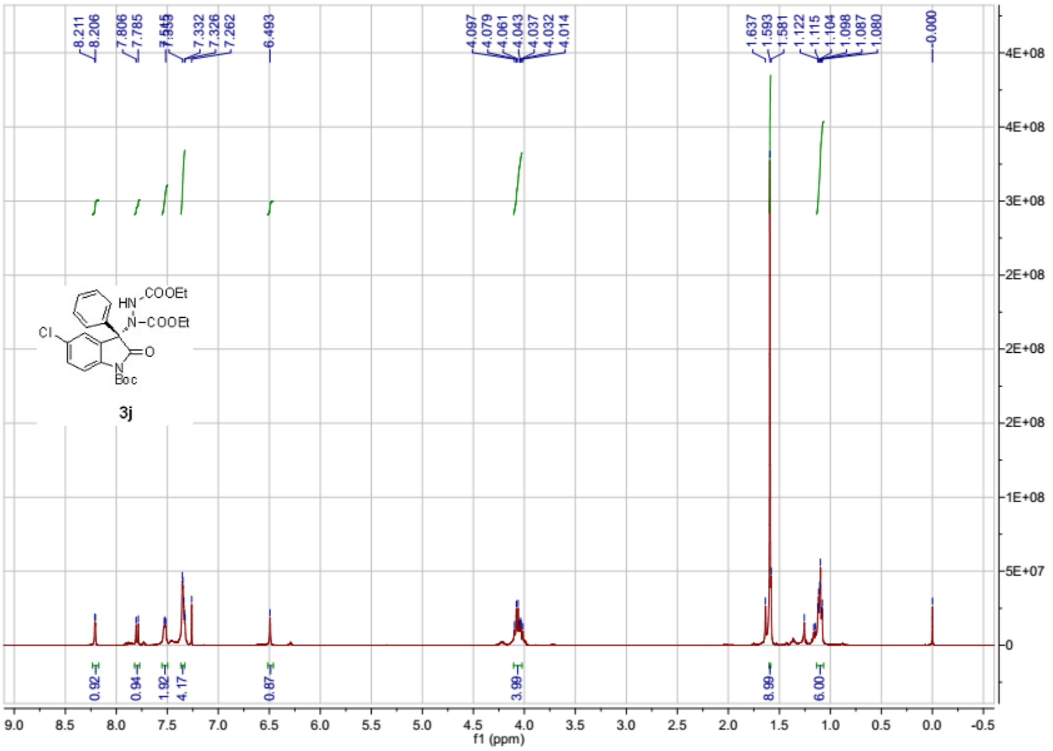

8.211
8.206
7.806
7.785
7.332
7.326
7.262
6.493
4.097
4.079
4.061
4.043
4.037
4.032
4.014
1.637
1.593
1.581
1.122
1.115
1.104
1.098
1.087
1.080
0.000
0.92
0.94
1.92
4.17
0.87
3.99
8.99
6.00
f1 (ppm)
3j

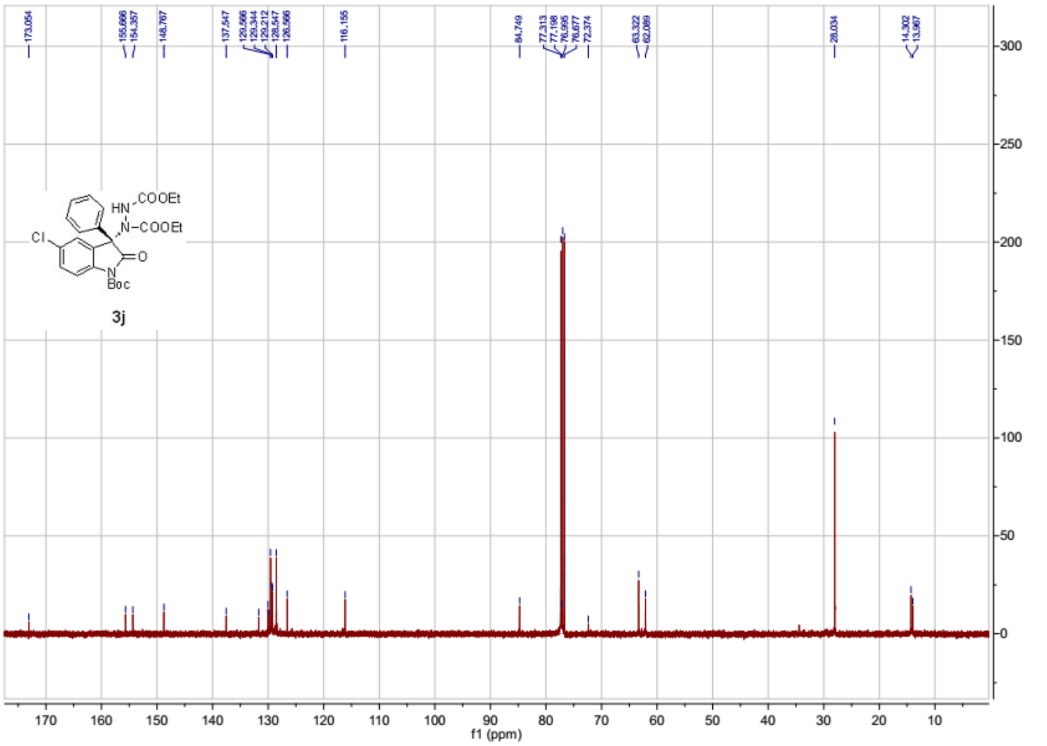

173.054
155.666
154.357
148.767
137.547
129.566
129.344
129.212
128.547
126.566
116.155
84.749
77.313
77.198
76.995
76.677
72.374
63.322
62.089
28.034
14.302
13.967
f1 (ppm)
3j

E:\jqw\3-1-2-race.lcd

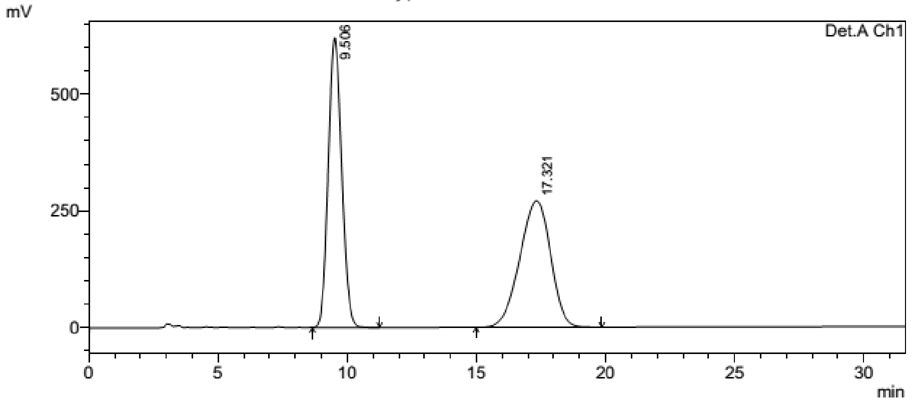

1 Det.A Ch1/220nm

PeakTable

Detector A Ch1 220nm

Peak#	Ret. Time	Area	Height	Area %	Height %
1	9.506	22504660	621001	50.009	69.567
2	17.321	22496519	271661	49.991	30.433
Total		45001178	892662	100.000	100.000

E:\jqw\3-1-2.lcd

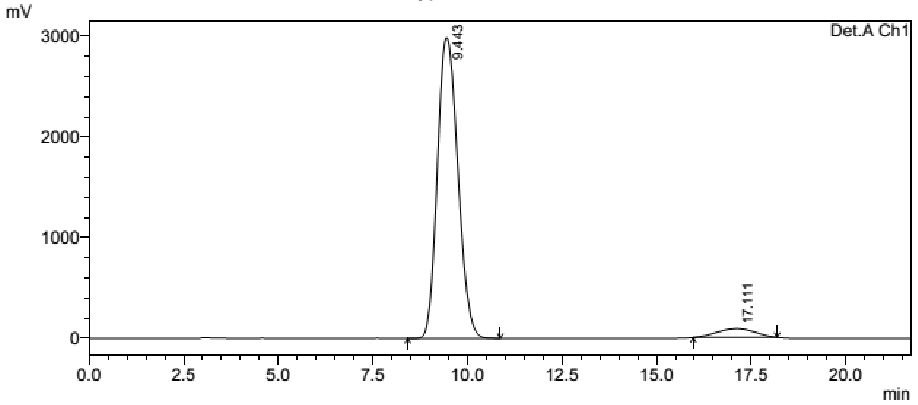

1 Det.A Ch1/220nm

PeakTable

Detector A Ch1 220nm

Peak#	Ret. Time	Area	Height	Area %	Height %
1	9.443	112516207	2986054	94.767	97.066
2	17.111	6212942	90264	5.233	2.934
Total		118729149	3076318	100.000	100.000

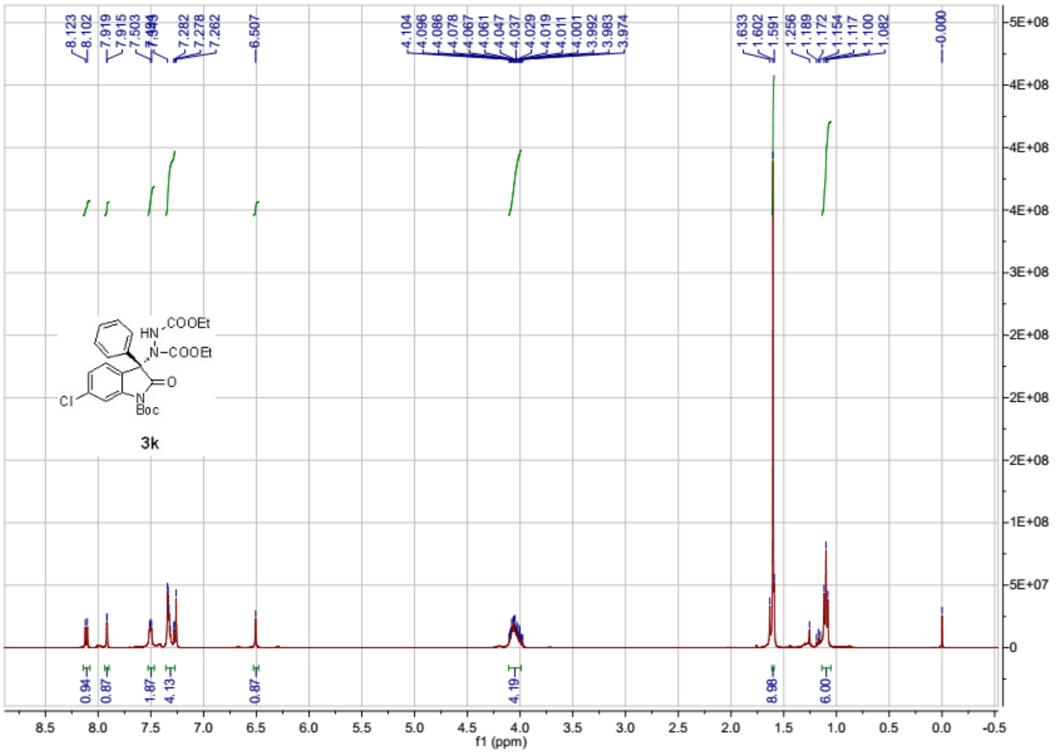

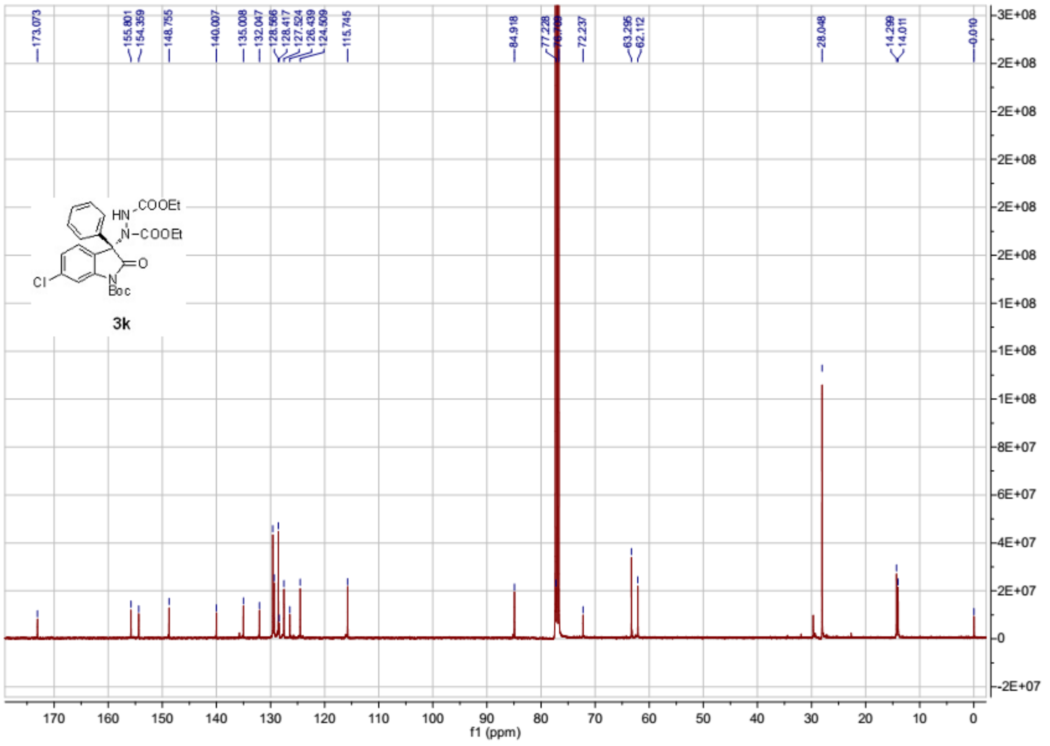

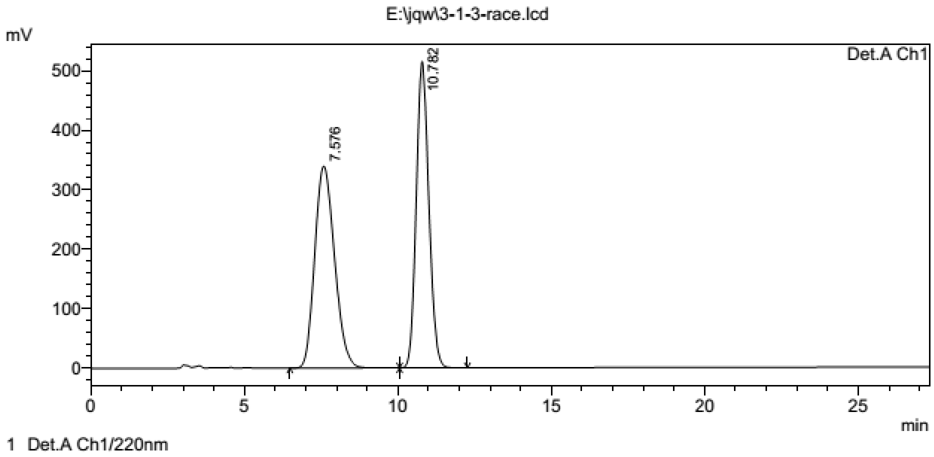

PeakTable

Detector A Ch1 220nm

Peak#	Ret. Time	Area	Height	Area %	Height %
1	7.576	15121633	339302	50.090	39.735
2	10.782	15067024	514612	49.910	60.265
Total		30188657	853914	100.000	100.000

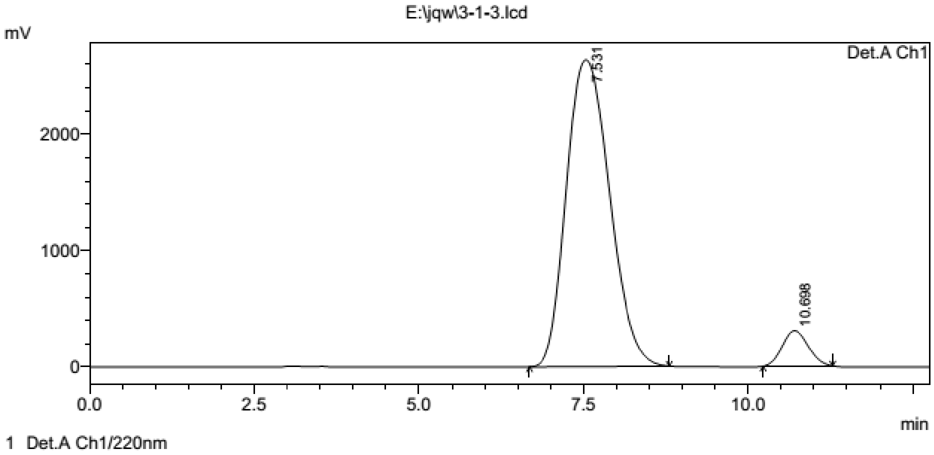

PeakTable

Detector A Ch1 220nm

Peak#	Ret. Time	Area	Height	Area %	Height %
1	7.531	120465199	2637583	93.413	89.576
2	10.698	8494258	306938	6.587	10.424
Total		128959456	2944521	100.000	100.000

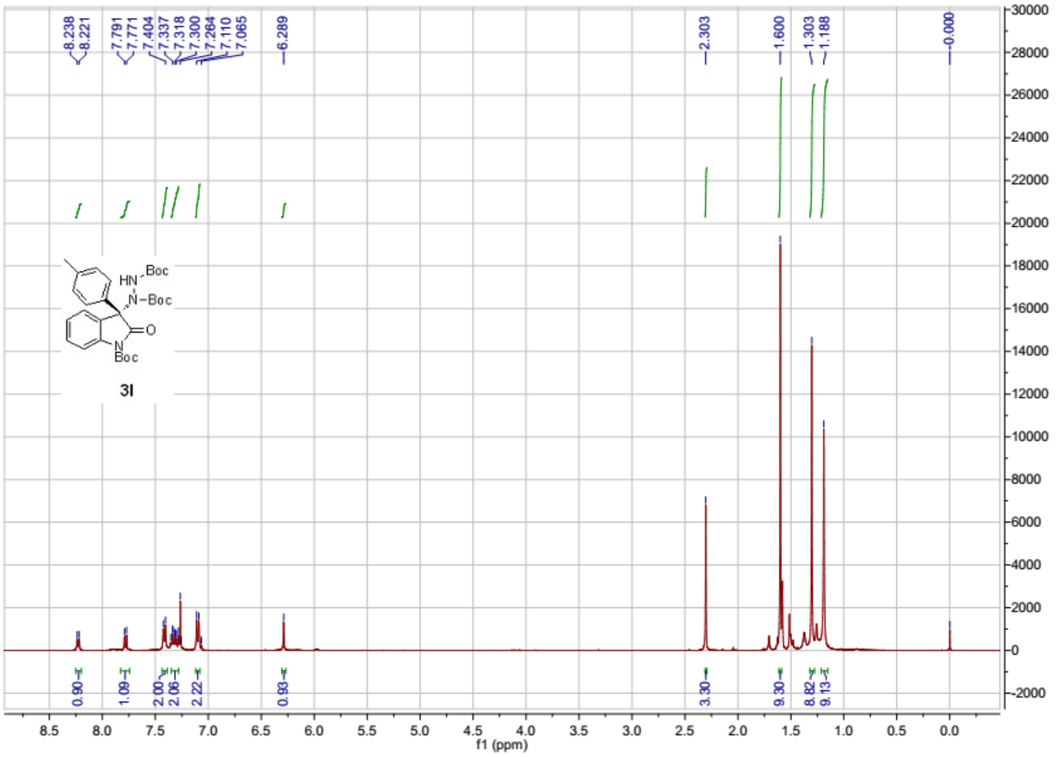
8.238
8.221
7.791
7.771
7.404
7.337
7.318
7.300
7.264
7.110
7.065
6.289
2.303
1.600
1.303
1.188
0.000
0.90
1.09
2.00
2.06
2.22
0.93
3.30
9.30
8.82
9.13
HN
Boc
N
Boc
O
N
Boc
3l
f1 (ppm)

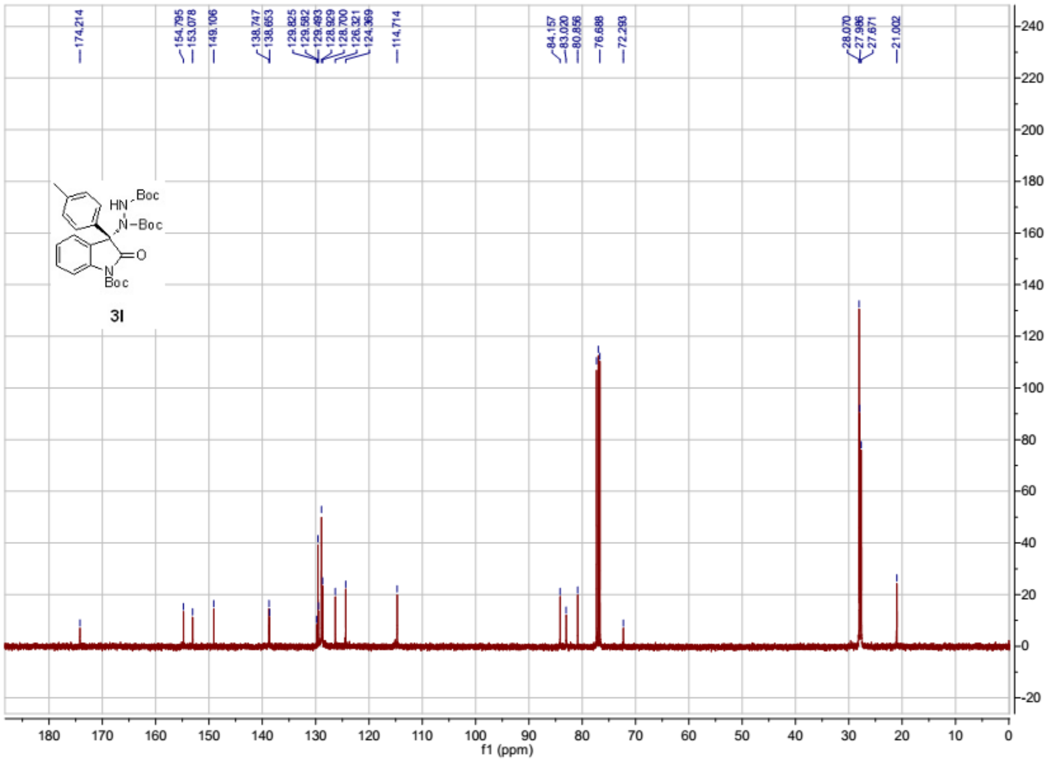
174.214
154.795
153.078
149.106
138.747
138.653
129.825
129.582
129.493
128.929
128.700
126.321
124.369
114.714
84.157
83.020
80.856
76.688
72.293
28.070
27.986
27.671
21.002
HN
Boc
N
Boc
O
N
Boc
3l
f1 (ppm)

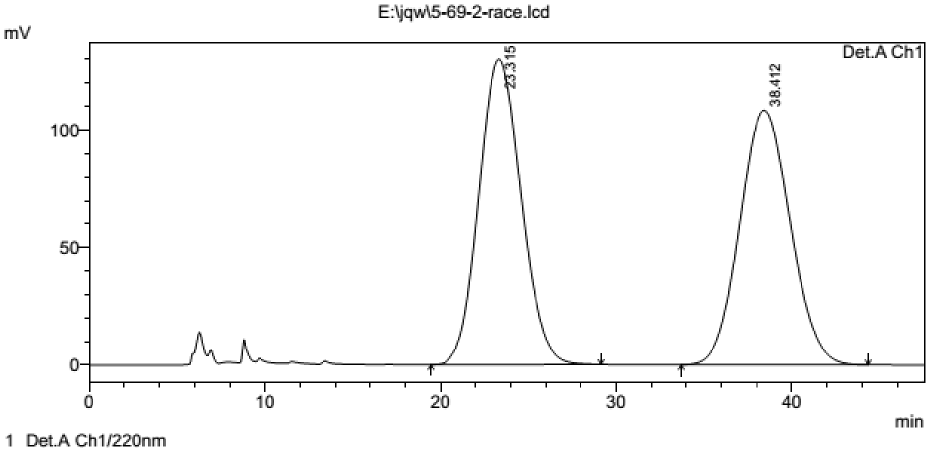

PeakTable

Detector A Ch1 220nm

Peak#	Ret. Time	Area	Height	Area %	Height %
1	23.315	21773663	129947	50.102	54.557
2	38.412	21684809	108238	49.898	45.443
Total		43458472	238185	100.000	100.000

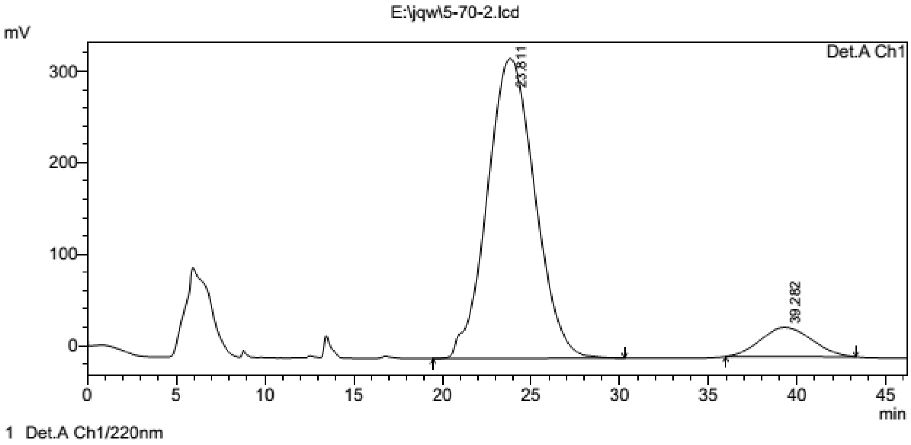

PeakTable

Detector A Ch1 220nm

Peak#	Ret. Time	Area	Height	Area %	Height %
1	23.811	61165017	327755	90.360	91.082
2	39.282	6524984	32090	9.640	8.918
Total		67690001	359845	100.000	100.000

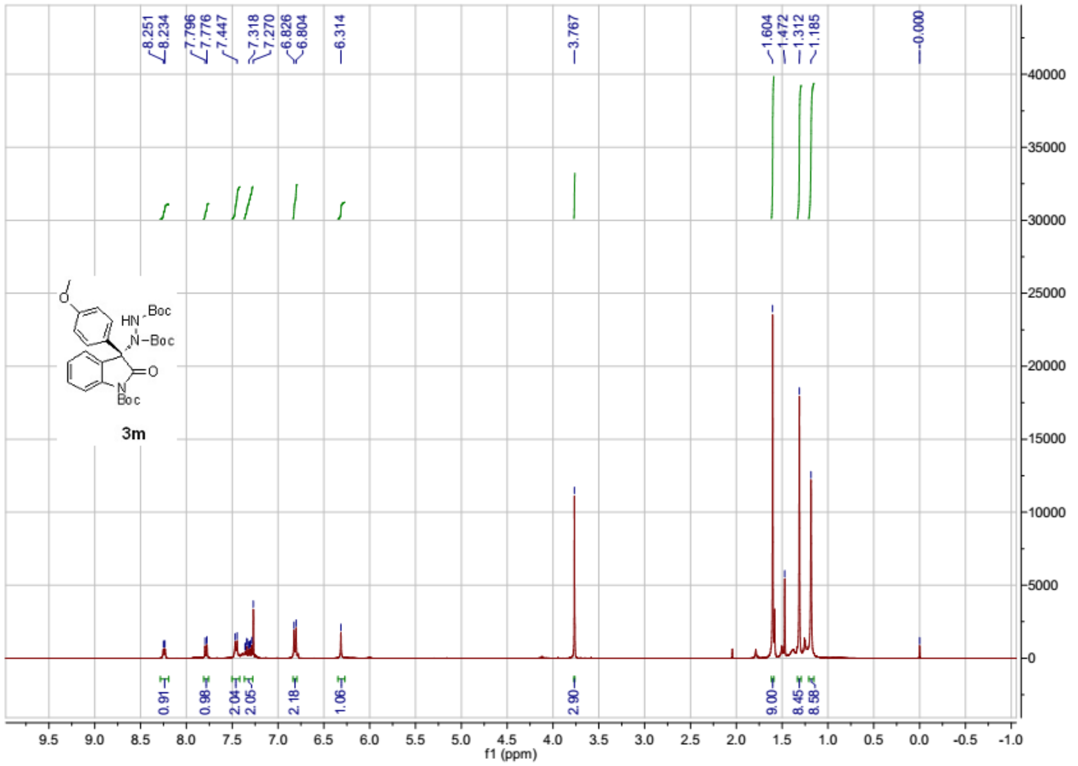

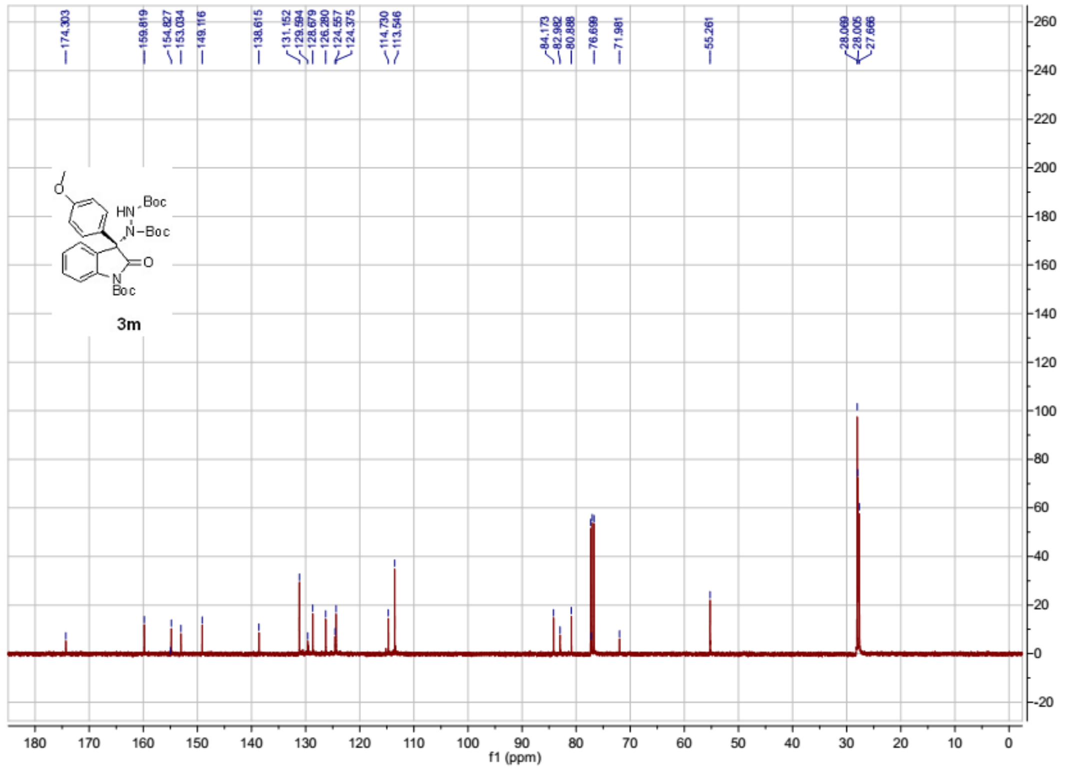

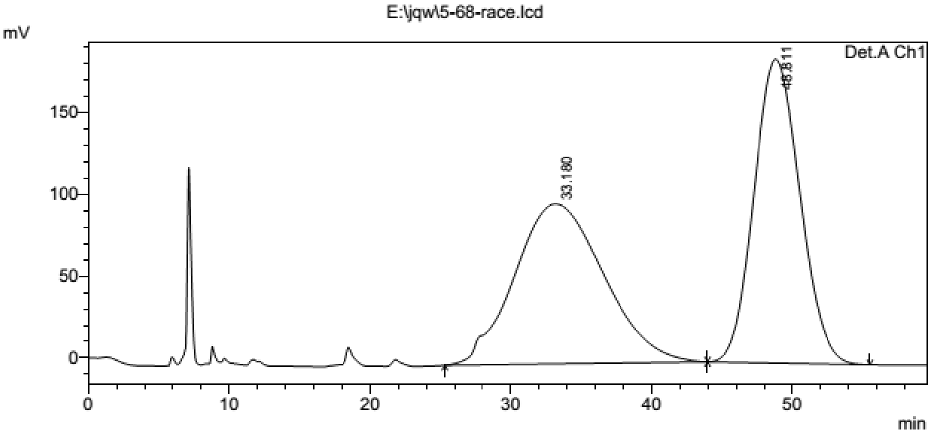

1 Det.A Ch1/220nm

PeakTable

Detector A Ch1 220nm

Peak#	Ret. Time	Area	Height	Area %	Height %
1	33.180	43046283	97804	50.495	34.497
2	48.811	42202533	185712	49.505	65.503
Total		85248816	283517	100.000	100.000

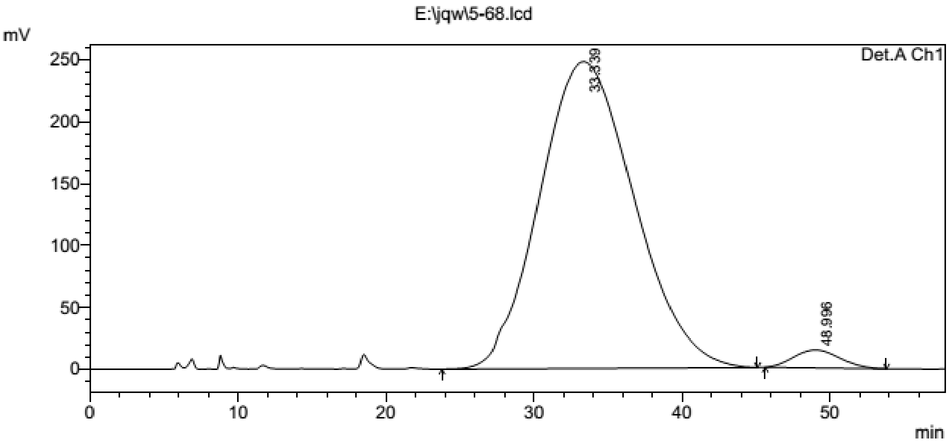

1 Det.A Ch1/220nm

PeakTable

Detector A Ch1 220nm

Peak#	Ret. Time	Area	Height	Area %	Height %
1	33.339	110287628	247599	97.207	94.442
2	48.996	3168805	14573	2.793	5.558
Total		113456433	262172	100.000	100.000

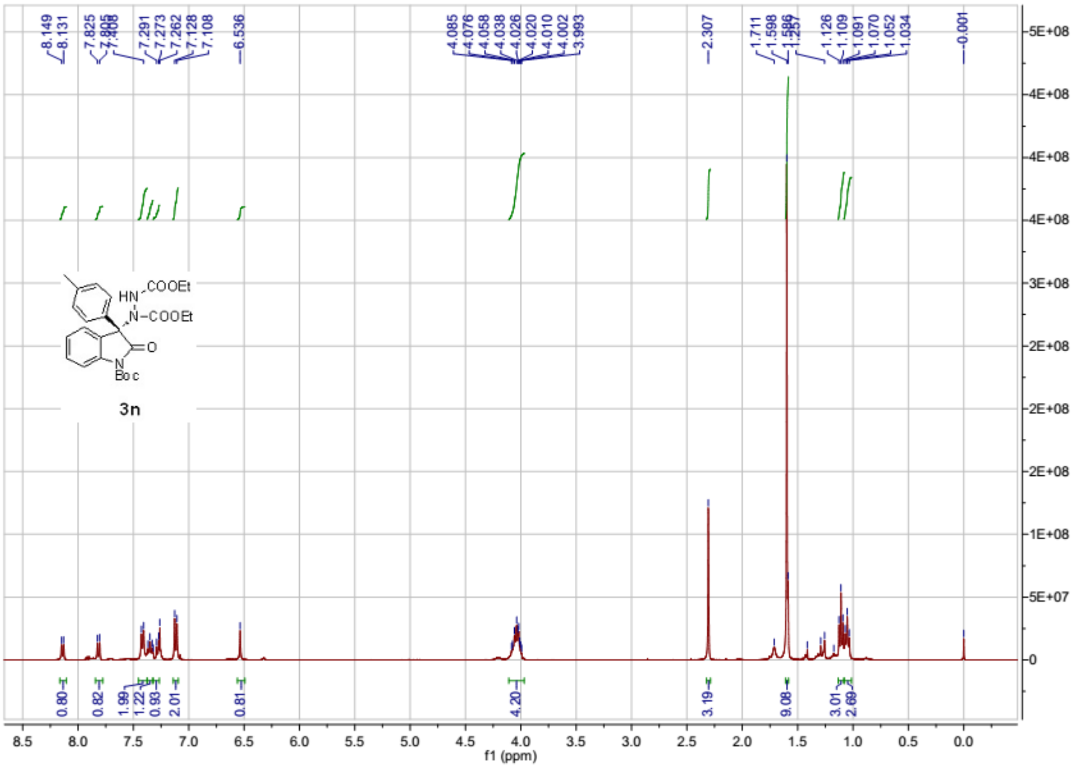
8.149
8.131
7.825
7.291
7.273
7.262
7.128
7.108
6.536
4.085
4.076
4.058
4.038
4.026
4.020
4.010
4.002
3.993
2.307
1.711
1.598
1.126
1.109
1.091
1.070
1.052
1.034
0.001
HN-COOEt
N-COOEt
O
N
Boc
3n
0.80
0.82
1.99
1.22
0.93
2.01
0.81
4.20
3.19
9.08
3.01
2.69
f1 (ppm)

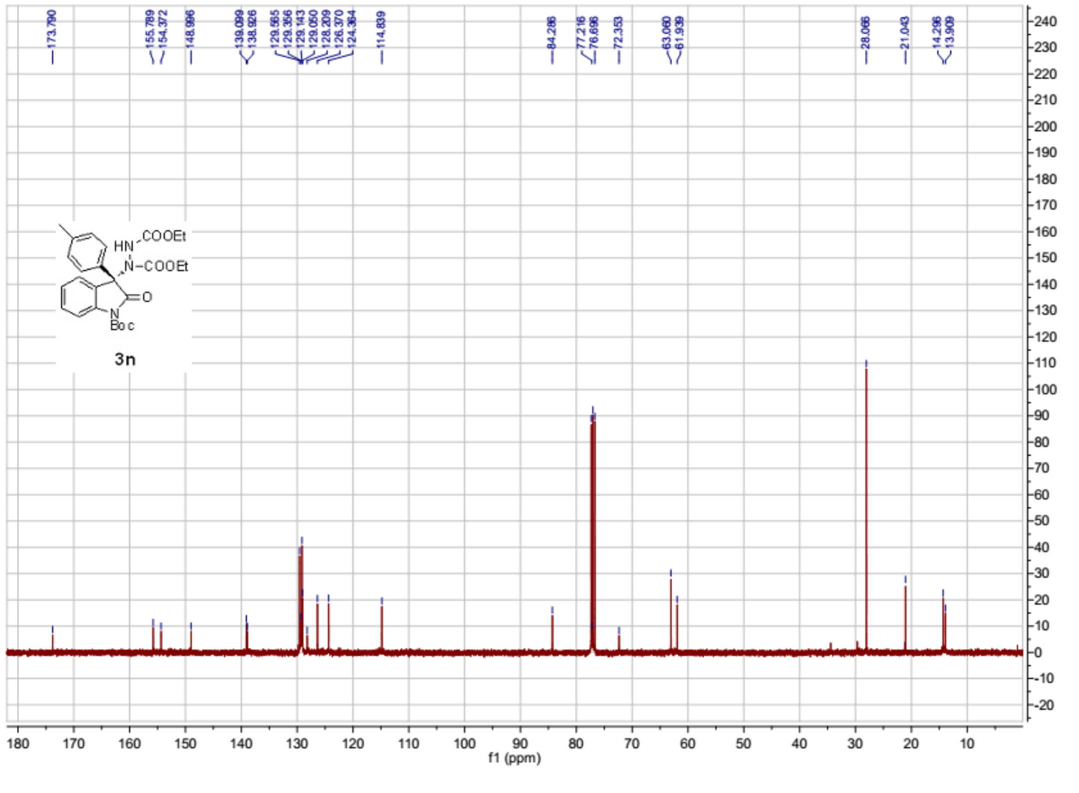
173.790
155.789
154.372
148.996
139.099
138.926
129.565
129.356
129.143
129.050
128.209
126.370
124.364
114.839
84.286
77.216
76.696
72.353
63.060
61.939
28.066
21.043
14.296
13.909
HN-COOEt
N-COOEt
O
N
Boc
3n
f1 (ppm)

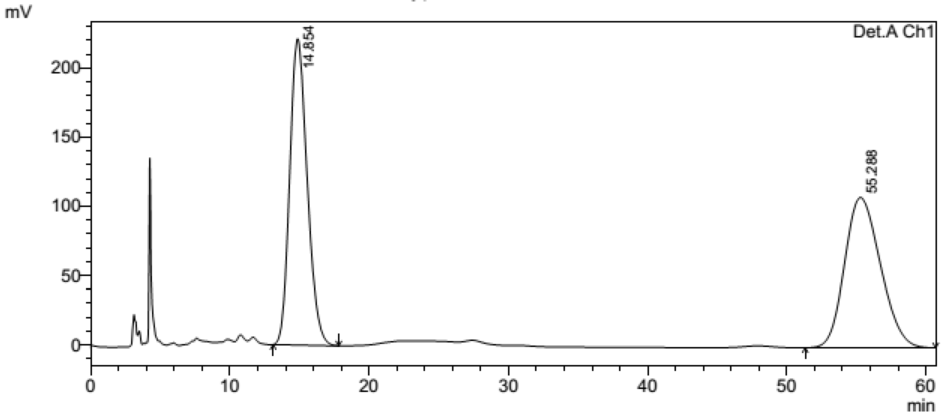

1 Det.A Ch1/220nm

PeakTable

Detector A Ch1 220nm

Peak#	Ret. Time	Area	Height	Area %	Height %
1	14.854	19372119	221482	49.904	67.119
2	55.288	19446710	108501	50.096	32.881
Total		38818829	329983	100.000	100.000

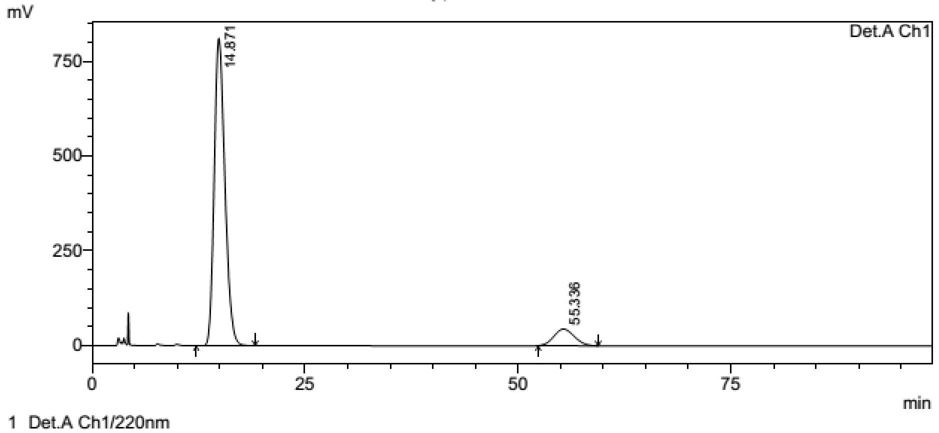

1 Det.A Ch1/220nm

PeakTable

Detector A Ch1 220nm

Peak#	Ret. Time	Area	Height	Area %	Height %
1	14.871	70380231	810317	90.655	94.899
2	55.336	7255430	43557	9.345	5.101
Total		77635660	853874	100.000	100.000

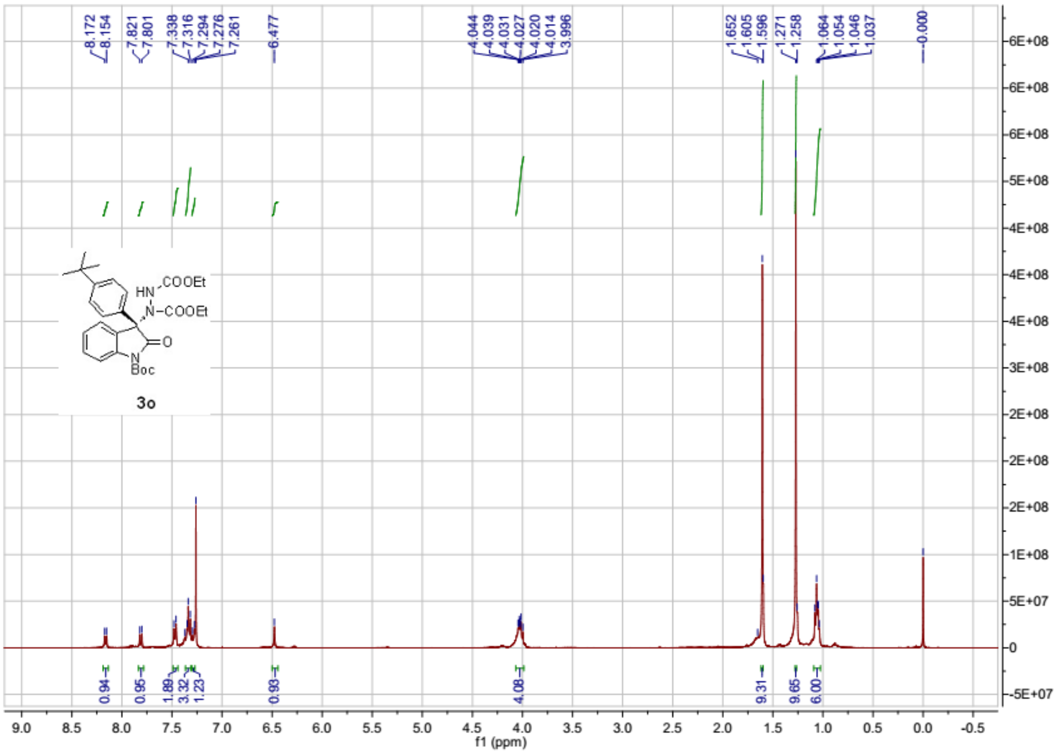

3o
f1 (ppm)

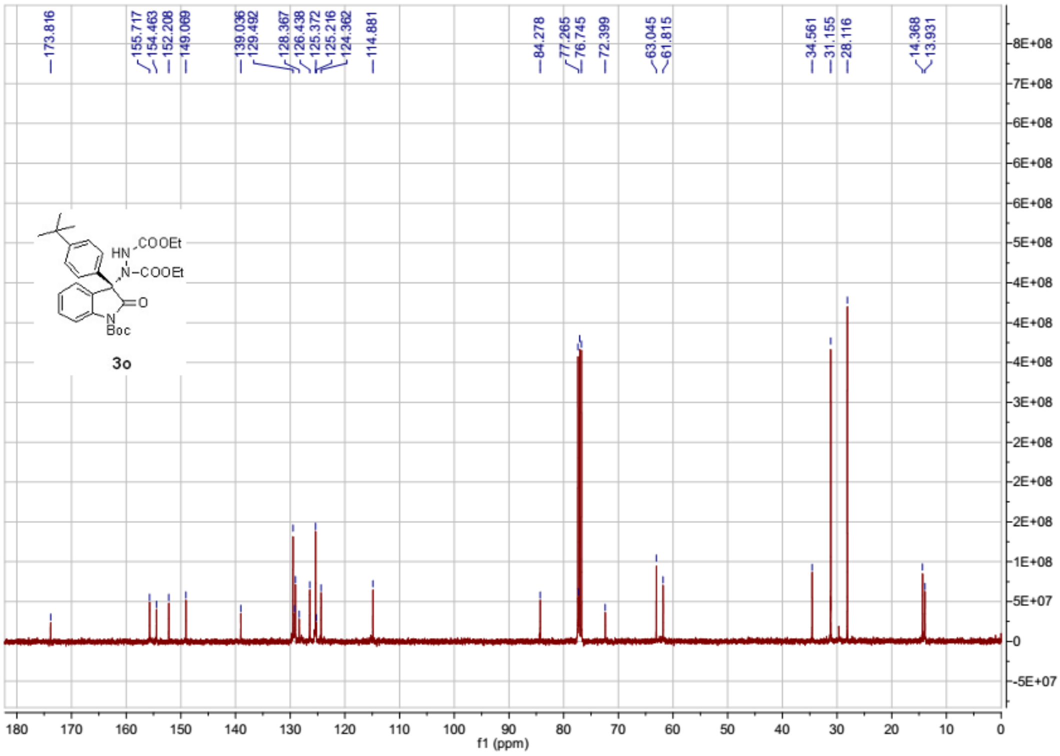

3o
f1 (ppm)

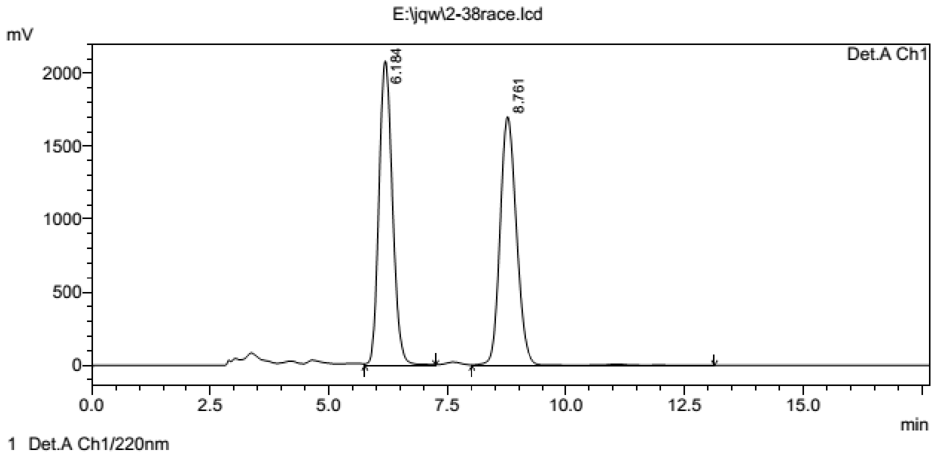

PeakTable

Detector A Ch1 220nm

Peak#	Ret. Time	Area	Height	Area %	Height %
1	6.184	41016557	2083283	50.205	55.028
2	8.761	40680877	1702548	49.795	44.972
Total		81697434	3785830	100.000	100.000

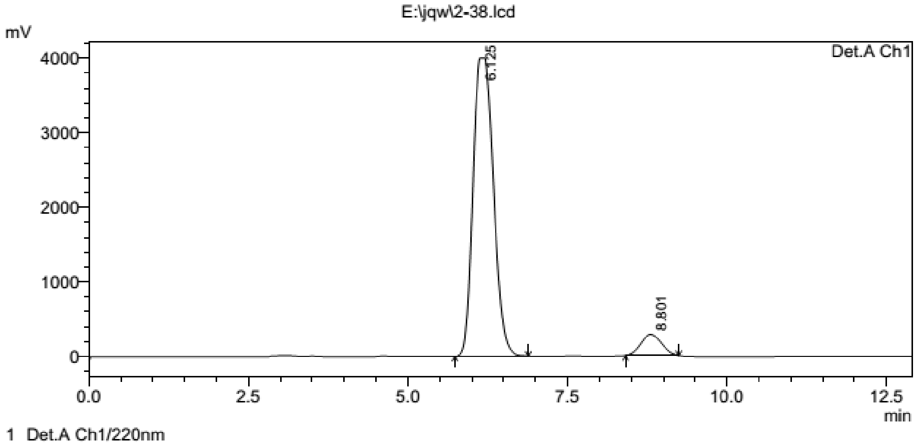

PeakTable

Detector A Ch1 220nm

Peak#	Ret. Time	Area	Height	Area %	Height %
1	6.125	88984294	3994438	93.286	93.366
2	8.801	6404631	283821	6.714	6.634
Total		95388926	4278259	100.000	100.000

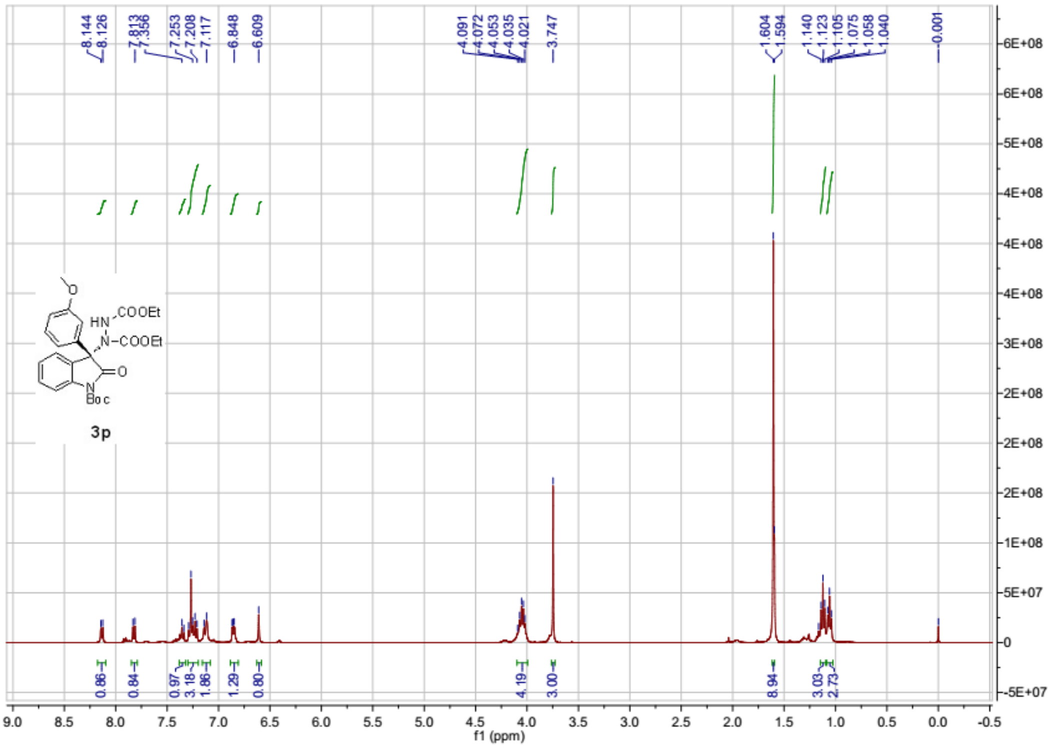

8.144
8.126
7.813
7.356
7.253
7.208
7.117
6.848
6.609
4.091
4.072
4.053
4.035
4.021
3.747
1.604
1.594
1.140
1.123
1.105
1.075
1.058
1.040
0.001
HN
COOEt
N–COOEt
O
N
Boc
3p
0.86
0.84
0.97
3.18
1.86
1.29
0.80
4.19
3.00
8.94
3.03
2.73
f1 (ppm)

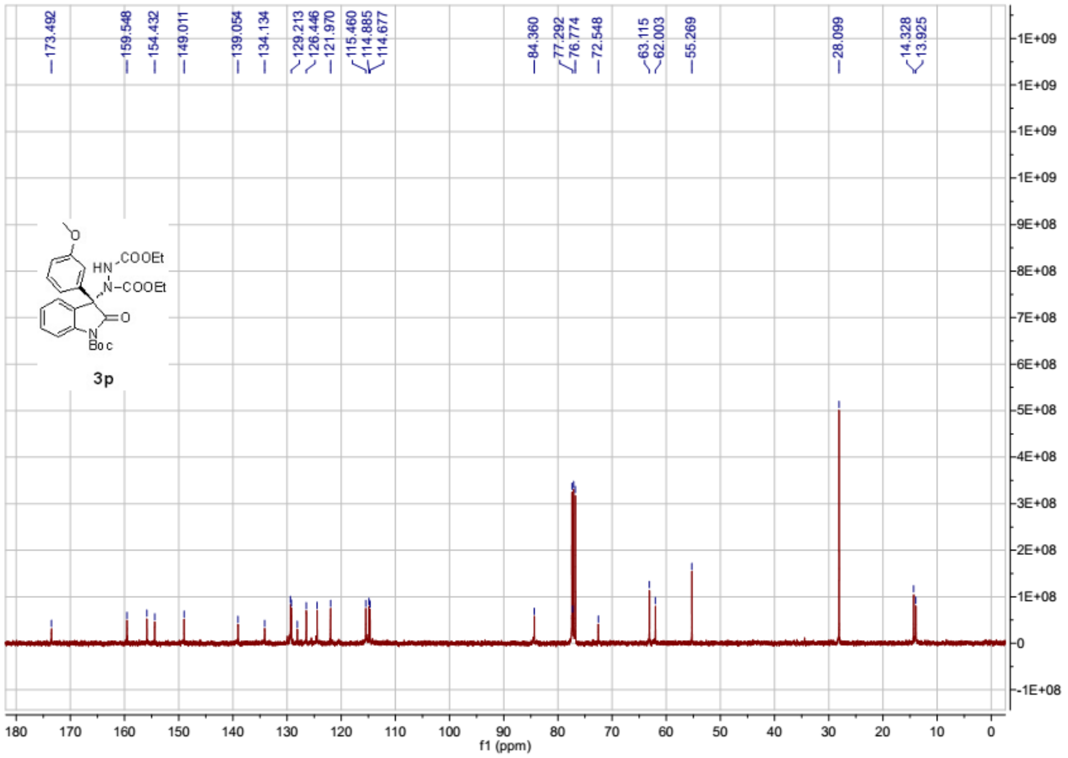

173.492
159.548
154.432
149.011
139.054
134.134
129.213
126.446
121.970
115.460
114.885
114.677
84.360
77.292
76.774
72.548
63.115
62.003
55.269
28.099
14.328
13.925
HN
COOEt
N–COOEt
O
N
Boc
3p
f1 (ppm)

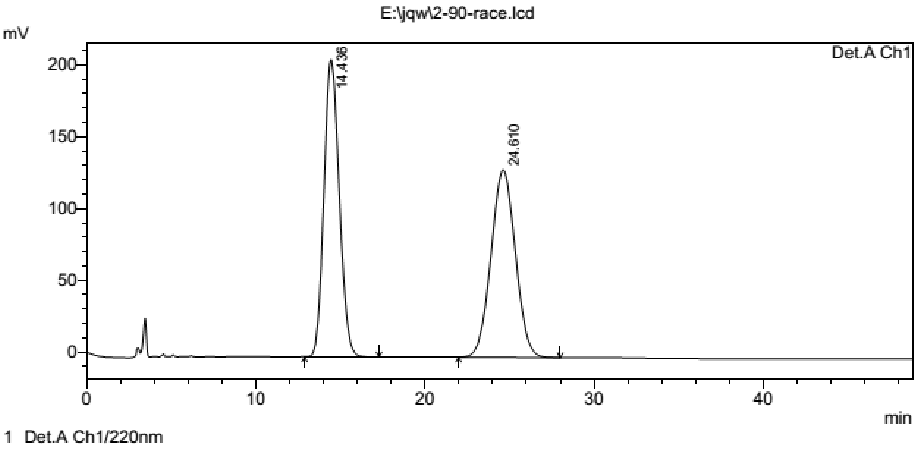

PeakTable

Detector A Ch1 220nm

Peak#	Ret. Time	Area	Height	Area %	Height %
1	14.436	13070480	207122	49.976	61.336
2	24.610	13083295	130563	50.024	38.664
Total		26153775	337686	100.000	100.000

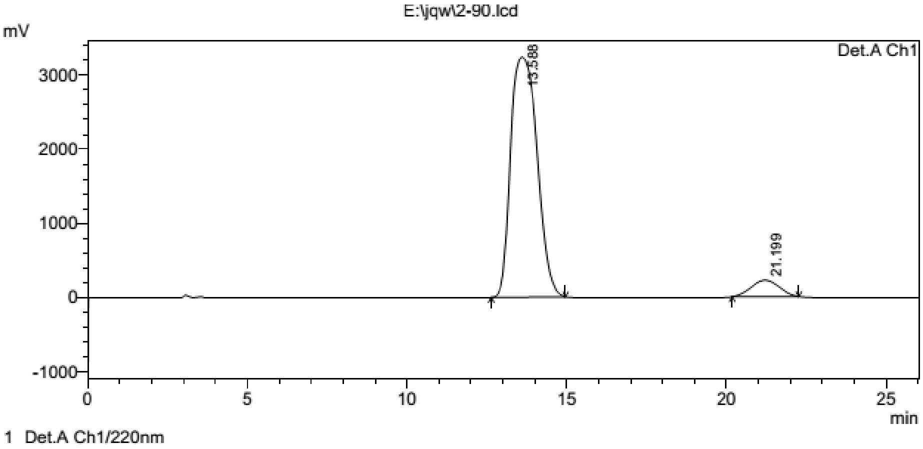

PeakTable

Detector A Ch1 220nm

Peak#	Ret. Time	Area	Height	Area %	Height %
1	13.588	186306141	3235417	93.352	93.698
2	21.199	13267508	217598	6.648	6.302
Total		199573650	3453015	100.000	100.000

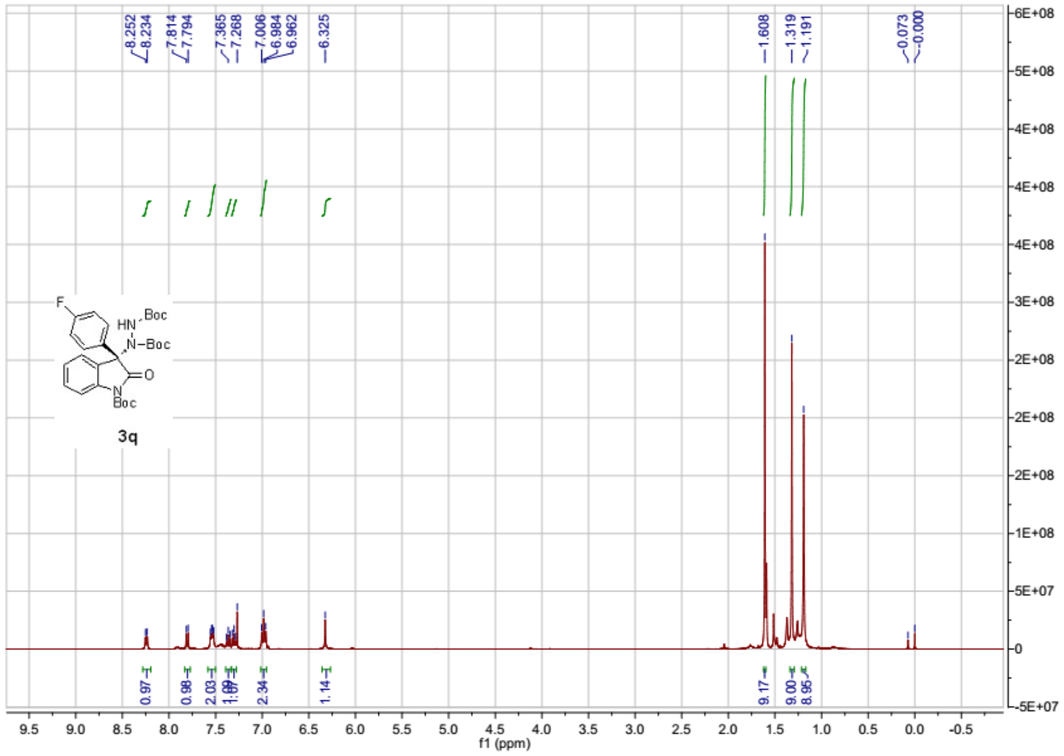

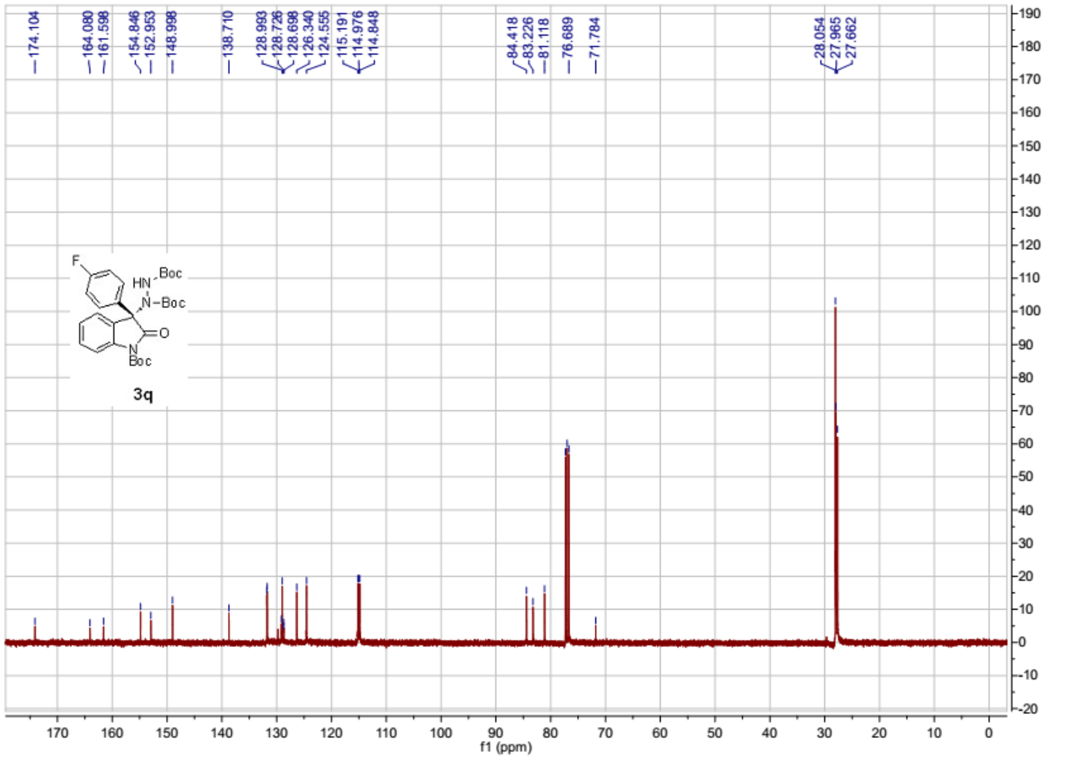

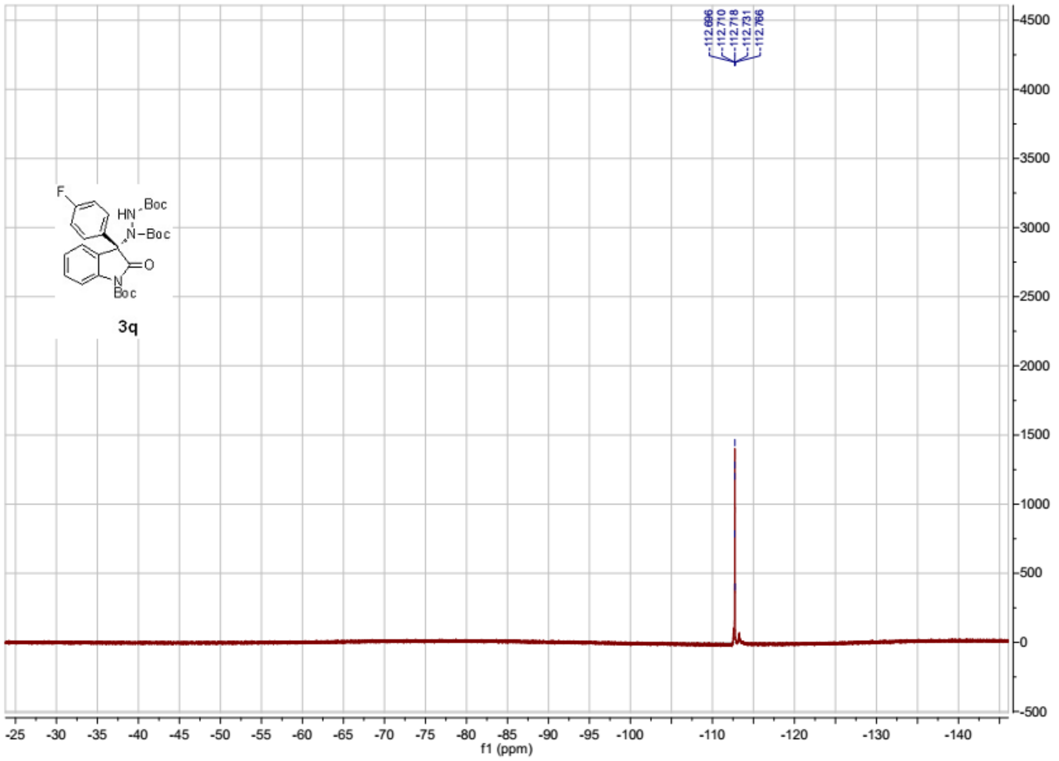

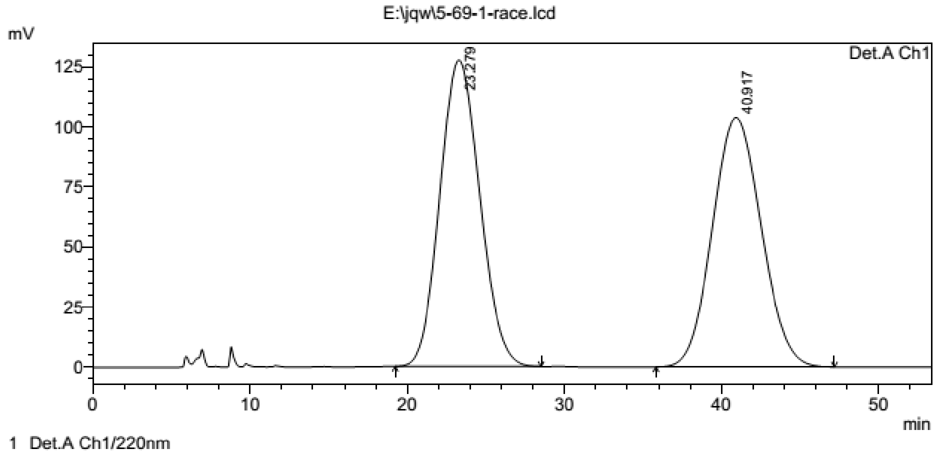

PeakTable

Detector A Ch1 220nm

Peak#	Ret. Time	Area	Height	Area %	Height %
1	23.279	22492093	127339	50.078	55.128
2	40.917	22421704	103647	49.922	44.872
Total		44913796	230986	100.000	100.000

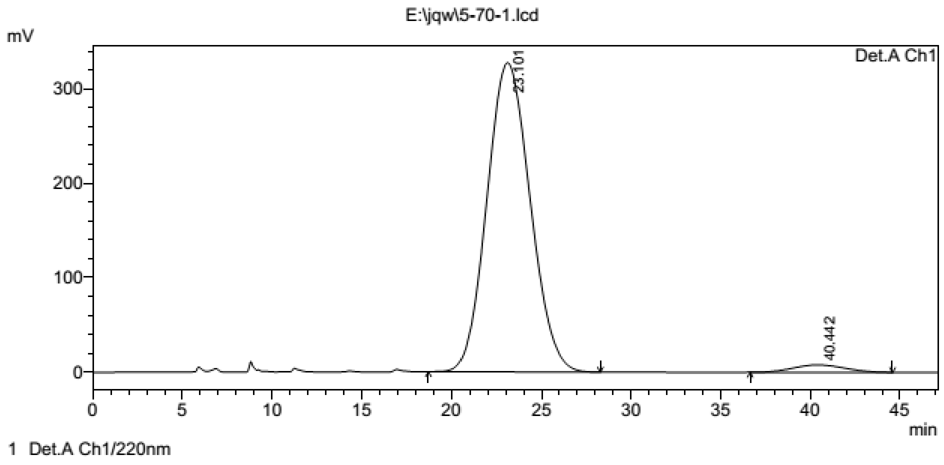

PeakTable

Detector A Ch1 220nm

Peak#	Ret. Time	Area	Height	Area %	Height %
1	23.101	54931756	327299	97.163	97.615
2	40.442	1603926	7995	2.837	2.385
Total		56535682	335295	100.000	100.000

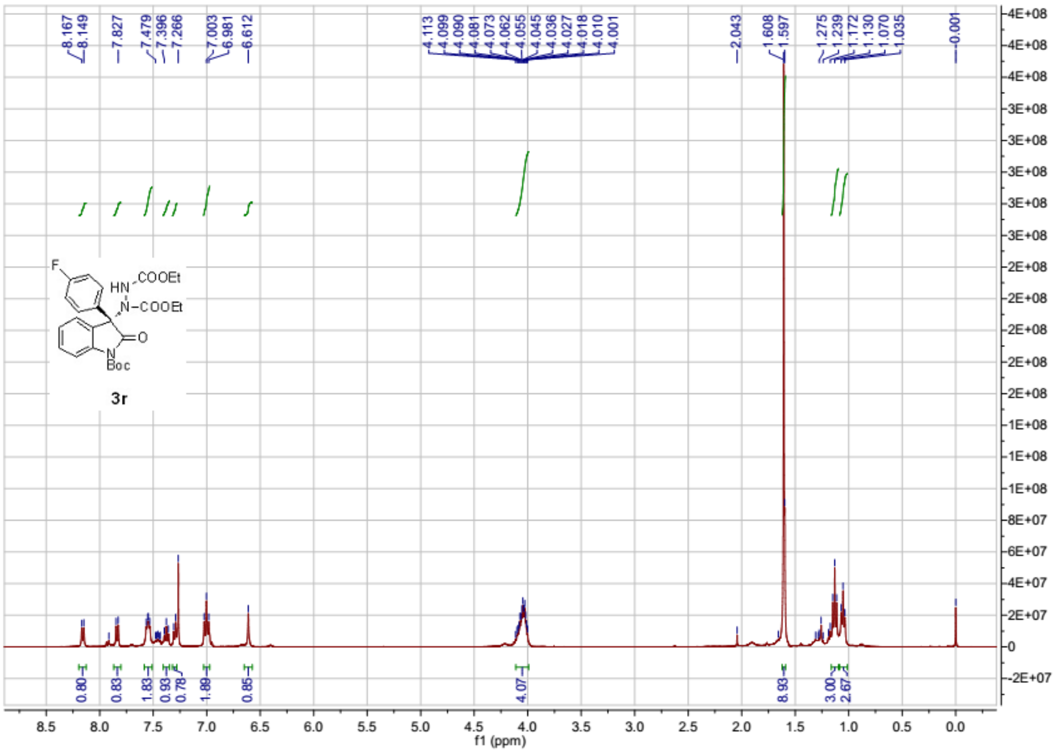

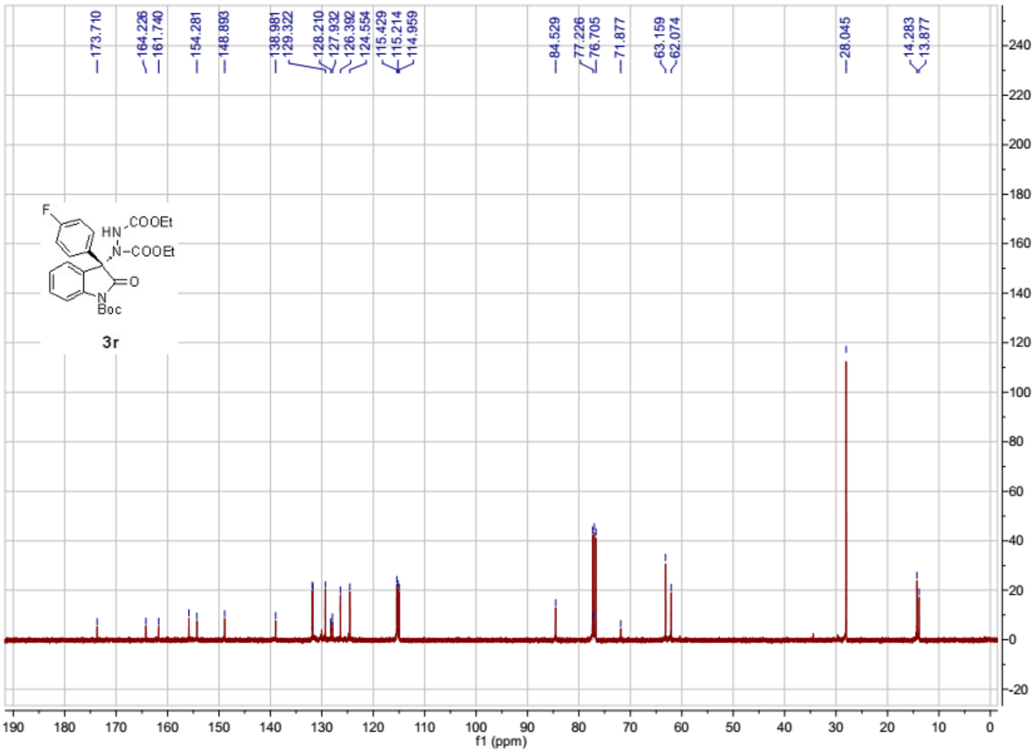
173.710
164.226
161.740
154.281
148.893
138.981
129.322
128.210
127.932
126.392
124.554
115.429
115.214
114.959
84.529
77.226
76.705
71.877
63.159
62.074
28.045
14.283
13.877
HN-COOEt
N-COOEt
F
O
N
Boc
3r
f1 (ppm)

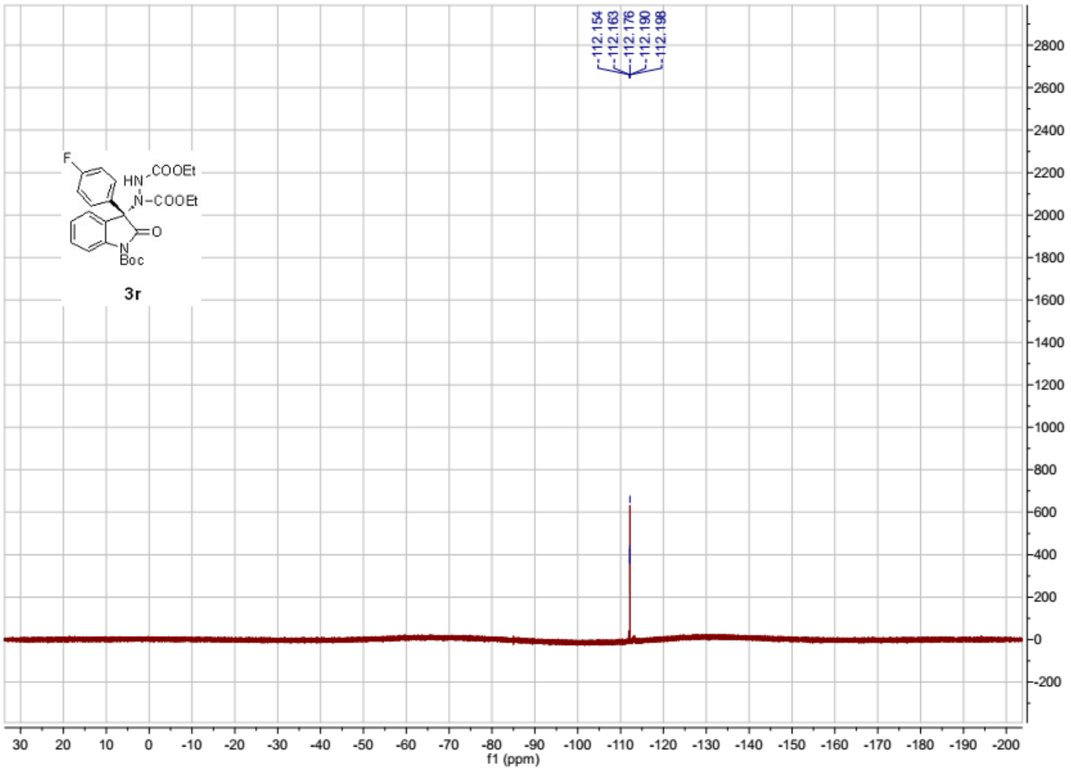
-112.154
-112.163
-112.176
-112.190
-112.198
HN-COOEt
N-COOEt
F
O
N
Boc
3r
f1 (ppm)

E:\jqw\2-44-race.lcd

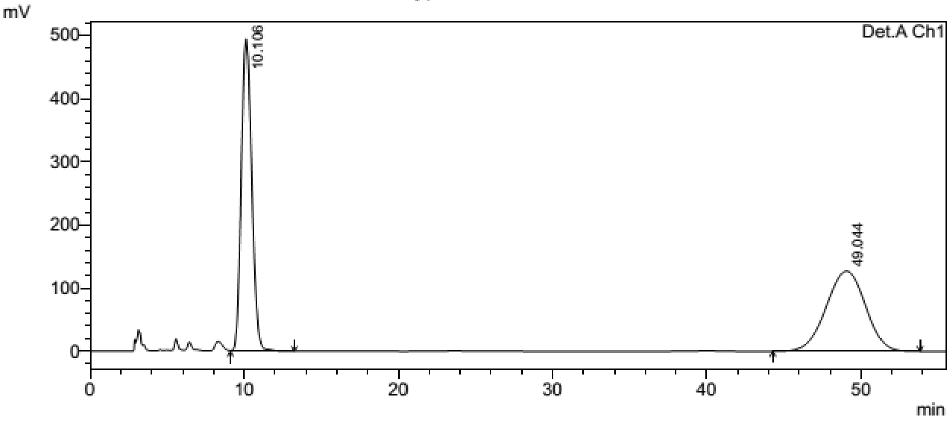

1 Det.A Ch1/220nm

PeakTable

Detector A Ch1 220nm

Peak#	Ret. Time	Area	Height	Area %	Height %
1	10.106	22795755	494762	50.185	79.504
2	49.044	22628097	127545	49.815	20.496
Total		45423852	622307	100.000	100.000

E:\jqw\2-44.lcd

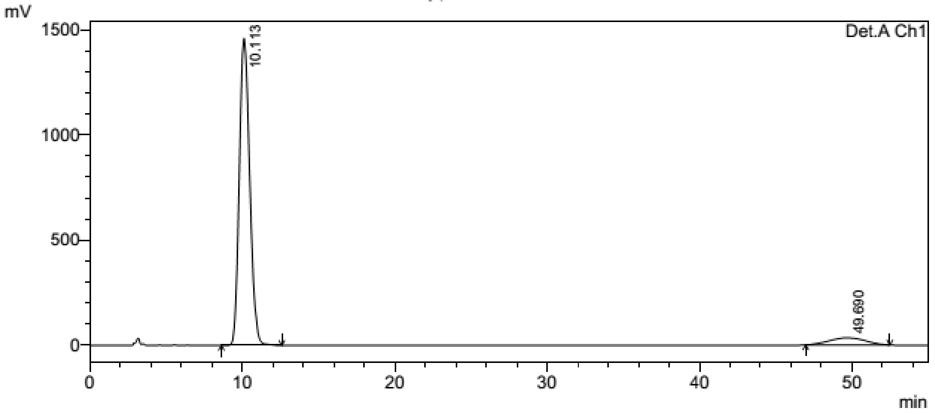

1 Det.A Ch1/220nm

PeakTable

Detector A Ch1 220nm

Peak#	Ret. Time	Area	Height	Area %	Height %
1	10.113	68532857	1459799	92.568	97.740
2	49.690	5501922	33761	7.432	2.260
Total		74034779	1493560	100.000	100.000

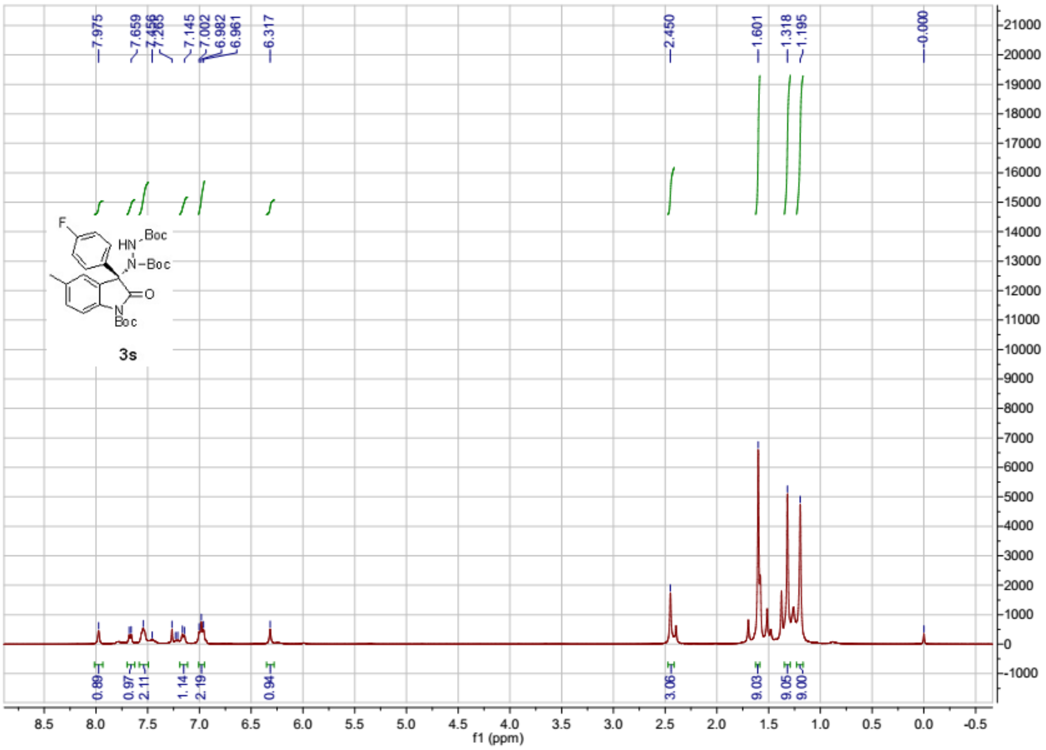
3s

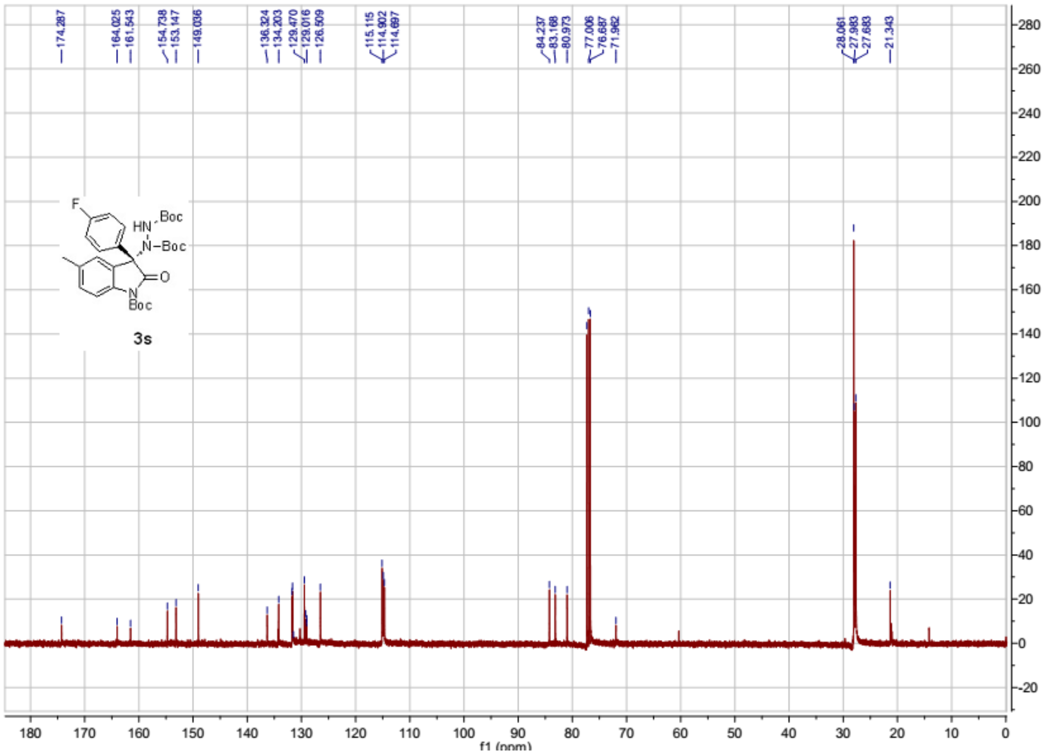
3s

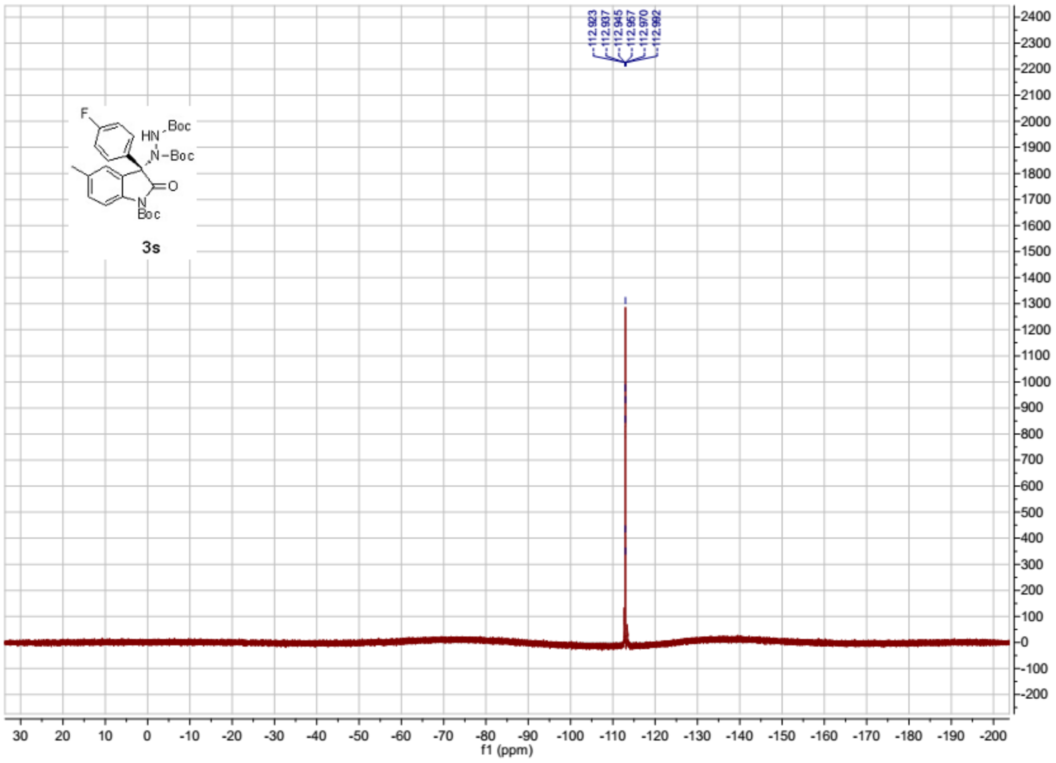

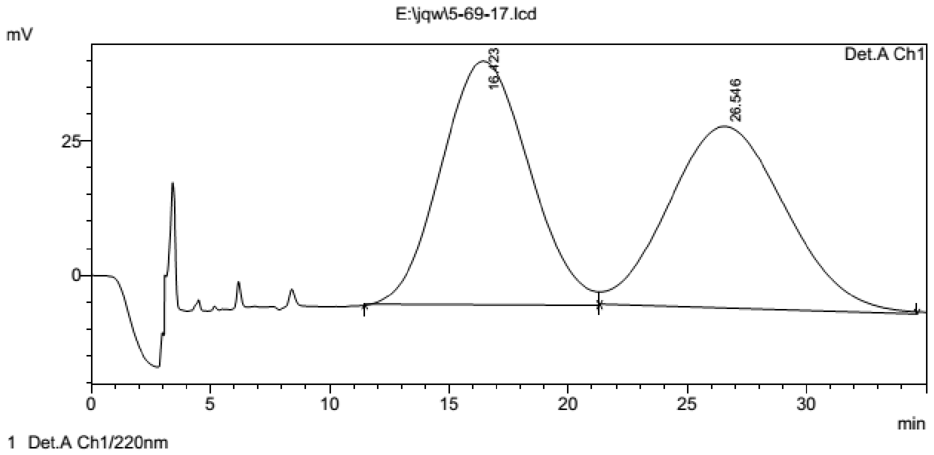

PeakTable

Detector A Ch1 220nm

Peak#	Ret. Time	Area	Height	Area %	Height %
1	16.423	11657586	45302	50.276	57.319
2	26.546	11529452	33732	49.724	42.681
Total		23187038	79034	100.000	100.000

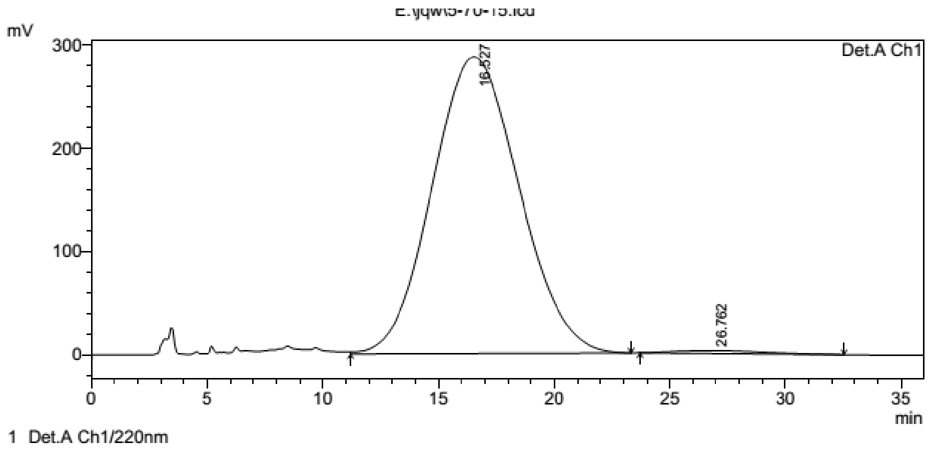

PeakTable

Detector A Ch1 220nm

Peak#	Ret. Time	Area	Height	Area %	Height %
1	16.527	75216472	287675	99.004	99.041
2	26.762	756507	2787	0.996	0.959
Total		75972979	290461	100.000	100.000

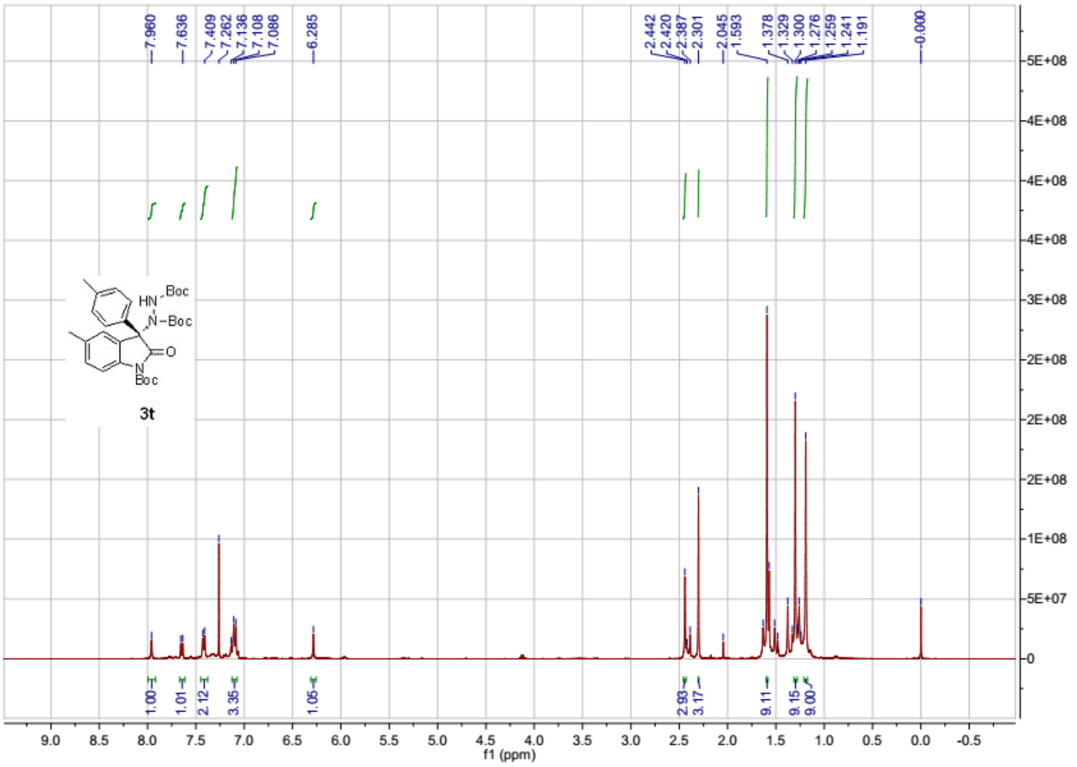

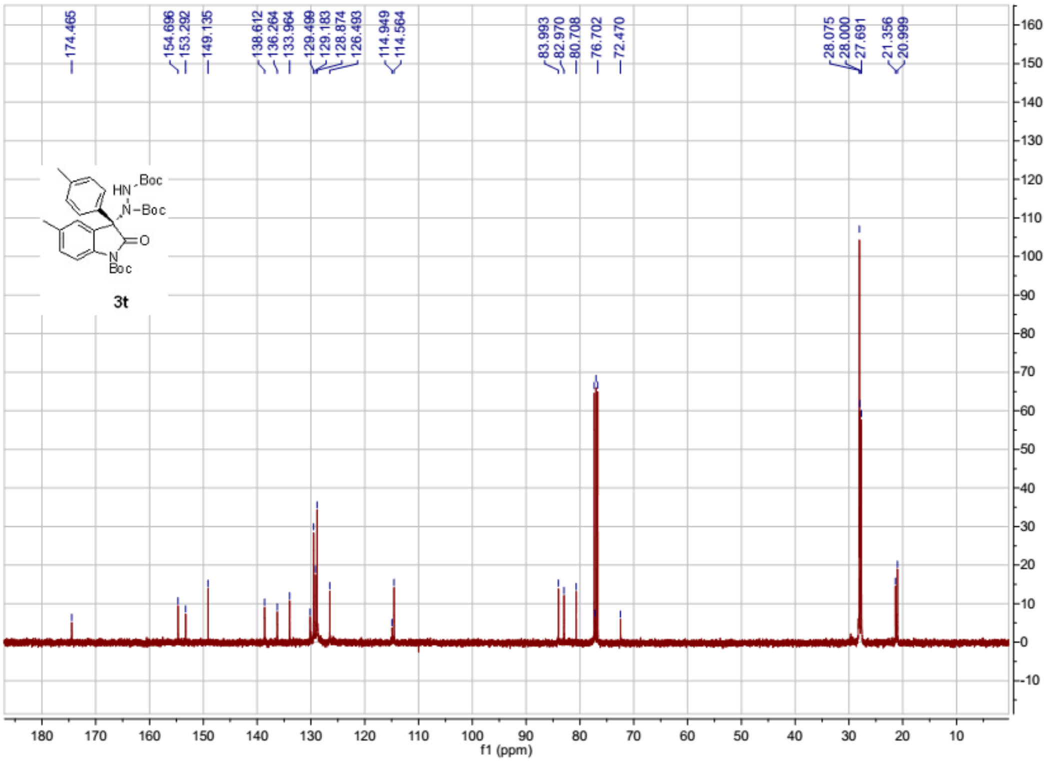

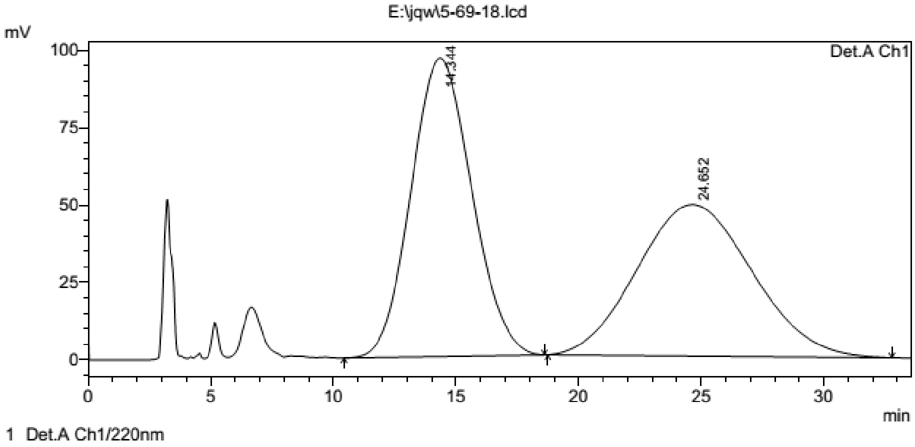

PeakTable

Detector A Ch1 220nm

Peak#	Ret. Time	Area	Height	Area %	Height %
1	14.344	16595116	96286	50.935	66.327
2	24.652	15985814	48883	49.065	33.673
Total		32580930	145169	100.000	100.000

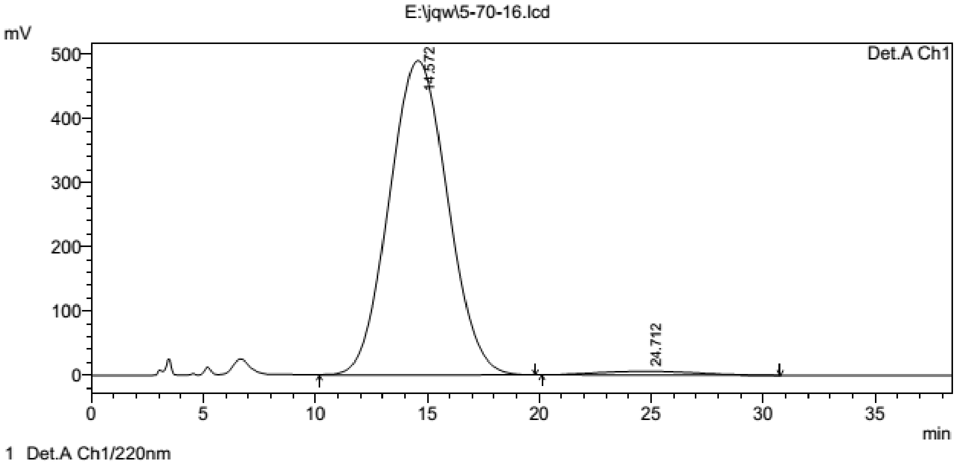

PeakTable

Detector A Ch1 220nm

Peak#	Ret. Time	Area	Height	Area %	Height %
1	14.572	88997599	490053	97.929	98.763
2	24.712	1882024	6135	2.071	1.237
Total		90879623	496188	100.000	100.000

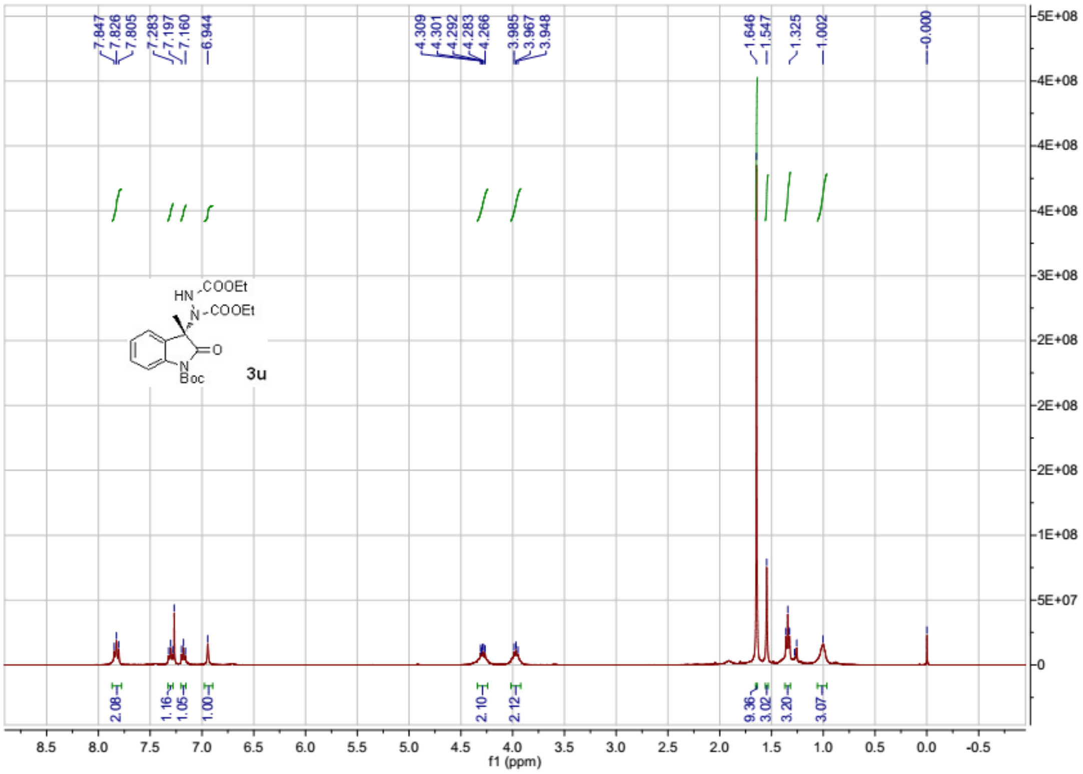

E:\jqw\2-59-race.lcd

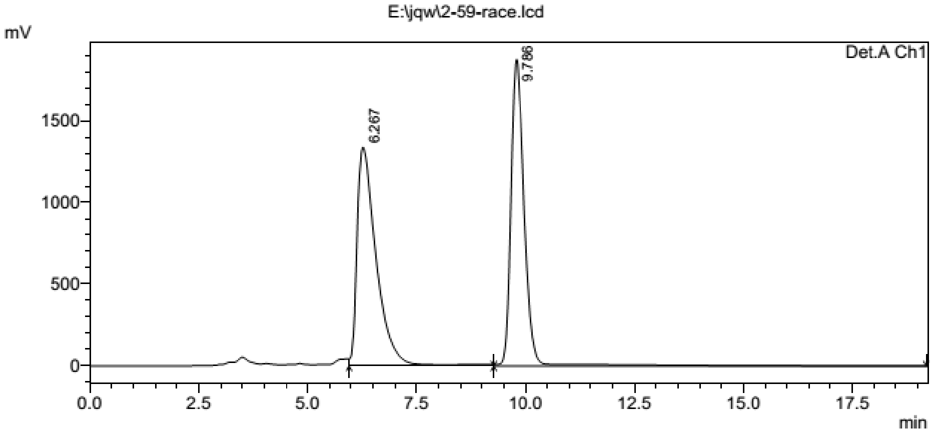

1 Det.A Ch1/220nm

PeakTable

Detector A Ch1 220nm

Peak#	Ret. Time	Area	Height	Area %	Height %
1	6.267	40829965	1335498	49.702	41.574
2	9.786	41319075	1876874	50.298	58.426
Total		82149040	3212372	100.000	100.000

E:\jqw\2-59r.t..lcd

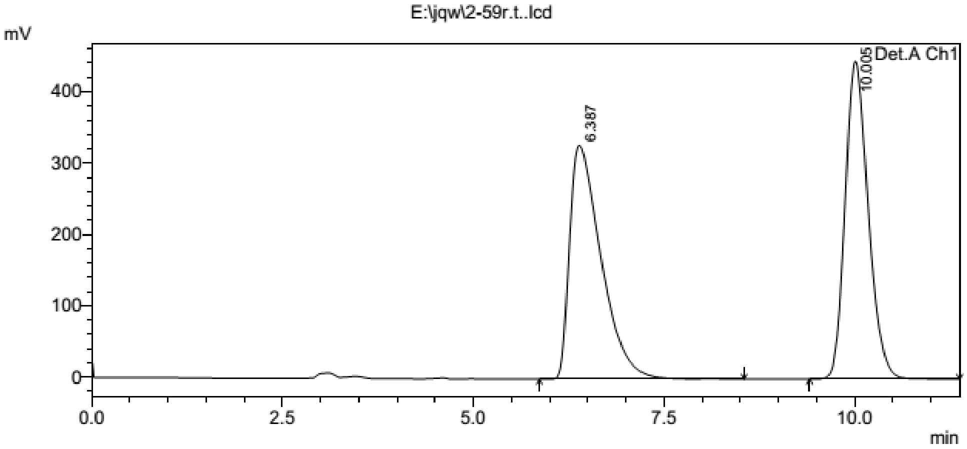

1 Det.A Ch1/220nm

PeakTable

Detector A Ch1 220nm

Peak#	Ret. Time	Area	Height	Area %	Height %
1	6.387	9398527	327604	50.083	42.391
2	10.005	9367452	445209	49.917	57.609
Total		18765980	772813	100.000	100.000

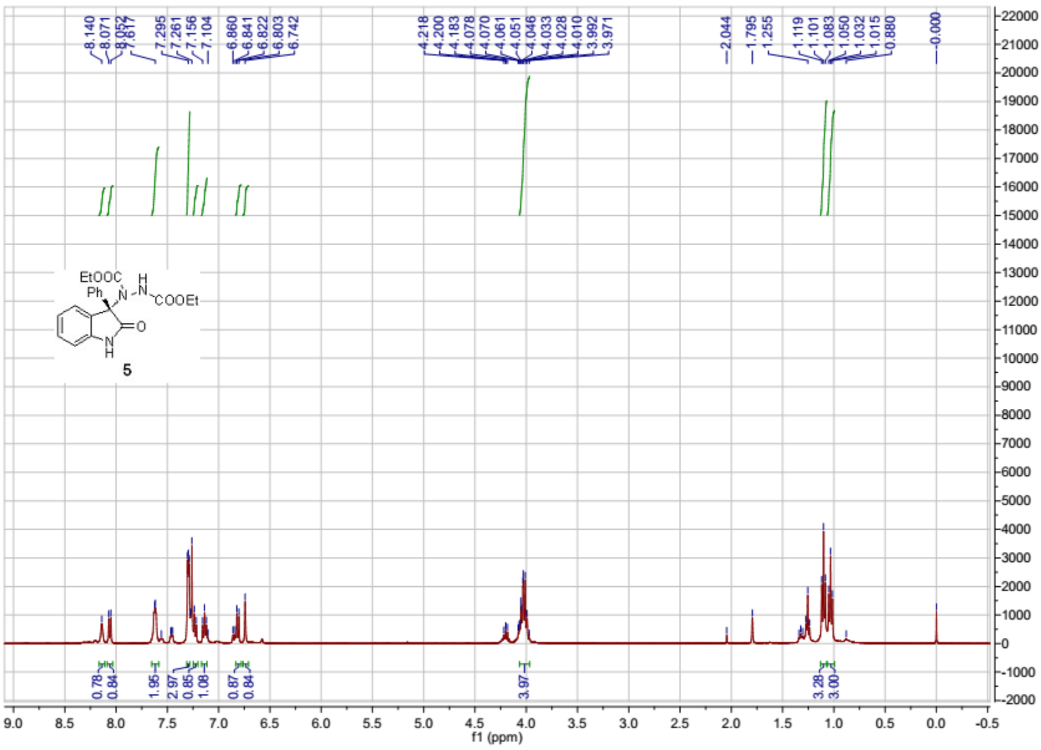

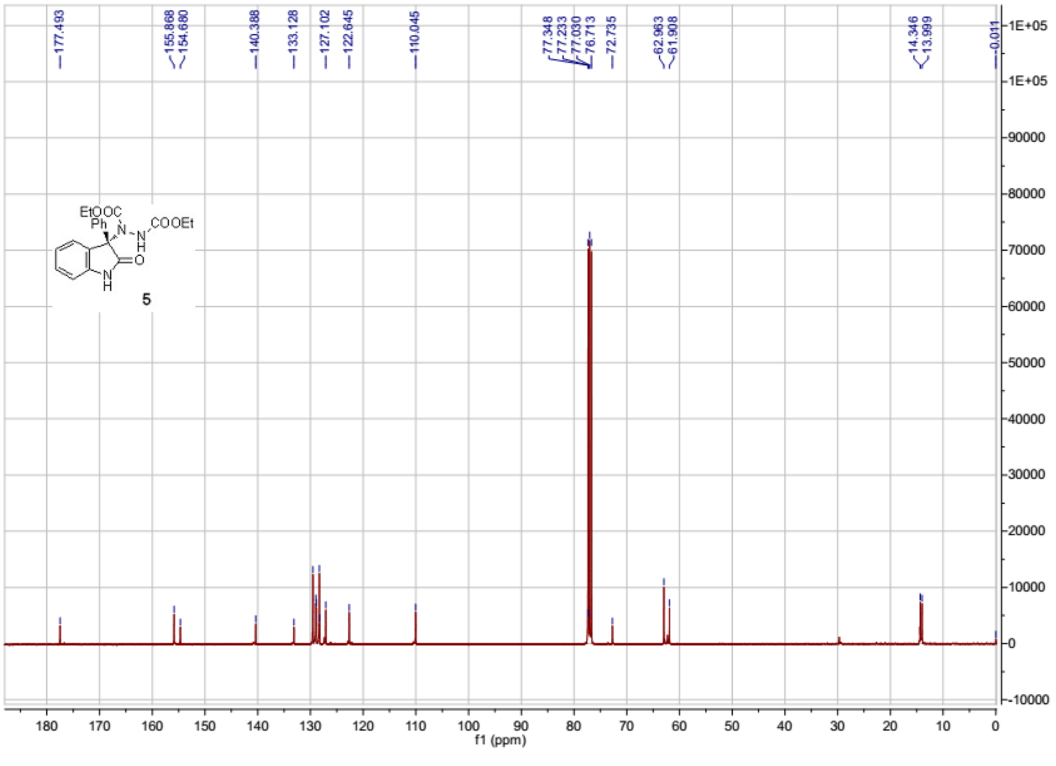

1880 JQW-3-7+- IA 73 214 0.7

Sample Name:	**JQW-3-7+- IA 73 214 0.7**	*Injection Volume:*	**5.0**
Vial Number:	**RE1**	*Channel:*	**UV_VIS_1**
Sample Type:	**unknown**	*Wavelength:*	**214**
Control Program:	**WXL-2014**	*Bandwidth:*	**n.a.**
Quantif. Method:	**WXL**	*Dilution Factor:*	**1.0000**
Recording Time:	**2014/10/22 16:27**	*Sample Weight:*	**1.0000**
Run Time (min):	**25.35**	*Sample Amount:*	**1.0000**

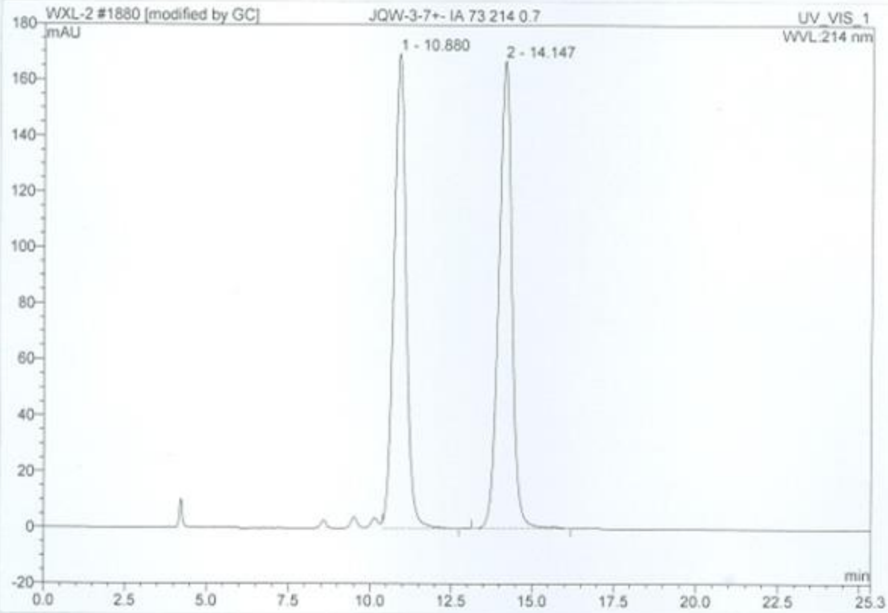

No.	Ret.Time min	Peak Name	Height mAU	Area mAU*min	Rel.Area %	Amount	Type
1	10.88	n.a.	169.806	73.957	48.31	n.a.	MB*
2	14.15	n.a.	167.387	79.135	51.69	n.a.	BMB*
Total:			337.193	153.093	100.00	0.000	

1881 JQW-3-7 IA 73 214 0.7

Sample Name:	JQW-3-7 IA 73 214 0.7	Injection Volume:	5.0
Vial Number:	RE2	Channel:	UV_VIS_1
Sample Type:	unknown	Wavelength:	214
Control Program:	WXL-2014	Bandwidth:	n.a.
Quantif. Method:	WXL	Dilution Factor:	1.0000
Recording Time:	2014/10/22 16:55	Sample Weight:	1.0000
Run Time (min):	17.12	Sample Amount:	1.0000

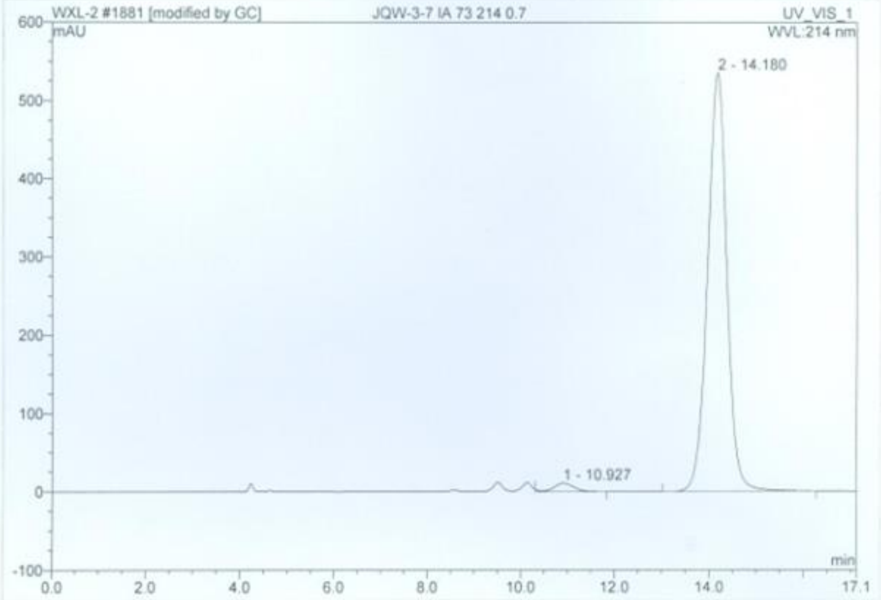

No.	Ret.Time min	Peak Name	Height mAU	Area mAU*min	Rel.Area %	Amount	Type
1	10.93	n.a.	10.321	5.066	1.97	n.a.	MB*
2	14.18	n.a.	535.506	251.494	98.03	n.a.	BMB*
Total:			545.827	256.560	100.00	0.000	
